# Food-based indexes and their association with dietary inflammation

**DOI:** 10.1016/j.advnut.2025.100400

**Published:** 2025-03-04

**Authors:** Gynette L Reyneke, Kelly Lambert, Eleanor J Beck

**Affiliations:** 1 School of Health Sciences, Faculty of Medicine & Health, University of New South Wales, Sydney, New South Wales, Australia; 2 School of Medical, Indigenous, and Health Sciences, Faculty of Science, Medicine and Health, University of Wollongong, Wollongong, New South Wales, Australia

**Keywords:** Diet quality index, chronic inflammation, nutrition, noncommunicable disease, dietary patterns, scoping review

## Abstract

Chronic inflammation is associated with an increased risk of noncommunicable diseases, prompting an intensified interest in the diet-disease relationship for modulating inflammation. Diet quality indexes are widely used to quantify dietary patterns. However, the optimal tool for assessing dietary quality in relation to chronic inflammation remains unclear. The objective of this study was to synthesize the literature on food-based diet quality indexes and their association with chronic inflammation. A systematic scoping review of scientific databases was conducted from inception to March 2024. Studies describing the development and validation of original dietary inflammatory indexes or assessed associations between established indexes and inflammatory biomarkers were included. Studies that predominantly focused on nutrient-based indexes were excluded. Forty-three food-based indexes, evaluated across 65 studies, were categorized into 4 distinct groups based on dietary patterns (*n* = 18), dietary guidelines (*n* = 14), dietary inflammatory potential (*n* = 6), and therapeutic diets (*n* = 5). Established indexes based on the Mediterranean diet and dietary guidelines were the most extensively utilized, demonstrating inverse associations with several inflammatory biomarkers across diverse populations. The Anti-Inflammatory Diet Index, Dietary Inflammation Score, and Empirical Dietary Inflammatory Index were identified as robust, empirically derived indexes to assess diet quality based on their inflammatory potential. The dietary composition of the evaluated indexes ranged from 4 to 28 dietary components, with fruits, vegetables, whole grains, and legumes consistently classified as favorable, whereas red/processed meats and added sugars were unfavorable. This scoping review identified several promising food-based indexes for assessing inflammation-related diet quality. Methodological variations and inconsistencies in algorithms underscore the need for further validation across diverse populations. Future research should consider the scoring methods, dietary composition, and validated inflammatory biomarkers when selecting indexes to evaluate diet-inflammation associations. Understanding the characteristics that underpin these indexes informs their application in nutrition research and clinical practice.

**Statement of Significance**

To our knowledge, this review provides the first comprehensive synthesis of food-based indexes assessing diet quality in relation to biomarkers of chronic inflammation. It identifies commonalities and discrepancies in the foods and algorithms utilized in the development and application of such indexes, highlighting the need for broader validation across diverse populations.

## Introduction

Chronic low-grade inflammation is associated with the onset and acceleration of age-related diseases [1,2] and an elevated risk of several noncommunicable diseases (NCDs) [3,4]. The WHO has identified NCDs as a primary threat to global health and sustainability [5]. The global incidence of inflammation-related chronic diseases such as cardiovascular disease (CVD), type 2 diabetes mellitus, and certain cancers is projected to rise over the next 3 decades [5,6]. Efforts to reduce premature mortality by 30% by 2030 through the prevention and treatment of NCDs have intensified interest in the diet-disease relationship [5,7].

Traditionally, nutritional epidemiology has examined the relationship between single nutrients or foods and risk of chronic disease. However, this reductionist approach of attributing specific effects to individual foods overlooks the complexities of whole diets with multiple nutrients and nonnutrients [8,9]. In light of this, nutrition research has shifted to a holistic approach for evaluating the role of whole dietary patterns in the diet-disease relationship [10]. Dietary pattern analysis considers potential correlations and synergistic effects of consuming combinations of foods and nutrients [10,11]. This approach also elucidates how increased consumption of certain foods (e.g., red and processed meat) might be associated with reduced intake of others (e.g., vegetables and legumes) [12,13]. Understanding the intricacies of dietary patterns is increasingly recognized in nutrition research and public health [14,15]. For example, healthy dietary patterns rich in plant foods are associated with low-grade inflammation [[16], [17]] and improved health outcomes [7,18]. Current research seeks to elucidate the relationship between the inflammatory potential of diet and various health outcomes, including CVD [19], neurodegenerative diseases [20,21], inflammatory bowel disease (IBD) [22,23], cancer [24,25], and all-cause mortality [7,18].

Researchers use indirect methods such as diet quality indexes to quantify dietary patterns [26]. These dietary indexes are either based on theoretically defined dietary patterns informed by current nutritional knowledge and guidelines or on empirical dietary patterns derived through statistical techniques such as principal component analysis and cluster analysis [26]. Numerous dietary indexes have been developed and utilized to investigate the relationship between diet and inflammation. For instance, the Empirical Dietary Inflammatory Index (EDII), a food-based index developed through data-driven methods, evaluates the inflammatory potential of diets and has been extensively used in health outcome studies [[27], [28], [29]]. The EDII reflects actual dietary patterns, offering a realistic representation of dietary intake that is more translatable to public health messaging. However, its generalizability may be limited as the findings are derived from niche populations, such as specific health conditions, age groups, or geographic regions, which may not represent the broader population. Additionally, inconsistencies in the association between these dietary indexes and inflammatory biomarkers across studies may arise from variations in dietary composition, such as differences in the types and quantities of foods or nutrients included or inflammatory biomarkers assessed.

To optimize translational value and align with contemporary nutritional research, this scoping review focused on food-based indexes [10,11]. Extensive research has generated numerous dietary indexes; therefore, identification of an optimal tool for assessing dietary inflammation remains indeterminate. This scoping review aimed to systematically evaluate food-based indexes and their association with chronic inflammation by examining the following:*1*) methodologies for developing and validating original dietary inflammatory indexes, *2*) associations between established dietary indexes and inflammation, and *3*) dietary composition and scoring structure of indexes used to measure dietary inflammation. This synthesis informs future research in selecting the optimal index for specific inquiries by enhancing the understanding of available inflammation-related food-based indexes and their adaptability for various research objectives and populations [30].

## Methods

This scoping review followed the Joanna Briggs Institute (JBI) Manual for Evidence Synthesis methodological guidance for scoping reviews [30,31] and is reported in accordance with the Preferred Reporting Items for Systematic Reviews extension for Scoping Reviews guidelines [32] (Supplemental Table 1). The protocol was registered in the Open Science Framework (https://doi.org/10.17605/OSF.IO/C4FB2).

### Eligibility criteria

For this scoping review, a food-based index was defined as an evidence-based tool used to apply a quantitative score to dietary intake data derived from structured dietary assessment methods (e.g., food frequency questionnaire [FFQ], 24-h recall, and 3-D food diary). The term “dietary components” refers to dietary items contained in the index, including food groups, foods, beverages, and nutrients. Peer-reviewed studies were included if they statistically assessed a predominantly food-based index for biomarkers of chronic inflammation. This review included studies that either *1*) described the development and validation of a dietary inflammatory index to predict the inflammatory potential of diet in a population or *2*) described the application of an established dietary index to assess the associations between dietary intake and biomarkers of chronic inflammation. The eligibility criteria, including the population, concept, and outcomes of interest, context, and study design, are presented in Table 1. To provide a comprehensive inventory of inflammation-related dietary indexes, articles were not excluded based on their methodological quality. Additionally, in accordance with the scoping review methodology, the validity and quality of the studies and indicators utilized were not assessed [30,31].

**TABLE 1:** Eligibility criteria for included studies.

	Inclusion criteria	Exclusion criteria
Population	Human participants	*1*) human participants with acute inflammatory conditions; *2*) animal populations or in vitro studies
Concept	Studies that *1*) described the development and validation of a dietary inflammatory index to predict the inflammatory potential of diet in a population or *2*) described the application of an established dietary index to assess the associations between dietary intake with biomarkers of chronic inflammation. *Outcomes of interest* *1*) methods of development and/or validation; *2*) dietary components included in indexes and the scoring structure; *3*) statistical analyses conducted to assess the association between index and biomarkers of chronic inflammation.	Development, validation, or utilization of a predominantly nutrient-based index/score *Outcomes:* 1) included any lifestyle component that could not be separated from the overall score; 2) data on chronic inflammatory biomarkers for the population was not included; 3) did not perform statistical analyses to assess association between dietary index and biomarkers of chronic inflammation; 4) inadequately described key characteristics of dietary index.
Context	No restrictions imposed on geographic location, culture, ethnicity, or socioeconomic factors	Acute care
Study design	Original/ primary research study, including observational (case-control, cohort, or cross-sectional) studies or intervention trials	*1*) abstracts; *2*) review studies or meta-analyses; *3*) non-peer-reviewed studies

### Search strategy

A systematic search of the literature was conducted in scientific databases Medline (EBSCO), CINAHL (EBSCO), Cochrane CENTRAL, PubMed, and Embase (Ovid) from inception to March 2024. The search was restricted to peer-reviewed studies published in English language. The search strategy contained free-text search terms and related controlled vocabulary terms pertaining to the research objectives as follows: dietary index OR diet quality index OR eating index OR diet score OR anti-inflammation dietary index OR anti-inflammation diet score AND inflammation OR inflammatory OR anti-inflammation OR anti-inflammatory OR inflammation mediators OR interleukin OR c-reactive protein OR tumor necrosis factor OR adiponectin OR cytokine. The reference lists of eligible publications were manually checked for additional relevant studies. Supplemental Table 2 provides the full search strategy.

### Study screening

The identified articles were exported to Covidence (Covidence systematic review software, Veritas Health Innovation), available at www.covidence.org. Duplicates were removed, and data were screened for study selection [33]. Authors (GR, KL, and EB) independently performed title and abstract screening of a random sample (20%) of articles, in duplicate [34,35]. A minimum consensus of 80% was achieved, and the remaining titles and abstracts were reviewed by a single author (GR) [36]. The identified articles were progressed for full-text review and independently screened in duplicate, according to the eligibility criteria by the authors (GR, KL, and EB). Any disagreements were resolved through consensus.

### Data extraction

Data extraction templates were developed in accordance with the JBI Manual for Evidence Synthesis Guidelines [30,31]. Data extraction was performed by one reviewer (GR), and a random sample (20%) of the data was extracted and verified for accuracy by a second reviewer (EB and KL) [34]. The extracted data were tabulated as follows: index, reference, country, funding source, population characteristics, study design, and source of data, dietary assessment method, inflammatory biomarkers, statistical analyses, assessment of variables, index dietary components, scoring methodology to assess food group intake, such as population-specific percentile cut-offs (e.g., median or tertiles), normative cut-offs (evidence-based diet-health associations), and study findings (associations between index and inflammatory biomarkers). Specific to the original dietary inflammatory indexes, the following data were extracted and tabulated: a methodology for index development and validation, basis of index (e.g., derived from national food-based dietary guidelines, traditional dietary patterns), rationale for dietary components included in the index, and study limitations.

### Synthesis of results

Narrative synthesis was conducted to provide a qualitative and descriptive summary of the evidence from the included studies. The key characteristics of original dietary inflammatory indexes were presented according to their methodology for development, scoring, and validation to provide an overview and scope of the key criteria researchers should consider when selecting an index. Studies that utilized established indexes, including those not specifically designed to assess dietary inflammation, were grouped according to the basis of the index. This approach facilitated the critical interpretation of each index.

## Results

**FIGURE 1:**
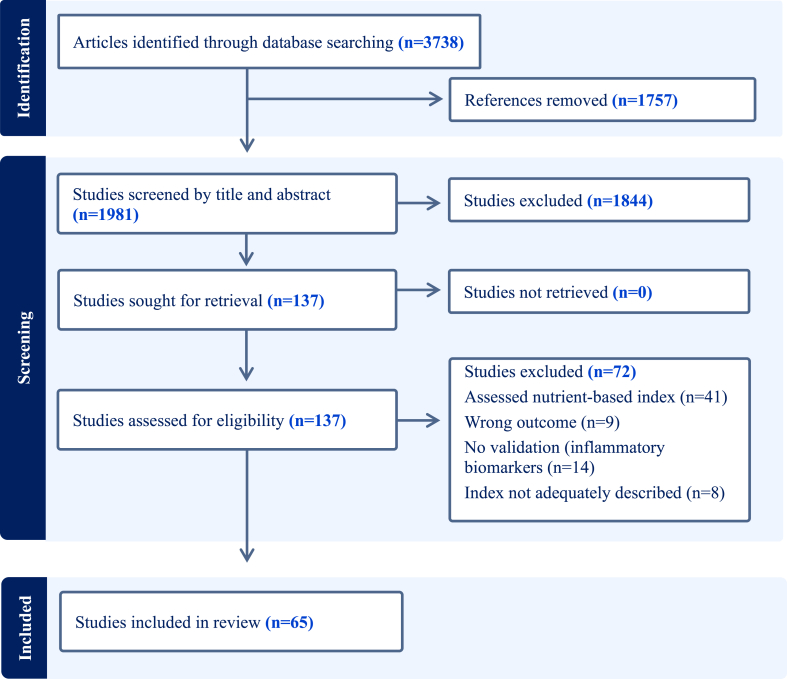
PRISMA flow diagram for the selection of articles that assessed dietary indexes in relation to inflammatory biomarkers.

A comprehensive search of scientific databases retrieved 3738 articles. After removing duplicates, the 1981 remaining articles were subjected to title and abstract screening (Figure 1). In total, 137 articles progressed to full-text review, and a further 72 articles were excluded, including those that evaluated nutrient-based dietary indexes (*n* = 41). Finally, 65 studies that assessed associations between food-based indexes and biomarkers of chronic inflammation were identified for inclusion.

### Study characteristics

The studies included in this review were conducted across a diverse range of geographic regions, including Europe (*n* = 24), North America (*n* = 22), Australia (*n* = 5), Asia (*n* = 5), the Middle East (*n* = 6), and South America (*n* = 4). Among the 65 reviewed studies, participants were typically middle-aged to older adults, although few studies included children and/or adolescents [[37], [38], [39]]. Although most studies included both sexes, some focused exclusively on females (*n* = 11) [[40], [41], [42], [43], [44], [45], [46], [47], [48], [49], [50]] or males (*n* = 4) [[51], [52], [53], [54]]. A total of 43 food-based indexes were identified, including 5 original dietary inflammatory indexes and 38 established diet quality indexes. A summary of the food-based indexes is presented in Table 2 [19,25,28,29,37,[39], [40], [41], [42],[44], [45], [46], [47], [48], [49], [50],^53^,^54^,^55^].

**TABLE 2:** Dietary components and scoring structure of food-based indexes used to assess dietary inflammation.

Index (reference for version used)	Secondary studies	Dietary composition of index/score		Scoring	Total score (range)	Basis of cut-off values
		No.	Dietary components			
Indexes based on dietary inflammatory potential						

AIDI-20 Kaluza 2018 [44]	N/A	20	Anti-inflammatory foods: total fruits and vegetables, dry fruits, beans/lentils, tea, herbal tea, coffee, whole-grain bread, breakfast cereal, low-fat cheese, olive/canola oil, nuts, linseeds, chocolate, red wine, beer. Proinflammatory foods: unprocessed red meat, processed red meat, organ meats, chips, SSB.	AIDI-20 was categorized into quintiles, and the lowest quintile (Q1) was used as the reference group. • Anti-inflammatory foods: 1 point assigned for consumption ≥ cut-off value for each dietary component. • Proinflammatory foods: 1 point assigned for no consumption < cut-off value for each dietary component. Total score is calculated by summing the points for all food groups. A higher score indicates a more anti-inflammatory diet, whereas a low score indicates a more proinflammatory diet	(0–20)	Cut-off values are based on an empirical approach. Optimal cut-off values that were strongly associated with CRP
DIS Byrd 2019 [55]	N/A	19	Anti-inflammatory foods: apples/berries, other fruits/juice, dark yellow vegetables/fruits, leafy greens/cruciferous vegetables, tomatoes, other vegetables, legumes, nuts, fish, poultry, high-fat dairy, low-fat dairy, coffee/tea (nutrients: supplement score). Proinflammatory foods: refined grains/starches, red/organ meats, processed meats, foods with added sugars, other added fats.	DIS was categorized into quintiles where the highest quintile (Q5) indicated a more proinflammatory diet and the lower quintile (Q1)—a more anti-inflammatory diet. DIS was calculated for each participant by multiplying intake of each dietary component by its weighting (β-coefficient) and then summing the weighted components to derive an overall DIS score. • Anti-inflammatory foods: each dietary component was assigned a negative weighting • Proinflammatory foods: each dietary component was assigned a positive weighting A positive overall score indicates a more proinflammatory diet	(-ve to +ve values)	Weights (β coefficient) based on their strength of association with inflammation
EDII/EDIP Tabung 2016 [49]	Aroke 2020 [51]; Byrd 2019 [55]; Tabung 2017 [29]; Vagianos 2021 [56]	18	Anti-inflammatory foods: fruit juice, dark yellow vegetables, leafy green vegetables, tea, coffee, pizza, snacks, wine, beer. Proinflammatory foods: tomatoes, other vegetables, refined grains, fish, red meat, meat, high energy beverages, low energy beverages.	The EDII score was obtained by summing the weighted number of daily serves, and the final score was rescaled by dividing by 1000. The total EDII score ranged from negative to positive values, where a more negative score indicates a more anti-inflammatory diet and a more positive score indicates a more proinflammatory diet. A score of close to 0 indicates an inflammatory-neutral diet Modifications to index: Tabung 2017 [29] included an additional proinflammatory component, Organ meat	(-ve to +ve values)	Weights (β coefficient) based on their strength of association with inflammation
FDII Salari-Moghaddam 2021 [28]	Mirrafiei 2023 [57]	28	Anti-inflammatory foods: fruits, fruit juices, fish, poultry, cruciferous vegetables, yellow vegetables, green leafy vegetables, other vegetables, tomatoes, legumes, whole grains, tea. Proinflammatory foods: processed meats, red meats, eggs, butter, dairy, coffee, potatoes, French fries, refined grains, pizza, snacks, mayonnaise, soft drinks, sweets/desserts, hydrogenated fats, hydrogenated oils.	Consumption of dietary components was adjusted for total energy using the residual method. The overall score was obtained by summing the weighted number of daily serves, and the final score was rescaled by dividing by 100 to reduce the magnitude of the score. A higher score indicated a more proinflammatory diet	(-ve to +ve values)	Weights (β coefficient) based on their strength of association with inflammation
IFI Riboldi 2022 [58]	N/A	18	Anti-inflammatory foods: fruits, nuts, whole-grain cereal, chicken, butter, pizza, wine. Proinflammatory foods: seafood, pork, red meat, processed meat, hot dogs, artificial juice (added sugar), artificial juice (sugar-free), coffee (added sugar), soda (sugar-free), soda (added sugar), beer.	IFI was categorized into tertiles where the highest quintile (Q3) indicated a more proinflammatory diet and lower Quintile (Q1) a more anti-inflammatory diet. IFI was calculated for each participant by multiplying intake of each dietary component by its weighting (β-coefficient) and then summing the weighted component to derive an overall IFI score. • Anti-inflammatory foods: each dietary component was assigned a negative weighting • Proinflammatory foods: each dietary component was assigned a positive weighting A positive overall score indicated a more proinflammatory diet	(-ve to +ve values)	Weights (β coefficient) based on their strength of association with inflammation
PAIFIS Azevedo-Garcia 2023 [37]	N/A	7	Anti-inflammatory foods: fruits, vegetables. Proinflammatory foods: red meat, processed meat, candies, snacks, SSB.	Daily consumption of each pro-and anti-inflammatory dietary component was calculated (g/d or mL/d). Sum of total proinflammatory food intake was subtracted from sum of total anti-inflammatory food intake to derive the overall PAIFIS. A higher PAIFIS indicates a more proinflammatory diet	(-ve to +ve values)	No cut-offs or weights. Literature derived pro- and anti-inflammatory groups

Indexes based on dietary guidelines recommendations						

Alternative to Healthy Eating Index/ Healthy Eating Index						
AHEI McCullough 2002 [59,60]	Akbaraly 2015 [61]; Fargnoli 2008 [62]; Fung 2005 [42]; Piccand 2019 [63]; Vahid 2023 [64]	9	Fruits, vegetables, nuts/soy, cereal fiber, white: red meat ratio, alcohol (nutrients: PUFA:SFA ratio, trans-fat, multivitamins).	Based on Dietary Guidelines for Americans and Food Guide Pyramid. • Each dietary component (except Multivitamins, Alcohol) was weighted on a 10-point scale, with 0 points (lowest adherence) to 10 points (maximal adherence). Intermediate intake was proportionally scored • Multivitamins: dichotomous score: 2.5 points (<5 y) and 7.5 points (≥ 5 y) • Alcohol scores were based on U-shaped values, with highest score assigned for moderate intake or proportional score of 0–10 points[42] Overall score: A higher score indicates greater adherence to dietary guidelines Modifications to index: Fung 2005 [42]: intermediate intake was scored as 1 point deducted for each 10% percent decrease in consumption. Piccand 2019 [63]: (i) trans-fats were omitted from the score due to insufficient data; (ii) Multivitamins: dichotomous score: 7.5 points (any intake), otherwise 0 points Vahid 2023 [64]: (i) trans-fats were omitted from the score due to insufficient data; (ii) Multivitamin intake was dichotomous: 10 points assigned for consuming any supplement, otherwise 0 points.	(2.5–87.5) (0–77.5) [65] (0–75) [66]	Cut-off values based on dietary guidelines
AHEI-2010 Chiuve 2012 [67]	Huang 2016 [43]; Ko 2016 [68]; Li 2021 [69]; Li 2023 [70]; Mattei 2017 [69]; Mears 2019 [45]	11	Fruit, vegetables, nuts/legumes, whole grains, red/processed meat, SSB, alcohol (nutrients: omega-3 FAs, PUFAs, trans-fats, sodium).	Based on Dietary Guidelines for Americans and Food Guide Pyramid. • Each dietary component (except alcohol) was weighted on a 10-point scale. (0 points for lowest adherence to 10 points for maximal adherence). Intermediate intake was proportionally scored. • Alcohol scores were based on U-shaped values, with highest score assigned for moderate intake Overall score: A higher score indicates greater adherence to dietary guidelines. Modifications to index: Li 2023 [70]: (i) trans-fat was omitted due to insufficient data; (ii) alcohol was omitted from the score; (iii) vegetables excl. potatoes.	(0–110) (0–100) [69] (0–90) [70]	Cut-off values based on dietary guidelines
HEI Kennedy 1995 [71]	Ford 2005 [72]; Fung 2005 [42]; Kant 2013 [73]	10	Fruits, vegetables, grains, dairy, meats, dietary variety (nutrients: SFA, total fat, cholesterol, sodium).	• Each dietary component was assigned a score from 0 points (lowest adherence) to 10 points (maximal adherence). Intermediate intake was proportionally scored. Overall score: A higher score indicates greater adherence to dietary guidelines Modifications to index: Kant 2013 [73]: Diet variety component was adapted to consist of all unique foods within Fruit, Vegetables, Meat/alternatives, Grains, Dairy groups. Mixed dishes contributed to score for each dietary component food group.	(0–100)	Cut-off values based on dietary guidelines
HEI-2010 Guenther 2014 [65]	Mattei 2017 [74]; Monfort-Pires 2014 [66]	12	Total fruit, whole fruit, total vegetables, dark green/orange vegetables/legumes, total grains, whole grains, dairy, meat/beans, oils (vegetable/fish/nuts/seeds), (nutrients: SFA, SFAAS, sodium).	Each component scored using an energy density approach (consumption per 1000 kcal) • Fruit, Vegetable, and Grain components (n = 5) were weighted on a 5-point scale. (0 points for lowest adherence to 5 points for maximal adherence). • Solid fats and alcohol/added sugars (%E) were weighted on a 20-point scale. (0 points for lowest adherence and 20 points for maximal adherence). • All other components were weighted on a 10-point scale. (0 points for lowest adherence and 10 points for maximal adherence). Intermediate intake was proportionally scored. Overall score: a higher score indicates greater adherence to dietary guidelines	(0–100)	Cut-off values based on dietary guidelines
HEI-2015 Krebs-Smith 2018 [75]	Bérard 2020 [76]; Hayuningtyas 2021 [39]; Li 2021 [77]; Li 2023 [70]; Matsunaga 2021 [78]; Millar 2021 [79,80]; Vahid 2022 [81]; Wang 2023 [82]	13	Adequacy components: total fruit, whole fruit, total vegetables, greens/beans, whole grains, total protein foods, seafood/plant proteins, dairy, (nutrients: PUFA:SFA ratio). Moderation components: refined grains and foods with added sugars, (nutrients: SFA, sodium).	Based on Dietary Guidelines for Americans (2015). Scores for each component (excl. Fatty acids) based on energy density per 1000 kcal. • Specific adequacy components (Whole grains, Dairy, PUFA:SFA ratio) were weighted on a 10-point scale. (0 points for lowest adherence and 10 points for maximal adherence). • All other adequacy and moderation components were weighted on a 10-point scale. (0 s for lowest adherence to 10 points for maximal adherence). Overall score: A higher score indicates greater adherence to dietary guidelines. Diet quality categories: Good quality: >80 point; Improvement needed: 51–80 points; Low quality: <51 points	(0–100)	Cut-off values based on dietary guidelines
HEIFA Roy 2016 [83]	English 2023 [84]	10	Core food groups (fruits, vegetables, grain foods, meat/protein alternatives, milk/ alternatives), foods with added sugar, water, alcohol (nutrients: SFA, sodium).	• All components (except Water and Alcohol) were weighted on a 10-point scale. (0 points for lowest adherence and 10 points for maximal adherence) • Water was weighted on a 5-point scale • Alcohol was weighted on a dichotomous score (0 or 5 points)	(0–100)	Cut-off values based on Australian Guide to Healthy Eating
Diet Quality						
DDS-R Kant 2004 [85]	Kant 2013 [73]	5	Fruit, vegetables, grains, dairy, meat.	• Consumption ≥ predefined minimum threshold was assigned 1 point for any recommended food. The mixed dishes that met the recommended criteria were assigned 1 point to the corresponding food group. Intake for each food group contributed 1 point to the overall score. A higher overall score indicates higher adherence to recommendations Modifications to index: Kant 2013 [73]: original DDS-R.[85] was adapted in the current study to include only foods from each of the 5 food groups currently recommended in dietary guidelines	(0–5)	Cut-off values based on dietary recommendations
DHD-2015 Looman 2017 [86]	de Graaf 2022 [87]	13	Fruit, vegetables, whole: refined grain ratio, legumes, nuts, dairy, fish, red meat, processed meat, tea, fats/oils, SSB/fruit juice, alcohol.	Based on Dutch Dietary Guidelines. • Consumption for each component was defined as minimum, maximum, or optimum in accordance with dietary guideline recommendations. • Based on these 3 criteria, each component was weighted on a 10-point scale. (0 points for lowest adherence and 10 points for maximal adherence). Intermediate intake was proportionally scored. Higher overall score indicated higher adherence to guidelines Modifications to index: de Graaf 2022 [87]: due to insufficient data, filtered and unfiltered coffee could not be differentiated, and salt intake could not be calculated. Therefore, 13 components were included instead of 15 in the original score.	(0–130)	Cut-off values based on Dutch Dietary Guidelines
DQI Kim 2003 [88]	Alkerwi 2015 [19]; Chan 2019 [89]; Vahid 2023 [64]	16	Variety components: fruits, vegetables, grains, meat/poultry/fish/egg, dairy/beans. adequacy components: fruits, vegetables, grains, protein foods. (Nutrients: fiber, iron, calcium, vitamin C). Moderation components: Total fat. (nutrients: SFA, cholesterol, sodium, empty calories) Overall balance: (nutrients: macronutrient ratio, fatty acid composition).	Based on Dietary Guidelines and Food Guide Pyramid. Scoring of 4 major factors: • Variety components (maximal score 20 points): (i) each component is assigned 3 points (≥1 serve/d), otherwise 0 points. Maximum score (15 points) for consumption of >1 serving/d from each food group; (ii) Within-group variety for protein source: 5 points (≥3 different sources), otherwise 0 points. • Adequacy components (maximal score 40 points): Each component was weighted on a 5-point scale. (0 points for lowest adherence and 5 points for 100% adherence). • Moderation components (maximal score 30 points): Each component scored 0 points (highest intake), 3 points (medium intake), or 6 points (lowest intake). • Overall balance components (maximal score 10 points): Macronutrient ratio was weighted on a 6-point scale; Fatty acid composition was weighted on a 4-point scale.	(0–100) (0–94) [62]	Cut-off values derived from dietary guidelines, Food Pyramid, and other dietary indexes
DQI-SNR Drake 2011 [25]	Dias 2015 [90]	6	Fruit/vegetables, dietary fiber, fish/shellfish, foods with added sugar (nutrients: SFA, PUFA).	Based on Swedish Dietary Guidelines and Swedish Nutrition Recommendations 2005. Each component contributed 1 point for adherence to recommendations, otherwise 0 points. Adherence categories: High (4–6 points); Medium (2–3 points); Low (0–1 points) Modifications to index: Chan 2019 [89]: due to insufficient data to calculate empty calories, the moderation component had a maximum score of 24 instead of 30, reducing the overall total score. Dias 2015 [90]: scoring cut-offs for SFA, fiber, and fruit/vegetables were modified due to small percentage of participants that reached recommendations	(0–6)	Cut-off values based on Swedish Dietary Guidelines and Swedish Nutrition Recommendations 2005
DQS Toft 2007 [91]	Rostgaard-Hansen 2023 [92]	4	Fruit, vegetables, fish, fats.	Based on Danish Dietary Guidelines • Each component was weighted on a 2-point scale. (0 points for lowest adherence to 2 points for maximal adherence). Intermediate intake was proportionally scored • Fats component: points assigned for using only SFA for spreads/cooking (0 points); using vegetable oil/margarine only (1 point); no use of spread/fat except olive oil for cooking (2 points). A higher score indicates greater adherence to dietary guidelines. Overall score was classified into 1 of 3 categories: unhealthy dietary habits, average dietary habits, healthy dietary habits	(0–8)	Cut-off values based on Danish Dietary Guidelines
RCI Alkerwi 2012 [93]	Alkerwi 2015 [19]	13	Fruits/vegetables, grains products, total fiber, sea products, dairy products, meat/poultry/fish/ eggs, total protein, nonalcoholic beverages (nutrients: total carbohydrate, total fat, SFA, simple sugar, sodium).	Based on Luxembourg National Dietary Guidelines Scoring was based on U-shaped values • Each component (except sodium and fruit/vegetable) scored from 0 points (lowest adherence), 0.5 points (inadequate or excessive) intake, and 1 point (maximal adherence) • Sodium component: assigned reverse scores: -0.5 points (excessive salt intake) increasing by 0.5point up to 1 point (minimal intake) • Fruit/vegetables component assigned 2 points (daily intake) to 0 points (minimal intake) Higher overall score indicated higher adherence to guidelines	(–0.5 to 14)	Cut-off values based on Luxembourg National Dietary Guidelines
RFS Kant 2000 [94]	Kant 2013 [73]	6	Fruits incl. juices, vegetables excl. fried/pickled/creamed, whole grains, lean meats/ poultry/fish/alternatives, low-fat mixed dishes.	• Each component scored 1 point (minimum threshold met), otherwise 0 points Intake of a recommended food contributed only 1 point to the score regardless of being reported more than once. A higher overall score indicated higher adherence to recommendations. Modifications to index: Kant 2013 [73]: the original RFS [94] was computed using FFQ data from the Breast Cancer Detection and Demonstration Project cohort. For the purposes of the current study, the RFS was adapted for 24-h recall data.	(0–6)	Cut-off values based on Dietary Guidelines for Americans
RFS McCullough 2002 [59]	Fung 2005 [42]	5	Fruits incl. juices, vegetables incl. juices, grains, dairy, proteins.	• Each component within each food group was scored 1 point (consumed ≥once/wk); otherwise—0 points. A higher overall score indicated higher adherence to guidelines	(0–51)	Cut-off values based on Dietary Guidelines for Americans

INDEXES BASED ON THERAPEUTIC DIETS						

AHA-DS Mattei 2013 [95]	Mattei 2017 [71]	11	Fruit, fruit/vegetable variety, whole grains, fish, foods with added sugars, alcohol (nutrients: total fat, SFA, trans-fats, cholesterol, sodium).	Scores for each component were based on adherence to recommendations or sex-specific tertile in the absence of a cut-off. Scores for each component ranged from 0 (minimal) to 4, 6, or 10 points (maximum adherence), with intermediate values prorated. A higher overall total score indicates greater adherence to AHA recommendations	(0–90)	Cut-off values based on recommendations for CVD risk reduction values (or sex-specific tertile in the absence of a cut-off value)
DASH-S Fung 2008 [96]	Alkerwi 2015 [19]; Ko 2016 [68]; Li 2023 [70]; Mattei 2017[74]; Millar 2021 [79, 80]; Nilsson 2019 [46]; Vahid 2023 [64]; van der Pligt 2024 [50]; Weber 2024 [97]	8	Fruit, vegetables, nuts/legumes, whole grains, low-fat dairy, red/processed meat, SSB (nutrients: sodium).	Population-based quintile: • Consumption from each component was weighted on a 5-point scale, with points for lowest adherence and 5 points for maximal adherence). Intermediate intake was proportionally scored. Total DASH scores were categorized into quintiles (Q1–Q5), where Q5 indicates greater adherence to DASH recommendations	(8–40)	Cut-off values based on recommendations for CVD risk reduction
DASH-S Gunther 2009 [98]	English 2023 [84]	8	Fruits incl. juice, vegetables, grains, dairy, meat/poultry/ fish/eggs, nuts/seeds/legumes, fats/oils, sweets.	Each participant was assigned an energy level based on age, sex, and PA level. Each dietary component was then standardized to the assigned energy level, and lower intakes were scored proportionally. All components (except Grains and Dairy) were weighted on a 10-point scale. (0 points for lowest adherence and 10 points for maximal adherence) Grains and dairy were each divided into 2 sub-groups, which were weighted on a 5-point scale. (0 points for lowest adherence and 5 points for maximal adherence) Dairy: total dairy (0–5 points) and low-fat dairy (0–5 points) Grains: whole grains (0–5 points), high-fiber grains (0–5 points)	(0–80)	Cut-off values based on recommendations for CVD risk reduction
DASH-S Matsunaga 2018 [99]	Matsunaga 2021 [78]	9	Adequacy components: Fruits, vegetables, whole grains, plant protein, dairy products. Moderation components: Animal proteins, foods with added sugars (nutrients: SFA, sodium).	• Each component was weighted on a 10-point scale. (0 points for lowest adherence and 10 points for maximal adherence) Total DASH scores were categorized into quintiles (Q1–Q5).	(0–90)	Cut-off values based on recommendations for CVD risk reduction
MIND-S Morris 2015 [100]	Chan 2019 [89]	9	Berries, green leafy vegetables, other vegetables, nuts, whole grains, wine, animal fat, cheese, pastries/sweets.	• Each component (except olive oil) was assigned a score of 0 or 1 according to intake (frequency and portion). • Olive oil, was assigned 1 point if used as primary oil, otherwise 0 points. Overall, a higher score indicates higher adherence to MIND Modifications to index: Chan 2019 [89]: due to lack of data, olive oil, fish, beans, poultry, red/processed meat, fried/fast foods were omitted from the MIND score	(0–9)	Cut-off values based on scientific evidence for cognitive health

Indexes based on dietary patterns						

Healthy Nordic Dietary Pattern						
BSDS Kanerva 2014 [101]	Kanerva 2014 [102]; Tertsunen 2022 [103]	9	Nordic fruits (apples, pears, berries); Nordic vegetables (tomatoes, cucumber, leafy vegetables, roots, cabbages, peas); Nordic cereals (rye, oat, barley); Low-fat/fat-free milk; Nordic fish (salmon/freshwater fish), red/processed meat; alcohol, (nutrients: total fat, PUFA: SFA ratio).	Cut-off values were based on study- and sex-specific quartiles of average daily intake of each component except alcohol. • Each component (except alcohol) was weighted on a 3-point scale. (0 points for lowest adherence and 3 points for maximal adherence) • Alcohol was assigned 1 point for low intake; otherwise, 0 points A higher score indicated higher adherence to the Baltic Sea diet. Modifications to index: Tertsunen 2022 [103]: due to insufficient data on intake of individual fruits, vegetables, or grains, authors of the current study used broader categories of consumption. In the HNDS, ‘Whole grains’ group replaced the BSDS ‘Rye/oats/barley’ group	(0–25)	BSDS was calculated using the population-based consumption quartiles or medians as cut-offs
Mediterranean Dietary Pattern						
aMED Fung 2005 [42]	Fung 2005 [42]; Li 2023 [70]	9	Fruits, vegetables excl. potatoes, legumes, nuts, whole grains, fish, red/processed meat, alcohol (nutrients: MUFA:SFA ratio).	Fung 2005[42]: Each component (except Red/Processed meat and Alcohol) scored 1 point for intake > median intake, otherwise 0 points. • Red/processed meat was scored 1 point for intake ≤median intake (serve/d), otherwise 0 points. • Alcohol was scored 1 point for moderate intake, otherwise 0 points. Modifications to index: Li 2023 [70]: score was calculated based on the study population’s sex-specific quintiles of food component consumption. Each component was weighted on a 5-point scale. (0 points for lowest adherence and 5 points for maximum adherence) Fung 2005 [42]: original MDS [104] was adapted based on eating behaviors consistently associated with lower risk of chronic disease in clinical and epidemiologic studies as follows: (i) excl. potatoes from vegetable group, (ii) separate fruit and nut intake into 2 groups, (iii) eliminate the dairy group, (iv) incl. only whole-grain products in the grain group, (v) incl. only red/ processed meats in the meat group, (vi) moderate alcohol intake to 5–15 g/d	(0–9) [42] (9–45)[70]	Cut-off values (or median intake in the absence of a cut-off value) based on adherence to Mediterranean dietary pattern
MDS Martínez-González 2002 [105]	Serrano-Martinez 2005 [106]	8	Fruit, vegetables, fiber, fish/seafood, olive oil, alcohol, meat/processed meat, carbohydrate-rich foods..	Average daily consumption was adjusted for total energy intake. Each component was weighted on a 5-point scale, and overall score was obtained by summing the quintile values. • Beneficial components that align with the Mediterranean diet were positively scored from 5 points (highest intake) to 1 point (lowest intake). • Detrimental components not aligned with the Mediterranean diet were assigned reverse scores from 1 point (highest intake) to 5 points (lowest intake).	(8–40)	Cut-off values (or median intake in the absence of a cut-off value) based on adherence to Mediterranean dietary pattern
MDS Stewart 2016 [107]	Waldeyer 2018 [108]	7	Fruits, vegetables, nuts/legumes, whole grains, fish, meat, alcohol.	Each component was weighted on a 4-point scale, and the overall score was obtained by summing the quintile values. • Beneficial components that align with the Mediterranean diet were positively scored from 4 points (highest intake) to 0 points (lowest intake). • Detrimental components not aligned with the Mediterranean diet were assigned reverse scores from 0 points (highest intake) to 4 points (lowest intake). • Alcohol scores were based on U-shaped values, with highest score assigned for moderate intake.	(0–28)	Cut-off values (or median intake in the absence of a cut-off value) based on adherence to Mediterranean dietary pattern
MDS Trichopoulou 2005 [104, 109]	Alkerwi 2015 [19]; Arouca 2020 [38]; Bonaccio 2023 [110]; Chan 2019 [89] Dai 2008 [53]; Li 2021 [69]; Mattei 2017 [74]; Millar 2021 [79, 80]; Moradi 2020 [111]; Piccand 2019 [63]; Vahid 2023 [64]; van der Pligt 2024 [50]; Vicente 2023 [112]; Weber 2024 [97]	9	Fruits/nuts, vegetables, legumes, cereal, fish, dairy, meat (red meat and poultry), alcohol (nutrients: MUFA:SFA ratio).	Sex-specific medians were used as the cut-off values and adjusted for total energy. Each component (except alcohol, dairy, and meat) was scored 1 point for intake ≥ median intake, otherwise 0 points • Detrimental components not aligned with the Mediterranean diet were assigned reverse scores with 1 point (<median intake), otherwise 0 points. • Alcohol scores were based on U-shaped values, with 1 point assigned for moderate intake Modifications to index: Dai 2008 [53]: The score was constructed using twin zygosity-specific, rather than gender-specific, median of food intake (adjusted to 2500 kcal). Li 2021[69]: due to the consistent association between red/processed meats with cardiometabolic conditions and cancer, and lack of association for poultry, only red/processed meats were included, and assigned reverse scores in the current study. Moradi 2020 [111]: the original MDS was adapted for implementation and uptake in non-Mediterranean countries and in consideration of Irish Dietary Guidelines: (i) Alcohol was omitted from the score, (ii) Nuts/ legumes component was separated into 2 individual components Vicente 2023 [112]: only foods considered authentic to the traditional Mediterranean diet (fresh, locally produced, no any minimal modification from their natural state) was included in the score. Piccand 2019 [63]: authors adapted the MDS [104] to the Swiss population, whereby dairy was considered healthy. van der Pligt 2024 [50]): based on dietary recommendations for pregnancy (i) Alcohol component was omitted from the MDS, (ii) low-fat dairy was omitted from the Dairy group (detrimental component)	(0–9) (0–8) [50]	Cut-off values (or median intake in the absence of a cut-off value) based on adherence to Mediterranean dietary pattern
MDS Trichopoulou 2003 [104]	Piccirillo 2022 [113]	9	Fruit, vegetables, combined fruit/vegetable, legumes, wholegrains, olive oil, fish, meat, wine.	Each component was assigned 1 point for meeting recommendations, otherwise 0 points The total MDS was divided into 3 adherence categories: low adherence (0–3 points); moderate adherence (4–5 points), high adherence (6–9 points) Modification to index: Piccirillo 2022 [113]: authors used a simplified version of the original MDS [104,113]: authors used a simplified version of the original MDS [104].	(0–9)	Cut-off values (or median intake in the absence of a cut-off value) based on adherence to Mediterranean dietary pattern
MDS Whalen 2014 [114]	Whalen 2016 [115]	11	Fruit, vegetable, nuts, grains/starches, fish, dairy, lean meats, red/processed meat, alcohol (nutrients: MUFA:SFA ratio, sodium).	Each participant was assigned a quintile rank based on sex-specific distribution in study population Each component was weighted on a 5-point scale, and the overall score was obtained by summing the quintile values. • Beneficial components that align with the Mediterranean diet were positively scored from 5 points (highest intake) to 1 point (lowest intake). • Detrimental components not aligned with the Mediterranean diet were assigned reverse scores from 1 point (highest intake) to 5 points (lowest intake). • Moderate alcohol intake was assigned 5 points; otherwise, it was 1 point. Modifications to index: Whalen 2016 [115]: authors adapted the scoring scheme for Dairy, Grains/starches, Alcohol components.	(11–55)	Cut-off values (or median intake in the absence of a cut-off value) based on adherence to Mediterranean dietary pattern
MDS Willett 1995 [116]	Savard 2021 [48]	11	Fruits, vegetables, legumes/nuts/seeds, whole-grain products, fish/seafood, olive oil, poultry, dairy, eggs, red/processed meats, sweets.	Each participant was categorized into quintiles based on overall MDS. Each component was weighted on a 4-point scale and then categorized into quintiles based on this score: Q5 indicated the highest adherence and Q1 for lowest adherence. • Beneficial components that align with the Mediterranean diet were positively scored from 4 points (highest intake) to 0 points (lowest intake). • Detrimental components not aligned with the Mediterranean diet were assigned reverse scores from 0 point (highest intake) to 4 points (lowest intake). • Dairy scores were based on U-shaped values, with 4 points assigned for moderate intake	(0–44)	Cut-off values (or median intake in the absence of a cut-off value) based on adherence to Mediterranean dietary pattern
MEDAS Martínez-González 2012 [117,118]	Bérard 2020 [76]; English 2023 [84]; Lahoz 2018 [119]; Pocovi-Gerardino 2021 [120]; Sabia 2022 [121]; Viscogliosi 2013 [122]	14	Fruit, vegetables, legumes, nuts, fish, olive oil (primary culinary fat), olive oil, soffrito, white:red meat ratio, red/processed meat, animal fat, SSB, commercial pastries, red wine.	• Beneficial component that aligns with the Mediterranean diet each positively scored 1 point (high intake); otherwise, 0 points. • Detrimental components not aligned with the Mediterranean diet were each reverse scored from 1 point (low intake); otherwise, it was 0 points. Modifications to index: Sabia 2022: authors included 4 additional questions on foods associated with pro- or anti-inflammatory potential. Questions on consumption of sugar, whole-grain cereals, orange vegetables/ fruits, coffee were added to the score.	(0–14) (0–18) [121]	Cut-off values (energy-adjusted) based on adherence to Mediterranean dietary pattern
MedDietSscore Panagiotakos 2006 [123]	Carter 2010 [124]; Sood 2022 [125]	11	Fruit, vegetables, potatoes, legumes, non-refined grains/ cereals, fish, dairy (full fat), olive oil, poultry, red/processed meat, alcohol.	Each component was weighted on a 5-point scale, and overall score was obtained by summing the quintile values. • Beneficial components that align with the Mediterranean diet were positively scored from 5 points (highest intake) to 0 points (lowest intake). • Detrimental components not aligned with the Mediterranean diet were assigned reverse scores from 0 point (highest intake) to 5 points (lowest intake). A higher score indicates higher adherence to the Mediterranean diet. Modifications to index: Sood 2022 [125]: the original MDS [123] was adapted to suit an Australian population cohort. Food groups were adapted to be culturally applicable and quantifiable in relation to the AGHE. Due to insufficient data, olive oil and wine were omitted from the score [125].	(0–45)	Cut-off values (or median intake in the absence of a cut-off value) based on adherence to Mediterranean dietary pattern
MEDI-LITE Sofi 2014 (126)	Cervo 2021 [52]	9	Fruits, vegetables, legumes, cereals, fish/seafood, dairy products, meat/processed meat, alcohol (nutrients: MUFA:SFA ratio).	The absolute cut-off points for each food component were derived from epidemiologic studies. Median values for intake were weighted for the number of participants. • Each food component was assigned a score based on a three-tier scoring system: highest category of intake (2 points), middle category of intake (1 point), and lowest category of intake (0 point) • Beneficial component that aligns with the Mediterranean diet were each positively scored 1 point (high intake); otherwise—0 points • Detrimental components not aligned with the Mediterranean diet were each reverse scored from 1 point (low intake); otherwise—0 points.	(0–18)	Cut-off values based on adherence to Mediterranean dietary pattern
rMED score Buckland 2009 [126]	Fernández-Barrés 2019 [127]	8	Fruits/nuts, vegetables, legumes, cereals, fish, olive oil, meat, dairy products.	All food components were measured as g/1000 kcal/d, and values divided into tertiles. Each component was assigned a score from 0, 1, or 2 points, where 2 points indicated maximal adherence (Q3) and 0 points, lowest adherence (Q1). • Beneficial components that align with the Mediterranean diet were positively scored from 2 points (highest intake) to 0 points (lowest intake). • Detrimental components not aligned with the Mediterranean diet were assigned reverse scores from 0 points (highest intake) to 2 points (lowest intake). Modifications to index: Fernández-Barrés 2019 [127]: the index was adapted for pregnant women by removing alcohol component	(0–16)	Cut-off values (or median intake in the absence of a cut-off value) based on adherence to Mediterranean dietary pattern
Okinawan Dietary Pattern						
ODS Willcox 2007 [128]	Chan 2019 [89]	16	Fruit, sweet potatoes, other potatoes, pickled vegetables, other vegetables, legumes, nuts/seeds, wheat/barley/other grains, rice, dairy, fish, meat (incl. poultry), sugars, oils, eggs, flavors & alcohol.	• Each component was assigned 1 point for consumption that met the recommended ratio of energy intake; otherwise, it was 0 points. A higher indicated higher adherence to the Okinawan diet. Modifications to index: Chan 2019 [89]: the original score included a Seaweed component; however, due to insufficient intake data, the seaweed component was omitted from the score.	(0–16)	Cut-off values based on adherence to Okinawan dietary pattern
Paleolithic Dietary Pattern						
Paleo diet score Whalen 2014 [114]	Whalen 2016 [115]	14	Fruit, fruit/vegetable diversity, vegetable, nuts, grains/starches, dairy, fish, lean meat, red/ processed meat, baked goods, SSB, alcohol (nutrients: calcium, sodium).	Each participant was assigned a quintile rank based on sex-specific distribution in study population Each component was weighted on a 5-point scale, and overall score was obtained by summing the quintile values. • Beneficial components that align with the Paleo diet were positively scored from 5 points (highest intake) to 1 point (lowest intake). • Detrimental components not aligned with the Paleo diet were assigned reverse scores from 1 point (highest intake) to 5 points (lowest intake). • Alcohol scores were based on U-shaped values, with highest score assigned for moderate intake Modifications to index: Whalen 2016 [77]: authors created 2 unique variables: (i) fruit/vegetable diversity as the sum of different fruits and vegetables consumed in given period, where higher diversity was considered favorable; (ii) calcium intake was measured independently of dairy by using a statistical method to separate calcium from dairy intake	(14–70)	Cut-off values based on adherence to Palaeolithic dietary pattern
Plant-based Dietary Pattern						
PBDi Kim 2019 [129]	González-Ortiz 2020 [54]	14	Fruit, vegetables, cereal, refined grains, potato, juice, coffee/tea, jam/sweet drinks/desserts, chocolate/sweets/sugar, meat, fish, egg, spreads, dairy.	Consumption of each component (g/d) was weighted on a 5-point scale. • The sum of quintile values across plant food components was assigned positive scores, 1 point for Q1 (lowest intake) to 5 points for Q5 (highest intake). • Animal food intake (g/d) was transformed into quintiles of distribution and the sum of quintile values was assigned reverse scores: 5 points for Q1 to 1 point for Q5. A higher score indicates higher adherence to a plant-based diet	(14–70)	Based on quintiles for highest/lowest consumption of plant foods and animal foods)
PDI Satija 2016 [130]	Aljuraiban 2022 [40]; Baden 2019 [41]; Huang 2023 [131]; Kharaty 2023 [132]; Pourreza 2021 [47]; Wang 2023 [82]; Weber 2024 [97]	18	Healthy plant foods fruits, whole grains, vegetables, legumes, nuts, vegetable oils, tea/coffee. Less healthy plant foods refined grains, fruit juices, potatoes, SSB, sweets. Animal foods animal fats, meat, dairy, eggs, fish/seafood, miscellaneous animal-based foods.	Scoring components were classified into 3 categories: (i) Healthy plant foods, (ii) Less healthy plant foods, and (iii) Animal foods). Mixed dishes consisting primarily of animal foods were classified as animal foods. • Each component was weighted on a 4-point scale and then categorized into quintiles based on this score: Q5 indicated highest adherence, and Q1 for lowest adherence. • All plant foods (healthy and less healthy) were positively scored from 4 points (highest intake) to 1 point (lowest intake). • Animal foods were assigned reverse scores from 0 points (highest intake) to 4 points (lowest intake). A higher score indicated a higher intake of plant foods and lower intake of animal foods Modifications to index: Aljuraiban 2022 [40]: cut-off values were based on Dietary Guidelines for Saudis	(18–72) (18–90) [41] (18–180) [82]	Based on quintiles for highest/lowest consumption of plant foods and animal foods)
				Baden 2019 [41]; Pourreza 2021 [47]; Weber 2024 [97]. Each component was weighted on a 5-point scale and then categorized into quintiles based on this score: Q5 indicated highest adherence to Q1 for lowest adherence.		
				Wang 2023 [82] Each component was weighted on a 10-point scale where 10 points were assigned for highest decile and 1 point for <lowest decile.		
hPDI Satija 2016 [130]	Aljuraiban 2022 [40]; Baden 2019 [41]; Huang 2023 [131]; Kharaty 2023 [132]; Pourreza 2021 [47]; Wang 2023 [82]; Weber 2024 [97]	18	Healthy plant foods fruits, whole grains, vegetables, legumes, nuts, vegetable oils, tea/coffee. Less healthy plant foods refined grains, fruit juices, potatoes, SSB, sweets. Animal foods animal fats, meat, dairy, egg, fish/seafood, miscellaneous animal-based foods.	As per PDI, with the distinction in scoring of the 2 plant food categories. Scoring components were classified into 3 categories: (i) Healthy plant foods, (ii) Less healthy plant foods, and (iii) Animal foods). Mixed dishes consisting primarily of animal foods were classified as animal foods. • Each component was weighted on a 4-point scale and then categorized into quintiles based on this score: Q5 indicated highest adherence, and Q1 for lowest adherence. • Healthy plant foods were positively scored from 4 points (highest intake) to 1 point (lowest intake). • Less healthy plant foods were assigned reverse scores from 0 points (highest intake) to 4 points (lowest intake). • Animal foods were assigned reverse scores from 0 points (highest intake) to 4 points (lowest intake). A higher score indicated a higher intake of plant foods and a lower intake of animal foods. Modifications to index: Aljuraiban 2022 [40]: cut-off values were based on Dietary Guidelines for Saudis Baden 2019 [41], Pourreza 2021 [47], and Weber 2024 [97]. Each component was weighted on a 5-point scale and then categorized into quintiles based on this score: Q5 indicated the highest adherence to Q1 for lowest adherence. Wang 2023 [82] Each component was weighted on a 10-point scale where 10 points were assigned for >highest decile and 1 point for <lowest decile.	(18–72) (18–90) [41] (18–180) [82]	Based on quintiles for highest/lowest consumption of plant foods and animal foods)
PVDI Martínez-González 2014 [133]	Wang 2023 [82]	12	Plant-based foods fruit, vegetables, potatoes, legumes, nuts, cereals, olive oil. Animal-based foods animal fats, eggs, fish, dairy, meat/ processed meat.	The consumption of each component was divided into deciles, and each decile was assigned a score of 1–10 points. • Positive scores were assigned to each plant-based component • Reverse scores were assigned to each animal-based component Overall scores were calculated, and a higher score indicated a higher intake of plant foods and lower intake of animal foods.	(12–120)	Based on deciles for highest/lowest consumption of plant foods and animal foods)

Abbreviations: %E, percentage of energy; AGHE, Australian guide to healthy eating; AHA-DS, American Heart Association diet score; AHEI, alternative Healthy Eating Index; AIDI-20, Anti-inflammatory diet index; aMED, alternate Mediterranean diet score; BSDS, Baltic Sea diet score; CVD, cardiovascular disease; DASH-S, dietary approaches to stop hypertension score; DDG, Dutch dietary guidelines; DDS-R, dietary diversity score for recommended foods; DG, dietary guidelines; DHD-2015, Dutch Healthy Diet Index; DIS, Dietary inflammation score; DQI, diet quality index; DQI-SNR, diet quality index Swedish nutrition recommendations; DQS, diet quality score; EDII, Empirical Dietary Inflammatory Index; EDIP, Empirical dietary inflammatory pattern; FA, fatty acids; FDII, food-based dietary inflammatory index; FFQ, food frequency questionnaire; HEI, Healthy Eating Index; HEIFA, Healthy Eating Index for Australians; hPDI, healthy plant-based diet index; IFI, Inflammatory food index; LNDR, Luxembourg national dietary recommendations; MDS, Mediterranean diet score; MEDAS, Mediterranean diet adherence screener; MEDI-LITE: literature-derived Mediterranean diet; MIND-S, Mediterranean-DASH Intervention for Neurodegenerative Delay Diet Score; MUFA, mono-unsaturated fatty acids; ODS, Okinawan diet score; PA, physical activity; PAIFIS, Proinflammatory, anti-inflammatory food intake score; Paleo, paleolithic; PBDi, plant-based diet index; PDI, plant-based diet index; PUFA, polyunsaturated fatty acids; PVDI, provegetarian diet index; Q, quartile; RCI, recommendation compliance index; RFS, recommended food score; rMED, Relative Mediterranean diet score; SFA, saturated fatty acids; SFAAS, saturated fat, alcohol, added sugar; SSB, sugar-sweetened beverage.

The characteristics of the 5 studies describing the original development and validation of dietary inflammatory indexes, all published between 2016 and 2023, are shown in Supplemental Table 3 [37,44,49,55,58]. The data used in the development and internal validation of these dietary inflammatory indexes were obtained from large prospective cohorts or cross-sectional studies. The generalizability and robustness of the indexes were further assessed through comparisons across diverse populations as part of the external validation process [44,49,55] (Supplemental Table 3). The characteristics of 60 studies that assessed the relationship between 38 established diet quality indexes and biomarkers of chronic inflammation, published between 2005 and 2024, are summarized in Supplemental Table 4. Most studies that utilized established dietary indexes also used data that originated from large cross-sectional and prospective cohort studies, except for 2 intervention trials [51,112].

### Inflammatory biomarkers

The majority of studies (*n* = 46; 71%) explicitly included inflammation as a key outcome in their research objectives, and C-reactive protein (CRP), or high-sensitivity CRP, was the most frequently evaluated inflammatory biomarker, assessed in all studies except 7 [39,45,52,56,87,106,112]. More than half of the included studies (*n* = 37; 57%) evaluated the association between dietary index and multiple inflammatory biomarkers and/or an inflammatory biomarker score (Supplemental Tables 3 and 4).

### Dietary assessment method

Various dietary assessment methodologies were used in the included studies. FFQs were commonly used (*n* = 44), mostly prevalidated (*n* = 37; 84%), typically administered once (*n* = 38), and where specified, captured dietary intake over the preceding 12 mo (*n* = 12) or 1 to 3 mo (*n* = 3). There was broad variation in the number of dietary items included in the FFQs, ranging from 23 to 190 items (Supplemental Tables 3 and 4). Additionally, several studies (*n* = 16) utilized the 24-h recall method, with some administering 2 (*n* = 7) or 3 (*n* = 5) recalls. Less commonly, studies utilized food diaries ranging from 3 to 7 d (*n* = 4), the Mediterranean diet adherence screener (MEDAS) questionnaire (*n* = 3), and diet history (*n* = 1).

### Summary of evaluated food-based indexes

**FIGURE 2:**
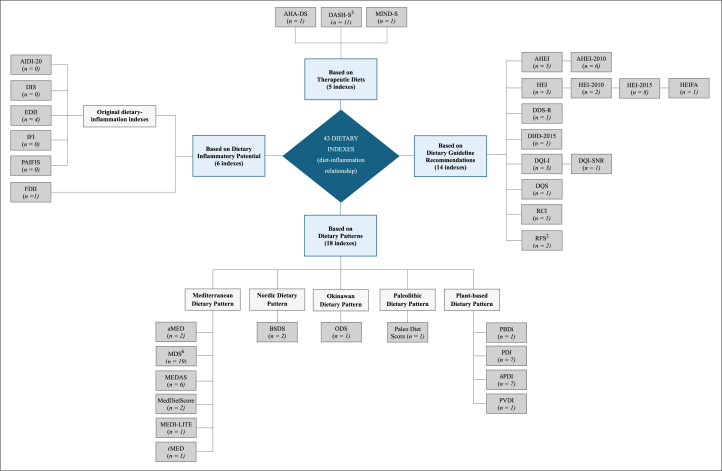
Categorization of 43 dietary indexes evaluated in inflammation-related studies (*n* = 65). The hierarchical structure comprises 4 main categories (light blue), subcategories (light gray), and individual dietary indexes (dark gray). The frequency of each index's evaluation is denoted by “*n*,” representing the number of studies that utilized the index (*n* = 0 indicates that an original dietary inflammatory index has not been further evaluated in eligible studies). Superscript numbers indicate the count of modified or iterated versions of the index included in the review.

The majority of reviewed studies evaluated the relationship between diet and inflammation using a single food-based index. However, several studies compared ≥2 dietary indexes in their evaluations (*n* = 22) [19,[40], [41], [42],^47^,^63^,^64^,[69], [70], [74],^73^,^76^,^78^,^79^,^84^,^84^,^89^,^97^,^111^,^115^,^131^,^132^]. For the purpose of this review, the 43 indexes have been categorized into 4 distinct groups: indexes based on dietary patterns (*n* = 18), indexes based on dietary guideline recommendations (*n* = 14), indexes based on therapeutic diets (*n* = 5), and dietary inflammatory indexes, designed to measure the inflammatory potential of diet (*n* = 6). Figure 2 provides a comprehensive overview of the various dietary indexes evaluated in this review, organized into 4 main categories.

#### Original food-based dietary inflammatory indexes and their association with inflammation

The 5 studies that described the development and validation of original dietary inflammatory indexes included the Anti-Inflammatory Diet Index (AIDI-20) [44], Dietary Inflammation Score (DIS) [55], EDII [49], Inflammatory Food Index (IFI) [58], and Proinflammatory and Anti-inflammatory Food Intake Score (PAIFIS) [37] (Supplemental Table 3). The dietary composition for these indexes was determined using the following methodology: *1*) Spearman’s correlation was used to identify dietary components that exhibited a significant association with inflammatory biomarkers, and only those with statistically significant associations were included in the index [44,55]; *2*) reduced rank regression (RRR) was conducted to derive a dietary pattern associated with inflammatory biomarkers and/or an inflammatory score, followed by stepwise linear regression analysis to identify the dietary components that contributed significantly to the RRR dietary pattern [49,58]; and *3*) dietary components categorized into pro- and anti-inflammatory groups based on the existing literature [37] (Supplemental Table 3). The construct validity of these novel dietary inflammatory indexes was evaluated using multivariable regression models (linear and logistic), specifically, the index’s ability to predict or measure the inflammatory potential of the diet. Adjustments for confounders varied but commonly included age, sex, BMI, energy intake, physical activity, smoking status, and medication use (Supplemental Tables 3).

**FIGURE 3:**
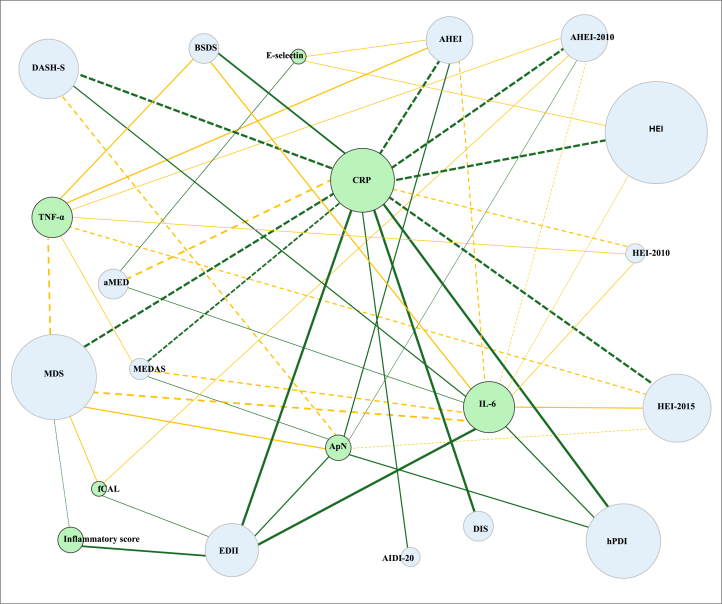
Network map depicting the reported associations between dietary indexes and inflammatory biomarkers: Blue nodes represent a dietary index, and green nodes represent an inflammatory biomarker. Node size indicates assessment extent (larger size represents more studies and larger populations). Lines denote reported associations between the index and the inflammatory marker as follows: Solid green: significant association; Dotted green: mixed findings, majority showing significant association; Solid yellow: no significant association; Dotted yellow: mixed findings, majority showing no significant association; Line thickness represents study/population size: thickest (*n* = ≥10,000), medium (*n* = 5000–9999), thin (*n* = 1000–4999), thinnest (*n* = <1000).

The AIDI-20 demonstrated a significant inverse association with CRP concentrations in Nordic female populations [44]. The DIS and EDII demonstrated a significant positive and linear relationship with proinflammatory biomarker concentrations, with higher inflammatory biomarkers in the highest quintiles [29,49,55]. A higher tertile of the IFI was significantly associated with a more proinflammatory diet and increased odds of developing type 2 diabetes and obesity [58]. Lastly, the PAIFIS exhibited only weak correlations with CRP concentrations [37]. Figure 3 presents a network map summarizing the reported associations between dietary indexes and inflammatory biomarkers across the reviewed studies. In conclusion, the AIDI, DIS, and EDII represent novel and robust food-based indexes for evaluating diet quality in relation to its inflammatory potential (Supplemental Table 3).

#### Established food-based indexes and their association with inflammation

A total of 38 established food-based indexes were used to assess the relationship between dietary intake and biomarkers of chronic inflammation. One-third were updated or adapted versions of an original index (*n* = 14, 36%). Furthermore, several of the included studies implemented their own modifications to the dietary composition and/or scoring of the original index (*n* = 20), predominantly due to limitations in the dietary intake data or to accommodate a specific population cohort [29,42,50,53,63,64,69,70,73,87,89,103,[111], [112], [113], [115],^121^,^125^,^127^]. Typically, studies conducted regression analyses, including logistic, linear, and multivariable models, to assess the association between the index and inflammatory biomarkers.

##### Indexes based on dietary inflammatory potential

Four reviewed studies utilized the EDII to assess the inflammatory potential of diet [29,51,55,56]. The EDII demonstrated the ability to significantly predict circulating concentrations of inflammatory biomarkers in male adults [51]. Higher EDII scores, indicative of a more proinflammatory diet, were inversely associated with adiponectin concentrations [29] and positively associated with proinflammatory biomarkers [29] and fecal calprotectin concentration [59] (Supplementary Table 3). Additionally, the FDII was assessed in a single study with no significant association between the index and inflammation in Iranian adults [57].

##### Indexes based on dietary patterns

Adherence to a Mediterranean dietary pattern was the most extensively assessed, with half (50%, *n* = 30) of the studies employing some version of a Mediterranean-based index. In addition to the 6 different types of Mediterranean-based indexes, several different versions of the Mediterranean diet score (MDS) were also utilized (Table 2) [19,25,28,29,37,[39], [40], [41], [42],[44], [45], [46], [47], [48], [49], [50],^53^,^54^,[55], [56], [57], [58], [61], [59], [60], [62], [63], [64], [68], [67], [69], [70], [74], [72], [71], [73], [65], [66], [76], [75], [77], [78], [79], [80], [81], [82], [84], [83], [85], [87], [86], [88], [89], [90], [91], [92], [93], [94], [95], [96], [97], [98], [99], [100], [102], [101], [103], [104], [106], [105], [108], [107], [109], [110], [111], [112], [113], [115], [114], [116], [119], [117], [118], [120], [121], [122], [124], [125], [123], [134], [127], [126], [128], [129], [130], [131], [132], [133]]. Adherence to the Dietary Guidelines for Americans was also extensively examined, with 45% (*n* = 21) of the included studies utilizing at least 1 version of the Healthy Eating Index (HEI) or the Alternate Healthy Eating Index (AHEI) to assess the association between dietary intake and inflammation (Table 2) [19,25,28,29,37,[39], [40], [41], [42],[44], [45], [46], [47], [48], [49], [50],^53^,^54^,^55^].

The associations between Mediterranean diet-based indexes and inflammation were examined across multiple studies (Figure 3). The MDS score has been extensively examined, with several studies reporting a significant inverse association with CRP concentrations in adult populations [63,69,74,79,80,97,108,113,115,122] and in early pregnancy [50]. Studies have also found significant inverse associations with other proinflammatory biomarkers in adults (53,105) with overweight/obesity [125] and in males and postmenopausal females [124] (Supplemental Table 4). Over time, a higher MDS score was significantly associated with a lower inflammatory score and reduced chronic inflammation [112] in the aging population [110] and older community-dwelling Chinese males but not females [89]. However, several studies have reported no association between MDS and inflammatory biomarkers [48,53,63,64,111]. The MEDAS score was significantly associated with lower CRP concentrations [84,119,120, 121] and higher adiponectin concentrations in childhood acute lymphoblastic leukemia survivors [76]. Higher literature-derived Mediterranean diet scores (MEDI-LITE) were significantly inversely associated with IL-6, although no associations were observed with other inflammatory biomarkers [52]. One study found that a higher alternate Mediterranean diet score was significantly associated with lower concentrations of proinflammatory markers, including CRP, IL-6, and E-selectin in one study [42]. However, another study found no association with CRP concentration [70]. Lastly, the relative Mediterranean diet score showed no association with CRP concentration during pregnancy [127]. Adherence to a healthy Nordic diet, indicated by higher Baltic Sea diet scores, demonstrated a significant inverse association with CRP concentrations [101,103]. Higher Okinawan diet scores were significantly associated with lower CRP concentrations in older community-dwelling Chinese males but not in females [89]. Finally, studies that examined the relationship between inflammation and various indexes based on plant-based DPs found significant positive associations between healthy plant-based diet index scores and adiponectin concentrations [41] and an inverse association between CRP [40,41,47, 82,97,131] and TNF-α [40,41,47, 82,97, 131] concentrations. Similarly, higher plant-based diet index and provegetarian diet index scores were significantly inversely associated with CRP [54,82,84], IL-6 [54], and Lp-PLA_2_ [84] concentrations.

##### Indexes based on dietary guideline recommendations

The associations between inflammatory biomarkers and indexes based on dietary guideline recommendations have been extensively investigated in this review (Figure 3). Several studies have reported significant associations between higher AHEI scores and favorable inflammatory profiles. Specifically, higher AHEI scores were significantly associated with increased adiponectin concentrations [62] and lower concentrations of CRP [43,63,68,69,70], IL-6 [42,45,61], and other proinflammatory biomarkers [42,62,69]. However, some studies found no significant association with proinflammatory biomarkers [63,64,74]. The HEI is an independent negative predictor of inflammation, and several studies have demonstrated significant inverse associations with CRP [70,72,73,66,77,78,81,82] and TNF-α [76] concentrations, as well as a significant positive association with adiponectin concentrations in children [39], although some studies found no associations with proinflammatory biomarkers [74,66,81]. The dietary diversity score revised was an independent negative predictor of CRP [73], and higher diet quality scores [Diet Quality Index International (DQI-I)] were significantly associated with lower CRP concentrations in adults [90,92] and older community-dwelling Chinese males, but not in females [89]. However, one study found no association with CRP [64]. Finally, the recommended food score was an independent negative predictor of CRP and was significantly inversely associated with fibrinogen concentrations [73] (Supplemental Table 4).

##### Indexes based on therapeutic diets

The American Heart Association diet score showed no association with CRP [74]. The dietary approaches to stop hypertension score (DASH-S) demonstrated a significant positive association with adiponectin [46] and an inverse association with CRP [68,70,78,79,80,84,97] in early pregnancy [50] and other proinflammatory biomarkers (TNF-α and IL-6) [79,80]. However, some studies have reported no association between DASH-S and CRP [46, 64,74] (Figure 3). The Mediterranean-DASH Intervention for Neurodegenerative Delay Diet Score (MIND-S) showed that higher scores were significantly associated with lower CRP concentrations in older community-dwelling Chinese males but not in females [89].

### Index scoring structure

The development of original dietary inflammatory indexes commonly utilized multivariable or stepwise linear regression analysis as scoring techniques. Weights (β coefficients) for each dietary component were calculated based on significant associations with inflammation (Supplemental Table 3) [44,49,55,58]. Anti-inflammatory components were assigned negative values, whereas proinflammatory components were assigned positive values. The overall score was then calculated by multiplying the intake of each dietary component by its respective weight and summing the results [49,55,58]. Kaluza et al. [44] employed a different approach, using empirically derived cut-off values and performing regression analyses to identify optimal cut-off thresholds strongly associated with CRP. Finally, Azevedo-Garcia et al. [37] utilized a unique formula to calculate an overall score, derived by subtracting the daily intake of proinflammatory foods from anti-inflammatory foods. In contrast to the other dietary inflammatory indexes, a higher score indicated a more proinflammatory diet (Supplemental Table 3).

Dietary scoring structures varied widely across studies that utilized established indexes (Table 2) [19,25,28,29,37,[39], [40], [41], [42],[44], [45], [46], [47], [48], [49], [50],^53^,^54^,^55^]. Most indexes employed a positive-scoring algorithm, where higher scores indicated greater adherence to dietary guidelines, therapeutic diets (e.g., risk reduction for CVD), or specific dietary patterns. Notably, only 3 studies used factor analysis to derive dietary patterns from the dietary assessment data [68,81,89]. In the absence of a cut-off value, studies typically used population-based and sex-specific consumption quintiles or medians as cut-off values.

### Concurrent validity: intercorrelation analyses of dietary indexes

Several studies (*n* = 22) used >1 dietary index to assess the relationship between dietary intake and inflammatory biomarkers (Supplemental Tables 2 and 3). Of these, 8 studies also conducted intercorrelation analyses to calculate Pearson’s or Spearman’s correlation coefficients, assessing the extent to which the dietary indexes were associated with one another [19,42,64,70,74,73,84] (Supplemental Table 3). Although all dietary indexes analyzed were significantly correlated, the Mediterranean-based dietary indexes (MDS, alternate Mediterranean diet score, and MEDAS), DASH-S, AHEI, and HEI were the most extensively examined and exhibited strong intercorrelations with other indexes.

### Dietary composition and classification in food-based indexes for assessing inflammation

**FIGURE 4:**
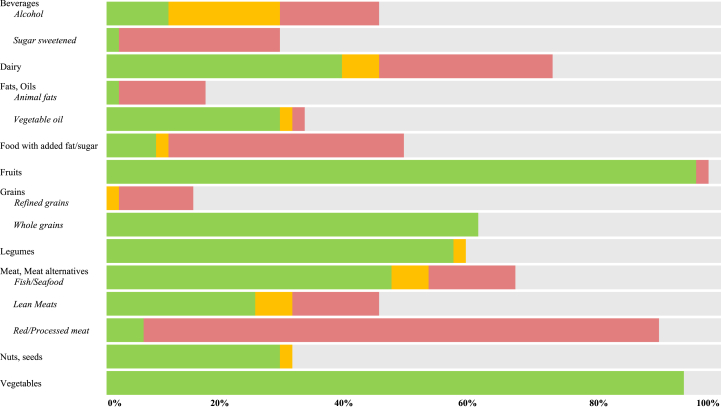
Representation and classification of dietary components in the evaluated indexes. Color coding indicates the following: green: higher intake (≥ recommendations) scored favorably; red: lower intake (≤ recommendations) scored favorably; orange: moderate intake scored favorably; gray: dietary component not included in the index.

All evaluated indexes incorporated food groups, foods, and beverages, and several indexes included ≥1 nutrient in addition to the food and food groups (44%; *n* = 19). However, there was substantial variation in the number and type of dietary components across the 43 indexes (Table 2) [19,25,28,29,37,[39], [40], [41], [42],[44], [45], [46], [47], [48], [49], [50],^53^,^54^,^55^]. Indexes ranged from 4 to 28 dietary components, and several items were noted for their consistent representation across the indexes (differentiated as favorable or unfavorable influences), including fruits, vegetables, grains (especially whole grains), legumes, red/processed meat, dairy, discretionary foods, and alcoholic beverages. Figure 4 summarizes the representation and classification of dietary components across the indexes. A detailed description of this is provided in Supplemental Figure 1.

#### Fruits and vegetables

Fruits and vegetables were the most extensively represented dietary components among the assessed indexes. All indexes incorporated ≥1 fruit and vegetable item, typically as a generalized food group (unspecified). However, some indexes included specific subcategories, such as whole fruits, green leafy vegetables, and potatoes (Supplemental Figure 1). The indexes consistently classified fruits and vegetables as beneficial, assigning higher scores for increased intake, with a few exceptions, such as tomatoes and potatoes, where classification was inconsistent (Supplemental Figure 1).

#### Grains

Almost all indexes (*n* = 39; 90%) incorporated grains, particularly whole grains, as a dietary component (Figure 4). There was consensus across most indexes that grains, except for refined varieties, were considered beneficial dietary components, with higher consumption being favorably scored.

#### Legumes

More than half of the indexes (*n* = 24; 56%) incorporated legumes and consistently classified them as beneficial dietary components (Figure 4). However, their categorization varied across indexes. Whereas typically placed in their own food group, legumes were also grouped with vegetables [65], nuts/seeds [59,67,96,98,107,114], plant proteins [99], and dairy [88].

#### Meat/meat alternative foods

All indexes, with one exception, incorporated ≥1 meat/alternative component. Chan et al. [89] modified the MIND-S to exclude certain dietary components, including meat/alternative foods, owing to insufficient data. Although the indexes exhibited broad diversity in the types of foods included in this food group, most incorporated fish/seafood (*n* = 28) and red/processed meat (*n* = 30). Overall, fish/seafood was considered beneficial, with higher intake favorably scored, and conversely, higher intake of red/processed meat was consistently deemed unfavorable (Figure 4).

#### Dairy

The majority of indexes (*n* = 30; 70%) included dairy as a food group, though the classification of this component varied (Figure 4). Although low-fat dairy was consistently considered beneficial, there was less consensus on the classification of full-fat or unspecified dairy (Supplemental Figure 1).

#### Fats and oils

Olive oil and vegetable oils were consistently represented, particularly in indexes based on Mediterranean and plant-based dietary patterns, with higher intake favorably scored (Figure 4). Animal fats, when included in the indexes, were generally assigned unfavorable scores.

#### Alcohol

Several dietary indexes (*n* = 17) included alcohol, with wine consumption generally receiving favorable scores. Conversely, beer and unspecified alcohol were scored unfavorably or moderately (Supplemental Figure 1).

## Discussion

To our knowledge, this systematic scoping review is the first to synthesize food-based indexes related to dietary inflammation. A total of 43 dietary indexes were identified and categorized according to the following categories: dietary patterns, dietary guidelines, dietary inflammatory potential, and therapeutic diets. Notably, one-third of these indexes were modified to address data limitations or to suit specific populations or cultures.

Notably, this review found robust predictive capabilities of the original AIDI-20, EDII, and DIS in assessing dietary inflammatory potential. Additionally, studies utilizing established dietary indexes showed that Mediterranean-based indexes, namely the MDS, and indexes based on the Dietary Guidelines for Americans [135] were the most extensively utilized. Overall, these dietary indexes demonstrated inverse associations with proinflammatory biomarkers across diverse populations. Additionally, indexes based on plant-based DPs demonstrated significant inverse associations with inflammatory biomarkers, including CRP, IL-6, and TNF-α [136]. These findings align with existing literature, which identifies the Mediterranean dietary pattern as the most extensively studied in nutrition research [[137], [138]]. Previous studies have demonstrated significant inverse associations between both Mediterranean and Vegetarian dietary patterns and chronic inflammation [[137], [138], [139]].

This review examined the methodologies employed to establish the content validity of original dietary inflammatory indexes and found that, overall, studies utilized comprehensive statistical approaches to develop and validate the indexes [37]. For instance, the AIDI and DIS used correlations to assess the relationship between dietary components and inflammatory markers. However, it is worth noting that this approach may oversimplify complex interactions [140] and does not align with the current focus in nutritional epidemiology, which emphasizes dietary patterns over individual nutrients and foods [141]. In contrast, EDII and IFI, developed using RRR, provide more comprehensive analyses of dietary patterns and their relationship with inflammation. These methods offer nuanced insights and improved reproducibility across studies [142,143].

Although these methodological approaches provide valuable insights, the development and application of dietary inflammatory indexes face several challenges inherent to nutrition research, particularly in establishing appropriate cut-off thresholds [[144], [145], [146]]. For instance, the establishment of appropriate cut-off thresholds is a critical consideration in the development and application of dietary indexes, as these thresholds significantly impact the index's effectiveness and interpretability [144,145]. Dietary inflammatory indexes were developed using scores derived from weighted dietary components based on their association with inflammation. Thus, the contribution of each dietary component to the overall inflammation can be quantified [144,145]. Compared with conventional cut-off-based approaches, this method potentially offers a more precise assessment, enhancing the index's discriminative power across diverse populations [145]. However, many of the included indexes used normative cut-off values based on nutritional recommendations. The reliance on a single cut-off point presents inherent limitations [144]. For example, a dietary component consumed below the threshold by the majority of the study population does not contribute to discriminative power and is, therefore, likely to be excluded [144]. This underscores the importance of carefully considering the characteristics of a study population when selecting an appropriate dietary index. As an alternative approach, several studies used population-based and sex-specific medians or quintiles to determine index scores. Although this method offers flexibility and is, therefore, frequently used in nutrition research [145], it is important to note that these thresholds may not align with healthy intake levels, potentially attenuating associations with health outcomes [144,145]. Several indexes, such as DQI-I and HEI, implemented a nuanced scoring system, assigning scores proportionally based on the degree of guideline adherence and potentially mitigating some of the limitations associated with rigid cut-offs [145]. Researchers should carefully consider the limitations of dietary indexes, including the impact of cut-off thresholds, scoring methods, and population-specific factors when selecting, applying, or developing an index for nutrition research. These factors can significantly influence the index's effectiveness, interpretability, and ability to detect associations with health outcomes [144].

Despite methodological challenges in developing and applying dietary indexes, particularly in establishing appropriate cut-off thresholds, significant intercorrelations were observed among food-based indexes. This review examined concurrent validity among the indexes and found that several indexes were significantly inversely associated with inflammation. For example, the AHEI, HEI, MDS, and DASH-S demonstrated strong correlations, reinforcing their consistency in assessing inflammation-related outcomes [147]. The shared dietary composition across indexes enhances the robustness of their association with inflammation [19,71]. This suggests that multiple indexes may reliably assess the diet-inflammation relationship, potentially offering comprehensive and generalizable evaluations across diverse populations and contexts [147,148].

This review identified inconsistencies in the classification of specific dietary components across the evaluated indexes. Namely, fruits, vegetables, whole grains, and legumes were classified as favorable or anti-inflammatory, whereas red and processed meats, foods high in saturated fats and added sugars, and sugar-sweetened beverages were deemed unfavorable or proinflammatory. Interestingly, 2 dietary inflammatory indexes classified discretionary items (pizza and snacks) as anti-inflammatory, although the researchers did not provide a definitive explanation for this unexpected finding [49,58]. Based on the hypothesis that diet can modulate inflammatory processes, anti-inflammatory diets represent a recent strategy for managing and preventing chronic diseases [136,149]. Long-term adherence to dietary patterns emphasizing plant foods, lean proteins, and unsaturated fats is associated with lower inflammation and reduced risk of CVD, morbidity, and mortality [14,136]. Conversely, diets high in red and processed meats, high-fat dairy, refined grains, added sugars, and animal fats are associated with elevated proinflammatory biomarkers and unfavorably affect health outcomes [150,151]. Dietary indexes offer valuable summative measures for quantifying the inflammatory potential of diet. However, they are limited in their ability to elucidate the extent to which specific elements influence health outcomes [152,153]. Furthermore, inconsistencies in the classification and categorization of some dietary components highlight a common challenge in nutrition research [146]. Notably, the categorization of legumes varied substantially across studies, with different indexes grouping them according to specific nutritional attributes. For example, the DQI-I groups beans with dairy due to their calcium content [88]. This was justified based on regions where dairy intake may be lower, and therefore, legumes contribute more significantly to calcium. This diversity in classification underscores the complexity of establishing consistent categories for nutritionally versatile foods such as legumes [154,155] and may reflect disparities in global dietary guidelines [155,156]. Furthermore, few reviewed studies distinguished between healthier and less healthy plant foods such as potatoes, especially when fried. The subtle yet crucial variations in index composition may partially explain the disparate associations observed in reviewed studies (Figure 4) [131]. By differentiating preparation methods in vegetable classification, researchers may provide insights into how different cooking and processing techniques influence the relationship between dietary patterns and inflammatory biomarkers [157,158]. Additionally, incorporating standardized food subcategories will ensure consistency and comparability across studies. By addressing these aspects, researchers can develop more nuanced and accurate tools for assessing the relationship between diet and inflammation across diverse populations and food preparation practices [145,159].

This review revealed that CRP was the predominant biomarker assessed across the reviewed studies. As expected, CRP is the most widely used biomarker for systemic inflammation in clinical practice [4,160]. The lower CRP concentrations observed in this review are potentially clinically meaningful, as these concentrations are associated with a decreased risk of chronic diseases, including CVD, cancer, and all-cause mortality [160,161]. Individuals with elevated CRP concentrations, >3 mg/L, have almost double the risk of developing CVD compared with those with CRP concentrations below 1 mg/L, after accounting for age, ethnicity, and sex [133]. A 10-y follow-up study of older adults without prior CVD found an association between elevated CRP concentration and increased incidence of CVD in both males (33%) and females (17%) [162]. However, it is important to note the broad heterogeneity of the use of other inflammatory markers across studies. Each inflammatory biomarker may reflect slightly different aspects of the inflammatory process [163]. For instance, EDII was significantly inversely associated with fecal calprotectin concentrations, a specific marker of intestinal inflammation, which could be particularly relevant for cohorts with IBD or when investigating diet-related intestinal inflammation [59,164]. Additionally, several studies used a composite inflammatory biomarker score that combines multiple markers to provide a more comprehensive assessment of inflammation. However, the clinical relevance of observed significant changes in these composite scores is unclear, as their interpretation and implications are not well established [11,149,163]. The diversity in biomarker selection highlights the complexity of measuring dietary inflammation. Further studies are required to define optimal biomarkers for assessing dietary inflammation toward a standardized approach to future nutrition research [11,163,165]

Furthermore, the dietary assessment tools used to evaluate nutritional intake are not without limitations that can affect the accuracy and reliability of the data collected [166,167]. FFQs and 24-h recalls, the 2 dietary assessment tools commonly utilized in this review, are limited by potentially random and systematic errors [166,167]. The 24-h recall assesses dietary intake over the previous day in smaller datasets. This methodology is unable to capture day-to-day or within-person variability without complex modeling, limiting its ability to determine usual intake [166]. Multiple 24-h recalls, collected on random, nonconsecutive days, are ideal [167,168]; however, in the current review, few studies administered >2. FFQs are cost-effective methods that reduce within-person variability by estimating usual intake over extended periods but tend to overestimate specific food groups, especially underconsumed foods such as fruits and vegetables [166,167]. Semiquantitative FFQs assess portion sizes and consumption frequency, relying on participants' abilities to accurately recall and estimate intake, potentially leading to low-quality data and misclassification of dietary adherence [168,169]. Despite the need for repeated administrations to assess reproducibility and mitigate errors, few studies utilized multiple FFQs [166,168]. Given these limitations, caution is warranted when using limited dietary data in index development and application, as measurement error and misclassification of dietary adherence may significantly impact the validity and reliability of dietary indexes and their subsequent health-related findings [166,168].

Finally, this review found that over half of the indexes underwent modifications during their application to subsequent studies. Primarily, these modifications were due to insufficient dietary data or population- or cultural-specific requirements. As previously reported, such modifications may introduce inconsistencies and compromise the validity of results, particularly when key dietary components are omitted [144,145]. For instance, Chan et al. [89] modified the MIND-S by excluding several dietary items, leading to the under-representation of the meat/meat alternative food group. This highlights the potential risk of misrepresenting the diet quality when significant modifications are made. Furthermore, indexes are typically designed to evaluate diet quality in relation to a specific health outcome and may require additional validation if applied to different outcomes [144]. Future research should focus on validating modified dietary indexes for specific health outcomes and assessing the impact of excluding or altering food groups on the overall diet quality representation in the context of chronic inflammation.

### Strengths and limitations

The strength of this review lies in its adherence to established methodological [30,31] and reporting [32] guidelines and a systematic, comprehensive search strategy for identifying eligible food-based indexes [170]. However, focusing predominantly on food-based indexes and excluding nutrient-based indexes limits the scope of dietary indexes related to inflammation. Scoping reviews have inherent limitations that must be considered when interpreting findings. Balancing the breadth and depth of analysis is challenging because of the large number of identified articles [30,171]. Specifically, a critical appraisal of the included studies was not conducted as a scoping review focuses on comprehensive coverage over study quality [30,171]. It should also be noted that dietary indexes are inherently related to cuisine, and cultural meaning adaptation will always be required between population groups. Additionally, the data used in the included studies were predominantly derived from cross-sectional studies, which are prone to reporting bias and are unable to establish causal inferences [172]. Finally, the review was limited to studies published in English, potentially missing relevant research in other languages and introducing “English-language bias” [173].

### Implications for future research

In this review, the diverse methodologies and findings were specific to the study populations and inflammatory biomarkers used, necessitating careful consideration in research and clinical applications [16,167]. There is a need for comprehensive validation studies of dietary inflammatory indexes, AIDI-20, DIS, and EDII, across diverse populations and disease states to enhance their robustness and generalizability [26,44]. Furthermore, intervention trials evaluating these dietary inflammatory indexes are necessary to elucidate the anti-inflammatory potential of specific dietary patterns and establish causal relationships between diet and inflammation.

Future research should investigate the use of diverse inflammatory biomarkers and composite inflammatory scores in conjunction with dietary indexes. This approach could serve multiple purposes: *1*) to contribute to establishing the clinical significance and implications of reductions in composite inflammatory biomarker scores [149,163], *2*) to identify optimal biomarkers for assessing dietary inflammation, and *3*) to contribute to developing a standardized methodology for assessing dietary inflammation for future nutrition research [11,163,165]. Such investigations would enhance our understanding of the diet-inflammation relationship across various populations and disease states to inform more targeted nutritional interventions and public health strategies [26,44].

## Conclusion

A comprehensive review of food-based indexes revealed that the AIDI, DIS, and EDII demonstrated robust predictive ability for dietary inflammatory potential. Additionally, established indexes such as AHEI, HEI, and MDS showed significant associations with inflammation across diverse populations and strong intercorrelations. This review highlights methodological challenges to the development and utilization of food-based indexes, emphasizing the need for further nutritional research. Future research should focus on comprehensive validation studies of dietary inflammation indexes across diverse populations, examining diverse inflammatory biomarkers. Researchers should carefully consider the underlying basis of the index, as well as the dietary composition, scoring methods, and the population and health outcomes in which the index has been validated when selecting indexes to assess diet-inflammation relationships. This synthesis contributes to informing future development, validation, and application of inflammation-related dietary indexes in nutritional research and clinical practice.

## Authors contributions

The authors’ responsibilities were as follows – All authors: contributed to the design, methodology, and data extraction for this review; GLR: wrote the first version of the manuscript; all authors: read and critically revised the manuscript; and all authors: reviewed, contributed to, and approved the final version of the manuscript.

## Data availability

All data described and presented in the manuscript will be made available on request.

## Funding

The authors reported no funding received for this study.

## Conflict of interest

The authors have no conflicts of interest to declare.
