# Supplementary material for: Food-based indexes and their association with dietary inflammation
Source: Adv Nutr. 2025 Mar 4;16(4):100400. doi: 10.1016/j.advnut.2025.100400 (PMC11992388; doi:10.1016/j.advnut.2025.100400)
Supplement: Multimedia component 1 [file mmc1.docx]

## Food-based indexes and their association with dietary inflammation.

Gynette L. Reyneke

Online Supplemental Material

**Supplemental Material**

SUPPLEMENTAL TABLES

[**Supplemental Table S1.** Preferred Reporting Items for Systematic reviews and Meta-Analyses extension for Scoping Reviews (PRISMA-ScR) Checklist 2](#_Toc179789768)

[**Supplemental Table S2.** Search strategy for scientific databases 4](#_Toc179789769)

[**Supplemental Table S3.** Summary of characteristics of studies describing the development and validation of novel inflammation-related dietary indexes (food-based indexes based on the inflammatory potential of diet 6](#_Toc179789770)

[**Supplemental Table S4.** Summary of characteristics of studies assessing the association between existing/ established indexes and inflammatory biomarkers. 7](#_Toc179789771)

SUPPLEMENTAL FIGURES

[**Supplemental Figure S1.** Heatmap presenting the representation and classification of dietary components in the included indexes 14](#_Toc179789754)

**Supplemental Table S1.** Preferred Reporting Items for Systematic reviews and Meta-Analyses extension for Scoping Reviews (PRISMA-ScR) Checklist

| **SECTION** | **ITEM** | **PRISMA-ScR CHECKLIST ITEM** | **REPORTED ON PAGE #** |
| --- | --- | --- | --- |
| **TITLE** | | | |
| Title | 1 | Identify the report as a scoping review. | 1 |
| **ABSTRACT** | | | |
| Structured summary | 2 | Provide a structured summary that includes (as applicable): background, objectives, eligibility criteria, sources of evidence, charting methods, results, and conclusions that relate to the review questions and objectives. | 2-3 |
| **INTRODUCTION** | | | |
| Rationale | 3 | Describe the rationale for the review in the context of what is already known. Explain why the review questions/objectives lend themselves to a scoping review approach. | 5-6 |
| Objectives | 4 | Provide an explicit statement of the questions and objectives being addressed with reference to their key elements (e.g., population or participants, concepts, and context) or other relevant key elements used to conceptualize the review questions and/or objectives. | 6-7 |
| **METHODS** | | | |
| Protocol and registration | 5 | Indicate whether a review protocol exists; state if and where it can be accessed (e.g., a Web address); and if available, provide registration information, including the registration number. | 7 |
| Eligibility criteria | 6 | Specify characteristics of the sources of evidence used as eligibility criteria (e.g., years considered, language, and publication status), and provide a rationale. | 7 |
| Information sources* | 7 | Describe all information sources in the search (e.g., databases with dates of coverage and contact with authors to identify additional sources), as well as the date the most recent search was executed. | 8 |
| Search | 8 | Present the full electronic search strategy for at least 1 database, including any limits used, such that it could be repeated. | 8 |
| Selection of sources of evidence† | 9 | State the process for selecting sources of evidence (i.e., screening and eligibility) included in the scoping review. | 8 |
| Data charting process‡ | 10 | Describe the methods of charting data from the included sources of evidence (e.g., calibrated forms or forms that have been tested by the team before their use, and whether data charting was done independently or in duplicate) and any processes for obtaining and confirming data from investigators. | 9 |
| Data items | 11 | List and define all variables for which data were sought and any assumptions and simplifications made. | 9 |
| Critical appraisal of individual sources of evidence§ | 12 | If done, provide a rationale for conducting a critical appraisal of included sources of evidence; describe the methods used and how this information was used in any data synthesis (if appropriate). | N/A |
| Synthesis of results | 13 | Describe the methods of handling and summarizing the data that were charted. | 9 |
| **RESULTS** | | | |
| Selection of sources of evidence | 14 | Give numbers of sources of evidence screened, assessed for eligibility, and included in the review, with reasons for exclusions at each stage, ideally using a flow diagram. | 10 |
| Characteristics of sources of evidence | 15 | For each source of evidence, present characteristics for which data were charted and provide the citations. | 10-11 |
| Critical appraisal within sources of evidence | 16 | If done, present data on critical appraisal of included sources of evidence (see item 12). | N/A |
| Results of individual sources of evidence | 17 | For each included source of evidence, present the relevant data that were charted that relate to the review questions and objectives. | 10-20 |
| Synthesis of results | 18 | Summarize and/or present the charting results as they relate to the review questions and objectives. | 12-19, Table 2 |
| **DISCUSSION** | | | |
| Summary of evidence | 19 | Summarize the main results (including an overview of concepts, themes, and types of evidence available), link to the review questions and objectives, and consider the relevance to key groups. | 20-26 |
| Limitations | 20 | Discuss the limitations of the scoping review process. | 26-27 |
| Conclusions | 21 | Provide a general interpretation of the results with respect to the review questions and objectives, as well as potential implications and/or next steps. | 27-28 |
| **FUNDING** | | | |
| Funding | 22 | Describe sources of funding for the included sources of evidence, as well as sources of funding for the scoping review. Describe the role of the funders of the scoping review. | 28 |

**Supplemental Table S2.** Search strategy for scientific databases

**MEDLINE with Full Text, CINAHL Plus with Full Text**

| SEARCH FIELD | LIMITERS - ENGLISH LANGUAGE; HUMAN SEARCH MODES - BOOLEAN/PHRASE |
| --- | --- |
| Title, Abstract, Subject | "diet* index" OR "diet* indices" OR "diet* quality index" OR "diet* quality indices" OR "eating indices" OR "eating index" OR "diet* score*" OR "anti-inflammat* diet* index" OR "anti-inflammat* diet* indices" OR "anti-inflammat* diet score*" OR "dietary inflammat* index" OR "food score*" OR "food index" OR "food indices"  **AND**  interleukin* OR IL-* OR "c-reactive protein" OR "c reactive protein" OR CRP OR "tumor necrosis factor*" OR "tumour necrosis factor*" OR TNF* OR Adiponectin OR inflammat* OR anti?inflammat* OR cytokine* |
| MeSH Term | cytokines OR tumor necrosis factor-alpha OR c-reactive protein OR interleukins OR inflammation OR inflammation mediators |

**Cochrane CENTRAL**

| SEARCH FIELD | LIMITERS - ENGLISH LANGUAGE; HUMAN SEARCH MODES - BOOLEAN/PHRASE |
| --- | --- |
| Title, Abstract, Subject | "diet index" OR "diet indices" OR "dietary index" OR "dietary indices" OR "diet quality index" OR "diet quality indices" OR "dietary quality index" OR "dietary quality indices" OR "eating indices" OR "eating index" OR "diet score" OR "diet scores" OR "dietary score" OR "dietary scores" OR "anti-inflammatory diet index" OR "anti-inflammatory diet indices" OR "anti-inflammatory dietary index" OR "anti-inflammatory dietary indices" OR "anti-inflammatory diet score" OR "anti-inflammatory diet scores" OR "dietary inflammation index" OR "dietary inflammation indices" OR "food score" OR "food scores" OR "food index" OR "food indices"  **AND**  interleukin OR interleukins OR IL-NEXT* OR "C-reactive Protein" OR "c reactive protein" OR CRP OR "tumor necrosis factor-alpha" OR "tumour necrosis factor" OR TNF OR TNF-a OR adiponectin OR inflammation OR inflammatory OR anti-inflammatory OR anti-inflammation OR cytokine OR cytokines |
| MeSH Term | cytokines OR tumor necrosis factors OR c-reactive protein OR inflammation OR inflammation mediators |

**Pubmed**

| SEARCH FIELD | LIMITERS - ENGLISH LANGUAGE; HUMAN SEARCH MODES - BOOLEAN/PHRASE |
| --- | --- |
| Title, Abstract, Subject | "diet index" OR "diet indices" OR "dietary index" OR "dietary indices" OR "diet quality index" OR "diet quality indices" OR "dietary quality index" OR "dietary quality indices" OR "eating indices" OR "eating index" OR "diet score" OR "diet scores" OR "dietary score" OR "dietary scores" OR "anti-inflammatory diet index" OR "anti-inflammatory diet indices" OR "anti-inflammatory dietary index" OR "anti-inflammatory dietary indices" OR "anti-inflammatory diet score" OR "anti-inflammatory diet scores" OR "dietary inflammation index" OR "dietary inflammation indices" OR "food score" OR "food scores" OR "food index" OR "food indices"  **AND**  interleukin* OR IL-* OR "c-reactive protein" OR "c reactive protein" OR CRP OR "tumor necrosis factor*" OR "tumour necrosis factor*" OR TNF* OR adiponectin OR inflammat* OR anti?inflammat* OR cytokine* |
| MeSH Term | Cytokines OR tumor necrosis factor alpha OR c-reactive protein OR interleukins OR inflammation mediators |

**Embase**

| SEARCH FIELD | LIMITERS - ENGLISH LANGUAGE; HUMAN SEARCH MODES - BOOLEAN/PHRASE |
| --- | --- |
| Title, Abstract, Subject | "diet* index" OR "diet* indices" OR "diet* quality index" OR "diet* quality indices" OR "eating indices" OR "eating index" OR "diet* score*" OR "anti-inflammat* diet* index" OR "anti-inflammat* diet* indices" OR "anti-inflammat* diet score*" OR "dietary inflammat* index" OR "food score*" OR "food index" OR "food indices"  **AND**  interleukin OR interleukins OR IL-NEXT* OR "C-reactive Protein" OR "c reactive protein" OR CRP OR "tumor necrosis factor-alpha" OR "tumour necrosis factor" OR TNF OR TNF-a OR adiponectin OR inflammation OR inflammatory OR anti-inflammatory OR anti-inflammation OR cytokine OR cytokines |
| Keywords | cytokines OR “tumor necrosis factor” OR “c-reactive protein” OR inflammation OR “inflammation mediator” |

**Supplemental Table S3.** Summary of characteristics of studies describing the development and validation of novel inflammation-related dietary indexes (food-based indexes based on the inflammatory potential of diet

| Index | Objective | Study type and origin of data; Participant characteristics | Inflammatory biomarkers | Dietary assessment method | Content validity  (approach to identify food components included in the index) | Scoring (cutoff values/ weighting of index components) | Concurrent validity^a^; Construct validity^b^ (adjusted variables) | Findings, Limitations | Conclusion |
| --- | --- | --- | --- | --- | --- | --- | --- | --- | --- |
| Anti-Inflammatory Diet Index **(AIDI-20)** Kaluza 2018^1^  Sweden  (funding: research council) | Develop a questionnaire-based index and assess its potential to predict chronic low-grade inflammation in a Nordic population. | *Internal validation*: (randomly selected) PCS (Swedish mammography cohort) 1987-1990; (n = 1751); 56-74 y; F; health status NS    *External validation* (generalisability): remaining population; (n = 1751); 56-74 y; F; health status NS | CRP | 123-items FFQ (SA; validated; 12 months)  8 frequency of consumption categories | Spearman’s correlation test used to identify foods correlated with serum CRP concentrations. Only those with statistically significant correlations were included.  Soy, shellfish and wholegrains were excluded due to inadequate intake or measurement across the case-cohort. | Cut-off values based on empirical approach. Different cut-off values were tested using multivariable linear regression models. Components strongly associated with CRP concentrations were included in the index. Scoring of each component was based on the optimal cut-off values.  Higher score indicates a more anti-inflammatory diet. | *Index-inflammation analyses:* Multivariable-adjusted linear regression to assess association between AIDI-20 quintiles and CRP concentrations.  *Adjusted variables:* age, education, energy intake, fat/lean body mass, PA, smoking, menopausal status, medication/HRT use, sleep, chronic disease. | *Index-inflammation association:* Statistically significant inverse association between AIDI-20 and CRP. Females in highest quintile (comparing extreme quintiles) had 26% lower CRP concentrations (95% CI, 18–33%; p <0.001). Each 1 score increment in AIDI-20 was associated with 0.06 (95% CI 0.04–0.08) mg/L lower CRP concentrations.  *Limitations:* Limited to older female population. Assessed a single inflammatory biomarker. | Robust, simple empirically developed index to assess diet quality based on its anti-inflammatory potential.  Future research: validation in diverse populations, utilising other inflammatory biomarkers. |
| Dietary Inflammation Score  **(DIS)** Byrd 2019^2^  USA  (funding: NR) | Develop a novel dietary inflammation score and assess its association with a panel of inflammatory biomarkers in a diverse population. | *Internal validation*:  PCS (REGARDS^3^) 2003 – 2007; (n = 639); M, F; 45-74 y; disease free  *External validation*: 3 validation populations CSS (REGARDS^3^) 2003 – 2007; (MAPs^4,5^) 1994-1997, 2002; (CECP trial^6^) 1990-1991; (n = 14806); M, F; 45-74 y; disease free | CRP and Inflammatory score (CRP, IL-6, IL-8, IL-10) | 109-item FFQ (SA; validated; 12 months)  9 frequency of consumption categories | Spearman’s correlation test used to identify foods correlated with serum CRP concentrations. Only those with statistically significant correlations were included. | Multivariable linear regression analysis was conducted to calculate weights (β coefficient) for each component based on their strength-of-association with inflammatory score. | *Concurrent validity (inter-correlation analysis of dietary indexes):* Spearman’s correlation coefficients were calculated to assess inter-correlations between indexes (DIS, EDIP)  *Index-inflammation analyses:* Multivariable logistic regression models to assess association between DIS and CRP levels and inflammatory scores  *Adjusted variables:* age, comorbidities, energy intake, ethnicity, income, location, medication/HRT use, season, sex. | *Inter-index correlation:* The DIS showed weak to moderate correlations with the EDIP across the validation populations, with Spearman’s correlation coefficients ranging from 0.17 to 0.43. Statistical significance NR  *Index-inflammation association:* DIS was statistically significantly associated with CRP levels in multiple studies. REGARDS REGARDS study: individuals in the highest DIS quintile (comparing extreme quintiles) had 66% (REGARDS) and 2.1-fold (MAP) higher odds (P trend < 0.001) of elevated CRP levels (>3 mg/dL). No significant associations between DIS and inflammatory score.  *Limitations:* DIS weights based on a small US-based sample. Assessed limited inflammatory markers. FFQs are prone to respondent error. | Novel, whole foods-based index to assess the collective contributions of diet on systemic inflammation.  Future research: validation in relation to chronic disease incidence and premature mortality is required. |
| Empirical Dietary Inflammatory Index /Pattern (**EDII/EDIP)** Tabung 2016^7,8^  USA  (funding: govt.) | Develop a food group-based empirical dietary inflammatory index and assess its construct validity in two population cohorts. | *Internal validation*:  PCS (NHS I^9^) 1976; (n = 5230) 30-55 y; F; free of chronic disease  *External validation*:  PCS (NHS II^9^) 1989; (n = 1002) 25-42 y; F; free of chronic disease  (HPFS^10^) 1986; (n = 2632) 40-75 y; M; free of chronic disease | Adiponectin, CRP, IL-6, TNFαR2, Inflammation biomarker score | 2 x 39-item FFQ (validated) | RRR conducted to derive DP associated with inflammatory biomarkers and inflammatory score (RRR DP). Conducted stepwise linear regression analysis to identify food components that significantly contributed to RRR DP  DP). | EDII was calculated by multiplying sum of intake for each food group by its weighting (regression (β) coefficients, derived from stepwise linear regression analysis). These summed values were rescaled (divided by 1000) to provide an overall EDII score.  A negative value indicates an anti-inflammatory food; a positive value indicates a pro- inflammatory food. Scores categorised into EDII quintiles, with the lowest quintile as the reference | *Concurrent validity (inter-correlation analysis of dietary indexes):* Spearman’s correlation coefficients calculated to assess inter-correlations between the EDII and alternative versions.  *Index-inflammation analyses:* Multivariable-adjusted linear regression models assessed associations between EDII and inflammatory biomarkers  *Adjusted variables:* age, BMI, case-control status, co-morbidity score, energy intake, medication/HRT use, menopausal status, PA, smoking. | *Inter-index correlation:* The EDII was significantly strongly correlated with its potential alternative versions.  *Index-inflammation association:* EDII was significantly associated with several inflammatory markers in the development and validation of the index. Individuals in the quintile with the most proinflammatory diets compared to the most anti-inflammatory diets demonstrated 52% higher (NHS-II) and 23% higher (HPFS) CRP levels and more than 3 times higher (NHS-II) and more than 2 times higher (HPFS) Inflammatory Marker Score and 12% (NHS-II) and 13% (HPFS) lower adiponectin levels;  *Limitations*: based on predominantly white study populations. Assessed single measurement of inflammatory markers. Potential bias due to self-reported factors. | Novel empirically derived dietary pattern index to assess diet quality based on its inflammatory potential.  Future research: not reported |
| Inflammatory Food Index (**IFI)** Riboldi 2022^11^  Brazil  (funding: govt.) | Develop an inflammatory food index based on associations between food items and inflammatory biomarkers and to assess its association with weight gain and T2D. | *Internal validation:*  PCS (ELSA-Brasil^12^); 2008-2010; (n = 9,909) 35-74 y; M,F; free of chronic disease | Inflammation biomarker score (CRP, leukocytes) | 114-item FFQ (validated; 12 months) | RRR conducted to derive DP associated with inflammatory biomarkers and inflammatory score (RRR DP). Conducted stepwise linear regression analysis to identify food components that significantly contributed to RRR DP  DP). | IFI was calculated by multiplying sum of intake for each food group by its weighting (regression (β) coefficients, derived from stepwise linear regression analysis)  A negative value indicates an anti-inflammatory food; a positive value indicates a pro- inflammatory food. | *Index-inflammation analyses:* Stepwise linear regression models assessed association with inflammatory score. Logistic regression models assessed association of IFI with major weight gain and Cox regression for association with incident T2D  *Adjusted variables:* behavioural/clinical risk factors, BMI, energy intake, socio-demographic variables. | *Index-inflammation association:* Higher tertiles of IFI were significantly associated with a more pro-inflammatory diet and greater odds of obesity (30%) and developing T2D (26%) .  *Limitations:* FFQ prone to low reliability and measurement error, Assessed only two inflammatory biomarkers. | Findings support the hypothesis that diet-induces subclinical inflammation and suggest that inflammatory potential of diet may play a role in weight regulation and T2D risk.  Future research: intervention trials that further explore the anti-inflammatory potential of various foods. |
| Pro-inflammatory, Anti-inflammatory Food Intake Score **(PAIFIS)** Azevedo-Garcia 2023^13^  South America  (funding: Govt.) | Develop and test the reliability and validity of the PAIFIS in multi-ethnic youth populations of South America | *Internal validation*:  CSS (SAYCARE study^14^); 2013-2015; (n = 661) 3-18 y; M F; health status not specified | CRP | 2 x 59-item FFQ (validated; 3 months)  ≥ 2 x 24-hour recall (by Dietitian) | Foods were categorised according to literature derived pro-inflammatory and anti-inflammatory food groups. | Cut-off values/ weighting not applicable.  Total daily consumption (grams/mL) of pro-inflammatory foods deducted from total daily consumption of anti-inflammatory foods to derive the PAIFIS.  A higher score indicates a more pro-inflammatory dietary. | *Reliability*: Spearman’s correlation to (i) estimate agreement between FFQ (FFQ1 × FFQ2) measurements and (ii)  allow direct comparison between FFQ and 24-hour recall based on categorisation of pro- and anti-inflammatory foods.  *Index-inflammation analyses:* Spearman’s correlation and multilevel linear regression models assessed correlations and associations between PAIFIS and hs-CRP concentrations  *Adjusted variables:*  age, energy intake, location, sex, type of school. | *Index-inflammation association:* PAIFIS demonstrated overall limited ability to predict inflammatory biomarkers. The index explained a moderate amount of variance in CRP levels for children (43.9%, R = 0.48) and adolescents (61.7%, R = 0.33) and demonstrated weak criterion validity for predicting CRP levels in children and adolescents (Spearman’s correlation coefficients ranging from -0.03 to 0.24).  *Limitation:* high attrition and small sample. Assessed only one inflammatory biomarker. | The PAIFIS showed moderate reliability but weak ability to assess the inflammatory potential of diet in children and adolescents.  Further research: future studies should include other inflammatory biomarkers and assess the index in diverse populations. |

^a^ Concurrent validity: the degree to which the index correlates with other established dietary assessment methods or scores.

^b^ Construct validity: the degree to which the index measures inflammatory potential of diet.

Abbreviations: AIDI-20**,** anti-inflammatory diet index; BMI, body mass index; CECP, Calcium and Colorectal Epithelial Cell Proliferation; CRP, C-reactive protein; CSS, cross-sectional study; DIS, dietary inflammation score; DP, dietary pattern; EDII, empirical dietary inflammatory index; EDIP, empirical dietary inflammatory index pattern; F; female; FFQ, food frequency questionnaires; govt, government; HPFS, health professionals follow-up study; HRT, hormone replacement therapy; CRP, high sensitivity C-reactive protein; IFI, inflammatory food index; IL, interleukin; M, male; MAPs, markers of adenomatous polyps; NHS, nurses' health study; NR, not reported; NS, not specified; PA, physical activity; PAIFIS, pro-inflammatory, anti-inflammatory food intake score; PCS, prospective cohort study; REGARDS, reasons for geographic and racial differences in stroke study; RRR, reduced rank regression; SA, self-administered; SES, socio-economic status; SQ, semi-quantitative; T2D, type 2 diabetes mellitus; TNFαR2, tumor necrosis factor alpha receptor-2; y, year; y, years

**Supplemental Table S4.** Summary of characteristics of studies assessing the association between existing/ established indexes and inflammatory biomarkers.

| Study reference | Objective | Study type (origin of data); period | (n), age range; sex; health status | Inflammatory biomarkers | Dietary assessment method | Indexes assessed | Statistical methods for association analysis | Findings |
| --- | --- | --- | --- | --- | --- | --- | --- | --- |
| Akbaraly 2015^15^  Europe  Research institute | Evaluate the impact of long-term adherence to a healthy diet on chronic inflammation over a 5-year follow-up in a large British cohort | CSS (Whitehall II study^16^); 1991/1993; 1997/1999; 2002/2004 | (n = 4600) 35-55 y; M,F; free from acute inflammation | CRP, IL-6 | 127-items FFQ (SQ) | **AHEI** *McCullough et al.^17^* | *Index-inflammation analyses:* Linear regression models  *Adjusted variables:* age, energy intake, ethnicity, living alone, PA, SES, sex, smoking. | *Index-inflammation association:* Higher AHEI scores were associated with significantly lower mean levels of IL-6 (1.84 pg/mL, 95% confidence interval [CI], 1.71-1.98) over the 6-year exposure period. Findings suggest that maintaining/improving adherence to healthy DP may lower risk of chronic inflammation. |
| Aljuraiban 2022^18^  Saudi Arabia  Research institute | To assess the extent to which plant-based indexes correlate with CRP | CSS 2019 | (n = 401) 19-35 y; F; health status NS | CRP | 133-items FFQ (validated: Saudi population) | **hPDI** *Satija et al.^19^*  **PDI** *Satija et al.^19^* | *Index-inflammation analyses:* Multivariate linear regression analyses  *Adjusted variables:* age, BMI, dietary and lifestyle factors | *Index-inflammation association:* Higher hPDI scores were associated with lower CRP levels (0.13mg/L CRP reduction per 6-point increase in hPDI score) (95% CI: 0.08, 0.28) after adjusting for lifestyle and dietary factors. Results were no longer statistically significant after adjusting for BMI. No associations were observed for PDI |
| Alkerwi 2015^20^  USA  Research institute | Assess ability of indexes to detect changes in biomarkers of chronic disease risk | CSS (ORISCAV-LUX^21^); 2007-2008 | (n = 1352) 18-69 y; M,F; health status NS | CRP | 134-item FFQ (SQ; validated) | **DASH-S** *Fung et al^22^*  **DQI-I** *Kim et al.^23^*  **MDS** *Trichopoulou et al.^24^*  **RCI** *Alkerwi et al.^25^* | *Inter-correlation analysis of dietary indexes:* bivariate Spearman’s correlations were calculated to assess inter-correlations between indexes.  *Index-inflammation analyses:* Multiple linear regression analyses  *Adjusted variables:* age, education, energy intake, smoking status, PA, sex. | *Inter-index correlation:* All dietary indexes were significantly correlated with one another, with Spearman’s correlation coefficients ranging from 0.31 (medium) to 0.65 (strong) (all P <0·0001).  *Index-inflammation association:* No significant associations between any diet index and CRP levels. MDS demonstrated the best ability to detect changes in numerous biomarkers of risk in an adult population. |
| Aroke 2020^26^  USA  Research institute | Assess /validate association between dietary indexes with circulating biomarkers and risk of prostate cancer, in PLCO cancer cohort. | RCT (PLCO^27^); 1993-2001 | (n = 3517) 55-74 y; M; free from prostate cancer at enrolment | Adiponectin, CRP, IL-6, TNFα-R2 | 124-item FFQ, 137-item FFQ (SQ; validated: USA; 12 months) | **EDIP** *Tabung et al.^7^* | *Index-inflammation analyses:* Multivariable adjusted linear regression analyses  *Adjusted variables:* age, BMI, education, energy intake, ethnicity, family x of cancer, marital status, medication use, pa, sex, smoking status, study centre. | *Index-inflammation association:* The EDIP significantly predicted circulating concentrations of inflammatory markers in male adults. Percentage difference in the highest EDIP quintiles: CRP 16% (-3%, 39%) P trend = 0.16; adiponectin -17% (-34%, 4%), P trend = 0.04; IL-6 32% (1%, 72%), P trend= 0.04; TNFa-R2 8% (1%, 14%), P trend = 0.0005. |
| Arouca 2018 ^28^  Europe  Nil funding | Assess association between MDS and its food-components with inflammatory biomarkers in adolescents of HELENA study | CSS (HELENA^29^); 2006-2007 | (n = 464) 12-17 y; M,F; health status NS | Cluster^b^ and individual inflammatory biomarkers^a^ | 2 x 24-hour recall  (validated: Flemish adolescents) | **MDS** *Trichopoulou et al.^24,30^* | *Index-inflammation analyses:* Multivariate and multiple linear regression analyses  *Adjusted variables:* adiposity, age, country, education, SES, sex, smoking | *Index-inflammation association:* MDS was positively associated with CRP in boys and negatively associated with sVCAM-1 in both boys and girls. MDS showed limited strength in assessing inflammation. |
| Baden 2019^31^  USA  Research institute | Assess association between plant-based indexes and several biomarkers predictive of cardiometabolic diseases | CSS (NHS II^9^); 1996–1999; 2010–2011 | (n = 831) 25–42 y; F; free from Cx, CVD, T2D | Adiponectin, CRP | 130-item FFQ (validated; 12 months) | **hPDI** *Satija 2016^19^*  **PDI** *Satija 2016^19^* | *Index-inflammation analyses:* Multivariate general linear regression models  *Adjusted variables:* age, alcohol, BMI, energy intake, HCL,HRT use, HTN, menopausal status, PA, smoking status, | *Index-inflammation association:* Higher hPDI scores were associated with lower hsCRP levels (−13.6% per 10-point increase in score) and higher adiponectin levels (3.0% per 10-point increase in score) (all P ≤ 0.025). After adjusting for BMI, no associations were observed for PDI with inflammatory markers. |
| Bérard 2020^32^  USA  Research institute | Assess associations between diet indexes and cardiometabolic outcomes and inflammatory profile in survivors of childhood leukemia. | CSS (PETALE^33^)  2013-2016 | (n = 241) 21.7 y (median age); M,F cALL survivors | Adiponectin, CRP, IL-6, TNF-α | 190-item FFQ (validated:) | **HEI-2015** *Krebs-Smith et al^34^*  **MEDAS** *Schröder et al.^35^* | *Index-inflammation analyses:* Multivariate logistic regression analyses  *Adjusted variables:* BMI, energy intake, sex, survival time. | *Index-inflammation association:* In cALL survivors, higher HEI-2015 scores were significantly associated with lower TNF-α levels (p=0.01) and higher MEDAS was significantly associated with higher adiponectin levels (p=0.004). |
| Bonaccio 2023 ^36^  Italy  Govt., research institute | Assess association between long-term changes in MED adherence and concurrent changes in CVD risk factors and biomarkers of chronic inflammation in Italian adults. | PCS (Moli-sani^37^)  2005-2010, 2017-2020 | (n = 2023) 35-78 y; M,F; health status NS | hs-CRP, inflammatory score^d^ | 2 x 188-item FFQ (SQ; validated: Italian population; 12 months) | **MDS** *Trichopoulou et al.^24^* | *Index-inflammation analyses:* Multivariable-adjusted linear regression models  *Adjusted variables:* age, education, energy intake, hx of CVD/ cancer, marital status, PA, SES, medication use, sex, smoking status. | *Index-inflammation association:* Higher MDS, over time was significantly associated with lower inflammatory score (β = -0.372,95% CI -0.720 to -0.025) and a decrease in chronic inflammation in an ageing population. |
| Carter 2010^38^  USA  Funding: NR | Assess the extent of association between MED diet and levels of atherothrombotic biomarkers in a population-based sample in the U.S. | CSS (NHANES III^39^)  1988-1994 | (n = 13197) 18-90 y; M, F; free from RA | CRP, Fibrinogen, Hcy, WBC | FFQ (validated: NHANES III^39^; 1 month)  24-hour recall interview | **MDS** *Panagiotakos et al.^40^* | *Index-inflammation analyses:* Multiple variable regression analysis  *Adjusted variables:* age, CHD risk, multivitamin/medication use, SES, sex. | *Index-inflammation association:* High MDS was significantly associated with lower CRP, Hcy, WBC, and fibrinogen all p = <0.05) in men and post-menopausal women, but not in pre-menopausal women. |
| Cervo 2021^41^  Australia  NHMRC Australia | Assess associations between MED diet adherence with cytokine levels, musculoskeletal health and incident falls, in older men. | PCS (CHAMP^42^)  2010-2013 | (n = 794) ≥ 70 y; M; health status NS | Cytokines (interleukins) | FFQ (DHQ by Dietitian) (validated) | **MEDI-LITE score** *Sofi et al.^43^* | *Index-inflammation analyses:* Multivariable linear regression analyses  *Adjusted variables:* age, birth country, body fat, comorbidities, energy intake, frailty status, NSAIDs use, PA, smoking status. | *Index-inflammation association:* MEDI-LITE score was statistically inversely associated with IL-7 in fully adjusted models. No significant associations were observed for other cytokines. |
| Chan 2019^44^  Hong Kong  Govt. | Assess association of several dietary patterns with CRP concentrations l in community-dwelling Chinese older adults. | PCS; 2001-2003 | (n = 2646) ≥65 y; M,F; community dwelling | CRP | FFQ  Dietary pattern derived by factor analysis | **DQI-I** *Kim* *et al.^23^*  **MDS** *Trichopoulou et al.^24^*  **MIND-S** *Morris et al.^45^*  **ODS** *Willcox et al.^46^* | *Index-inflammation analyses:* Ordinal regression analyses  *Adjusted variables:* age, alcohol use, BMI, chronic diseases, energy intake, PA, smoking status. | *Index-inflammation association:* CRP concentrations were significantly inversely associated with higher DQI-I (P = 0.001), MIND (P = 0.002), Okinawan (0.009) diet and MDS (0.015) scores in older Chinese community dwelling, males. No significant associations were observed for women. |
| Dai 2008^47^  USA  Research institute | Asses the association between MED diet adherence and circulating biomarkers of inflammation in twin males. | OS (THS^48^)  2002-2006 | (n = 345 twin pairs)  middle-aged; M; health status NS | hs-CRP, IL-6 | FFQ (SQ; 12 months) | **MDS** *Trichopoulou et al.^24^* | *Index-inflammation analyses:* Mixed effect regression analyses, adapted for twin studies  *Adjusted variables:* comorbidities, genetics, lifestyle factors, supplement/ medication use, SES | *Index-inflammation association:* Higher MDS was associated with significantly lower IL-6 but not CRP levels. Each one-unit increase in diet score was associated with a 9% (95% CI, 4.5 to 13.6) lower interleukin-6 level (P 0.001). |
| de Graaf 2022^49^  Netherlands  Research institute | Assess the association between adherence to Dutch dietary guidelines with inflammatory markers and GI symptoms in individuals with IBD or IBS | CSS (IBDSL^50^, MIBS^51^); period not specified | (n = 694) ≥18 y; M,F; IBD/IBS/ healthy control | fCal | FFQ (validated) | **DHD-2015** *Looman et al.^52^* | *Index-inflammation analyses:* Multivariable linear regression analysis  *Adjusted variables:* age, age at diagnosis, BMI, disease duration, medication, phenotype (IBD), subtype (IBS), sex, smoking. | *Index-inflammation association:* DHD-2015 was significantly associated with lower faecal calprotectin levels (b = 4.009, p=0.006) in individuals with IBD but not those with IBS or healthy controls. |
| Dias 2015^53^  Sweden  Research institute | Assess association between overall diet quality with biomarkers of chronic inflammation in middle-aged individuals | CSS (MDC study^54^)  1991-1996 | (n = 667) 63-68 y; M,F; health status NS | CRP, TNF-α, WBC | DHx and 168-item FFQ (validated) | **DQI-SNR** *Drake et al.^55^* | *Index-inflammation analyses:* Linear regression analysis  *Adjusted variables:* age, energy intake, sex, smoking. | *Index-inflammation association:* Higher DQI-SNR scores were significantly inversely associated with CRP, TNF-α levels (P <0.05) |
| English 2023^56^  Australia  Govt | Assess association between healthy dietary patterns and inflammatory biomarkers in Australian adults at varied levels of CVD risk | CSS; 2021-2022 | (n = 100) 49±13 y; M,F; diagnosed with ≥2 CVD risk factors | CRP, Lp-PLA_2_, PAF | 130-items FFQ (SQ; validated for Australia) | **DASH-S** *Gunther et al.^57^*  **HEIFA** *Roy et al.^58^*  **MEDAS** *Martínez-González et al.^59^* | *Inter-correlation analysis of dietary indexes:* pairwise Pearson’s correlation coefficients were calculated to assess inter-correlations between indexes.  *Index-inflammation analyses:* Multiple linear regression analyses  *Adjusted variables:* age, data collection year, level of risk, PA, sex, WC. | *Inter-index correlation:* All dietary indexes were significantly correlated with one another, with Pearson correlation coefficients ranging from 0.46 (medium) to 0.60 (strong) (all *P* < 0.001)  *Index-inflammation association:* All indexes were significantly inversely associated with CRP levels. A one-point increase in adherence to the DASH Index, HEIFA, MEDAS was associated with a 30%, 19%, and 22% reduction in CRP levels, respectively. |
| Fargnoli 2008^60^  USA  Research institute | Assess association between AHEI adherence with biomarkers of inflammation, endothelial dysfunction, and insulin resistance in women. | CSS (NHS^9^)  1990 | (n = 1,922) 30-55 y; F; free from CVD, DM | Adiponectin, CRP, E-selectin, ICAM-1, IL-6, TNF-α, VCAM-1 | FFQ (SQ; validated) | **AHEI** *McCullough et al.^17^* | *Index-inflammation analyses:* Simple linear regression models (crude analysis); multiple linear regression models  *Adjusted variables:* age, BMI, energy intake, PA, smoking. | *Index-inflammation association:* Highest (compared to lowest) AHEI scores had significantly higher adiponectin levels (32%) and lower levels of resistin (16%), CRP (41%) and E-selectin (19%) (P 0.01 for all). These associations remained significant after adjustment for potential confounders. No further significant associations were observed for other inflammatory markers. |
| Fernández-Barrés 2019^61^  Spain  Govt. | Assess association between maternal adherence to MED diet during pregnancy and the longitudinal BMI trajectories and cardiometabolic risk of offspring in early childhood | LCS; (INMA^62^);  2003-2008 | (n = 697 mother-child pairs) mother: gestational age ≥16 y, child: ≤4 y, M,F, health status NS | Adiponectin, CRP, IL-6 | 2 x 101-food items FFQ (validated for pregnant women in Spain) | **rMED score** *Buckland et al.^63^* | *Index-inflammation analyses:* Linear regression models  *Adjusted variables:* age, BMI, breastfeeding duration, education, GDM, PA, SES, smoking. | *Index-inflammation association:* No association between rMED score and inflammatory marker concentrations. Greater adherence to MED in third trimester of gestation was not associated with lower cardiometabolic risk in childhood. |
| Ford 2005^64^  USA  Funding source NR | Assess association between diet quality and CRP in a representative sample of US population | CSS (NHANES III^39^);  1988-1994 | (n = 13,811) ≥20 y; M,F; health status NS | CRP | 24-hour recall | **HEI** *Kennedy et al.^65^* | *Index-inflammation analyses:* Logistic regression analysis  *Adjusted variables:* age, aspirin/alcohol use, BMI, education, energy intake ethnicity, cotinine concentration, PA, sex, smoking, WHR. | *Index-inflammation association:* HEI score was inversely associated with high CRP concentration. Females in the highest quintile (comparing extreme quintiles) had 26% lower odds of elevated CRP levels (0.74; 95% CI): 0.57–0.96) |
| Fung 2005^66^  USA  Funding source NR | Assess association between several diet scores biomarkers of inflammation and endothelial dysfunction. | CSS (NHS^9^); 1990 | (n = 660) 43-69 y; F; free from chronic disease | CRP, E-selectin, IL-6 | 140-food items FFQ (validated: NHS^9^) | **AHEI** *McCullough et al.^17^*  **HEI** *Kennedy et al.^65^*  **aMED** *Fung 2005^66^*  **RFS** *Kant et al.^67^* | *Inter-correlation analysis of dietary indexes:* Spearman’s correlations coefficients were calculated to assess inter-correlations between indexes.  *Index-inflammation analyses:* Multivariate linear regression analyses  *Adjusted variables:* age, alcohol intake, BMI, energy intake, PA, smoking status. | *Inter-index correlation:* All dietary indexes were significantly strongly correlated with one another, with Spearman’s correlation coefficients ranging from 0.56 to 0.75 (all *P* < 0.001)  *Index-inflammation association:* Each one-unit score increase in AHEI and aMED was associated with significantly lower concentrations of inflammation. Highest quintile (comparing extreme quintiles) of aMED score was associated with lower concentrations of CRP (24%), IL-6 (16%) and E-selectin (13%) and the AHEI score was associated with sICAM-1 and sVCAM-1 (8%) and IL-6 (13%). |
| González-Ortiz 2020^68^  Sweden  Research institute | Assess association between adherence to plant-based diets and insulin sensitivity and inflammatory biomarkers in men with CKD. | CSS (ULSAM^69^);  1995-1999 | (n = 418) 70–71 y; M; free from DM | CRP, IL-6 | 7-day food diary | **PBDi** *Kim et al.^70^* | *Index-inflammation analyses:* Multivariable linear regression analyses  *Adjusted variables:* age, alcohol intake, BMI, BP, eGFR, energy intake, hx CVD, medication use, PA, smoking. | *Index-inflammation association:* Higher PBDi scores were significantly associated with lower CRP and IL-6 (P for trend <0.05). |
| Hayuningtyas 2021 ^71^  Indonesia  Nil funding | Examine overall diet quality of Indonesian children, using the HEI and to assess its association with serum adiponectin levels, considered an early marker of NCDs. | CSS (EJCS^72^);  2018-2019 | (n = 85) 3-5 y; M,F; free from chronic disease | Adiponectin | 2 x 24-hour recall | **HEI-2015** *Krebs-Smith et al^34^* | *Index-inflammation analyses:* Multiple linear regression analyses  *Adjusted variables:* exclusive breastfeeding hx | *Index-inflammation association:* Each one-point increase in the HEI 2015 score was significantly associated with a 0.115 μg/mL increase in the serum adiponectin level (after adjusting for exclusive breastfeeding hx) (β = 0.115; 95% CI = 0.010–0.221; p = 0.032). |
| Huang 2016^73^  USA  Research institute | Assess cross-sectional and longitudinal associations between dietary quality and several biomarkers of the brain-adipose axis | CSS (NHS II^9^);  1996–1999 and 2010–2011 | (n = 831) 32.8–53.4 y; F; health status NS | Adiponectin, CRP, IL-6 | FFQs (SQ; validated) | **AHEI-2010** *Chiuve et al. 2012^74^* | *Index-inflammation analyses:* General linear regression analyses  *Adjusted variables:* age, baseline dietary scores, energy intake, HRT use, menopausal status, PA, smoking status, time intervals. | *Index-inflammation association:* Females in the highest quintile of AHEI-2010 (comparing extreme quintiles) had significantly lower CRP (5%; P-trend = 0.02) and significantly higher adiponectin (8% P-trend = 0.0003). Associations for adiponectin remained significant after adjustment for BMI. |
| Huang 2023^75^  China  Research institute | Assess associations between plant-based diet indexes with serum levels of IR, inflammation, and cardiometabolic risk in a population sample (NHANES) | (NHANES^39^); 2003-2018 | (N = 34,785) 18–85 y; M,F; health status NS | CRP, WBC | 2 x 24-hour recall | **hPDI** *Satija 2016^19^*  **PDI** *Satija 2016^19^* | *Index-inflammation analyses:* Multiple linear regression analyses  *Adjusted variables:* age, alcohol use, BMI, CVD, DM, education, energy intake, ethnicity, HTN, marital status, SES, sex, smoking, survey cycle. | *Index-inflammation association:* Higher hPDI and PDI scores were associated with significantly lower CRP and WBC |
| Kanerva 2014^76^  Finland  Research institute | Assess association between a healthy Nordic diet (using BSDS) with concentrations of obesity-related inflammatory biomarkers. | CSS (DILGOM^72^);  2007 | (n = 4579) 25-74 y; M,F; health status NS | CRP, IL-6, TNF-α | 130-items FFQ (validated; 12 months) | **BSDS** *Kanerva et al.^77^* | *Index-inflammation analyses:* Linear regression analyses  *Adjusted variables:* age, energy intake, inflammation, lifestyle factors, obesity, sex, statin medication. | *Index-inflammation association:* Higher BSDS scores were significantly inversely associated with CRP levels (P 0.01). |
| Kant 2005^78^  USA  Research institute | Assess the ability of diet indexes to predict biomarkers of dietary intake, and chronic disease. | CSS (NHANES III^39^);  1988-1994 | (n = 15,979) ≥20 y; M,F; free from chronic disease | CRP, fibrinogen | 24-hour recall (by Dietitian) | **DDS-R** *Kant et al.^79^*  **HEI** *Kennedy et al.^80^*  **RFS** *Kant et al.^67^* | *Inter-correlation analysis of dietary indexes:* Pearson’s correlation coefficients were calculated to assess inter-correlations between indexes.  *Index-inflammation analyses:* Multiple linear regression models  *Adjusted variables:* age, education, alcohol intake, BMI, fasting, PA, race, sex, smoking status, supplement use. | *Inter-index correlation:* All dietary indexes were significantly strongly correlated with one another, with Spearman’s correlation coefficients ranging from 0.57 to 0.74 (all *P* < 0.001)  *Index-inflammation association:* HEI, RFS and DDS-R indexes were independent negative predictors of CRP and RFS was inversely associated with fibrinogen levels. RFS and DDS-R are two relatively simple dietary intake indexes for predicting biomarkers of disease risk. |
| Kharaty 2023^81^  Ireland  Research institute | Assess association between adherence to a plant-based diet and inflammation in a cohort of middle to older aged adults. | CSS (MCS II^82^);  2010-2011 | (n = 1986) 46–73 y; M,F; health status NS | Adiponectin, CRP, IL-6, leptin, PAI-1, resistin, TNF-α, | 150-item FFQ (SQ; validated; 12 months) | **PDI** *Satija 2016^19^*  **hPDI** *Satija 2016^19^* | *Index-inflammation analyses:* Linear regression models  *Adjusted variables:* alcohol intake, BMI, CVD, education, energy intake, HTN, medications, PA, smoking, T2D. | *Index-inflammation association:* Highest (compared to lowest) hPDI and PDI scores were significantly associated with lower CRP, IL-6 levels and increased adiponectin levels (P < 0.05). hPDI was significantly inversely associated with TNF-α (P < 0.05). |
| Ko 2016^83^  Korea  Research institute | Assessed cross-sectional associations between diet quality (using AHEI-2010 or DASH-S) and adipokines and inflammatory marker levels. As risk factors for CVD | CSS; 2009-2011 | (n = 196) 43-48 y; M,F; free from chronic disease | CRP, irisin, sICAM-1 | 100-item FFQ (SQ; validated) | **AHEI-2010** *Chiuve et al. 2012^74^*  **DASH-S** *Fung et al^22^* | *Index-inflammation analyses:* Multiple linear regression analysis  *Adjusted variables:* age, alcohol, BMI, education, energy intake, exercise, income, inflammation biomarkers, marriage, race, sex, smoking. | *Index-inflammation association:* CRP levels were statistically inversely associated with higher AHEI-2010 (β = -0.21, P = 0.018) and DASH-S (β = -0.20, P = 0.023) scores, even after controlling for BMI and total energy intake. |
| Lahoz 2018 ^84^  Spains  FundingNR | Assess association between each MEDAS question and the overall score with CRP levels in a Spanish general population. | CSS (SPREDIA-2^85^); period NR | (n = 1411) 45-74 y; M,F; free from chronic disease. | CRP | N/A | **MEDAS** *Schröder et al.^35^* | *Index-inflammation analyses:* Multiple linear regression  *Adjusted variables: a*ge, BMI, HTN, medications, MetS, sex. | *Index-inflammation association:* MEDAS was inversely correlated with CRP (β = 0.034, (CI 95% 0.062, -0.005), p = 0.019) even after adjusting concentrations even after adjustments. Findings suggest that higher adherence to MED as indicated by higher MEDAS is associated with lower CRP levels. |
| Li 2021^86^  Australia  Research institute | Assess (i) association between inflammatory biomarkers and diet scores (ii) associations between dietary score and all-cause mortality. | CSS (MCCS^87^); 2003-2007 | (n = 770) 69±8 y; M,F; free from inflammatory conditions | CRP, fCal, IFN-γ, IL-6, IL-8, IL-10, SAA, TNF-α | 144-item FFQ (SQ; validated) | **AHEI-2010** *Chiuve et al. 2012^74^*  **MDS** *Trichopoulou et al.^88^* | *Index-inflammation analyses:* Multivariate logistic regression analyses, multiple covariate-adjusted regression models.  *Adjusted variables:* age, BMI, country, energy intake, PA, sex, smoking. | *Index-inflammation association:* MDS and AHEI-2010 were associated with lower concentrations of CRP and IFN-γ. Additionally, the MDS was significantly inversely associated with IL-8 and TNF-α levels and the AHEI-2010 with IL-6 levels (all P < 0.05). |
| Li 2021^89^  USA  Nil funding | Assess associations between diet quality(using HEI-2015) with CVD risk factors, in individuals with long-standing SCI. | CSS; 2017-2019 | (n = 24) 45±12 y; M,F; SCI | CRP | 3 x 24-hour recall | **HEI-2015** *Krebs-Smith et al^34^* | *Index-inflammation analyses:* Multiple linear regression analyses  *Adjusted variables:* body fat %, level of injury, sex. | *Index-inflammation association:* Higher HEI-2015 was associated with a small inverse effect on CRP levels in individuals with SCI. Each 10-point increase in HEI-2015 score was associated with -0.02 mg/L (-0.26,0.22) decrease in CRP levels. |
| Li 2023^90^  China  Research institute | Assess associations of healthy dietary patterns with cardiometabolic biomarkers and all-cause mortality in individuals with prediabetes. | CCS (NHANES^39^); 1999-2014 | (n = 8363) ≥20 y; M,F; diagnosed diabetes | CRP | 3 x 24-hour recall | **aMED** *Fung 2005^66^*  **DASH-S** *Fung et al^22^*  **AHEI-2010** *Chiuve et al. 2012^74^*  **HEI-2015** *Krebs-Smith et al^34^* | *Inter-correlation analysis of dietary indexes:* Pearson correlation coefficients were calculated to assess inter-correlations between indexes.  *Index-inflammation analyses:* General linear regression analyses  *Adjusted variables:* age, alcohol intake, BMI, CVD, education, energy intake, ethnicity, HCL, hx HTN, income, marital status, PA, sex, smoking. | *Inter-index correlation:* All dietary indexes were significantly strongly correlated with one another, with Pearson correlation coefficients ranging from 0.68 to 0.76 (all *P* < 0.001).  *Index-inflammation association:* AHEI-2010, DASH-S, and HEI-2015 were associated with significantly lower CRP levels (all P-trend < 0.05). |
| Matsunaga 2021^91^  USA  Govt. | Assess association between dietary indexes with CRP levels in US adults with DM. | CSS (NHANES^39^)  2005-2010 | (n = 12,070) ≥20 y; M,F; health status NS | CRP | 2 x 24-hour recall | **DASH-S** *Matsunaga et al^92^*  **HEI-2015** *Krebs-Smith et al^34^* | *Index-inflammation analyses:* Multivariable logistic regression models  *Adjusted variables:* age, DM, ethnicity, HTN, sex, smoking status. | *Index-inflammation association:* CRP levels were significantly associated with the highest quintile (comparing extreme quintiles) for the DASH-S (OR 0.69, 95% CI 0.61 0.79) and HEI-2015 (OR 0.69, 95% CI 0.57 0.79) indexes, even after adjustments. |
| Mattei 2017^93^  USA  Research institute | Assess associations of diet quality scores with 2-y changes in cardiometabolic risk factors in adults. | LCS; (Boston Puerto Rican Health Study^94^)  2004-2010 | (n = 1194) 45-75 y; M,F; health status NS | CRP | FFQ (SQ; validated; 12 months) | **AHA-DS** *Mattei 2013^95^*  **DASH-S** *Fung et al.^22^*  **AHEI-2010** *Chiuve et al. 2012^74^*  **HEI-2010** *Guenther et al.^96^*  **MDS** *Trichopoulou et al.^24^* | *Inter-correlation analysis of dietary indexes:* diet quality scores were converted to z scores to enable comparisons between different diet quality indexes. Spearman’s correlation coefficients were calculated to assess inter-correlations between indexes.  *Index-inflammation analyses:* Multivariable linear regression  *Adjusted variables:* age, CVD, DM, education, energy intake, frequency of foods away from home, HTN, marital status, medications, PA, SES, sex, smoking. | *Inter-index correlation:* All dietary indexes were significantly correlated with one another, with Pearson correlation coefficients ranging from 0.37 (medium) to 0.68 (strong) (all *P* < 0.001).  *Index-inflammation association:* Higher MDS was significantly associated with lower CRP (-0.13 ± 0.03, P = 0.0002). No significant associations were observed for other indexes and CRP. |
| Mears 2019^97^  USA  Research institute | Assess associations between diet quality with body composition, IL-6 levels and OA severity in older African American adults with self-reported OA. | CSS; period NS | (n = 126) 60–87 y; F; diagnosed OA, OW/OB | IL-6 | 110-items FFQ | **AHEI-2010** *Chiuve et al. 2012^74^* | *Index-inflammation analyses:* Logistic regression models  *Adjusted variables:* OA severity | *Index-inflammation association:* IL-6 was significantly inversely associated with AHEI-2010 in African American females. Higher AHEI-2010 score attributed to a ~4% higher odds (OR 0.953, 95% CI 0.920–0.986, p = 0.006) of having lower IL-6.  Findings suggest AHEI-2010 was an independent predictor of IL-6 adjusting for OA severity |
| Millar 2021^98,99^  Ireland, USA, Switzerland  Research institute | Assess the cross-sectional association between HEI-2015 with inflammatory biomarkers, coagulation factors and WBC in a sample of adults aged 46–73 y. | CSS; (MCS II^82^)  2010-2011 | (n = 1862) 46-73 y; M,F; health status NS | Adiponectin, CRP, IL-6, PAI-1, TNF-α, WBC | 150-item FFQ (SQ; validated) | **DASH-S** *Fung et al.^22^*  **HEI-2015** *Krebs-Smith et al^34^*  **MDS** *Trichopoulou et al.^24^* | *Index-inflammation analyses:* Multivariate-adjusted liner regression analyses  *Adjusted variables:* age, BMI, cancer, CVD, education, energy intake, medications, PA, T2D, sex, smoking status. | *Index-inflammation association:* Higher MDS, DASH and HIE-2015 scores were associated with significantly lower CRP and IL-6 levels. MDS and DASH scores were significantly inversely associated with and TNF-α levels. |
| Mirrafiei 2023^100^  Iran  Nil funding | Assess association between FDII and CVD risk factors and inflammation in Iranian adults | CSS; 2018-2019 | (n = 816) 20-59 y; ^1,2,7,11,13^M,F; healthy | CRP | 3 x 24-hour recall | **FDII** *Salari-Moghaddam et al.,^101^* | *Index-inflammation analyses:* Multiple linear regression  *Adjusted variables:* age, BMI, education, energy intake marital status, occupation, PA, sex, smoking. | *Index-inflammation association:* No significant association between FDII and CRP levels were found. |
| Monfort-Pires 2014^102^  Brazil  Research institute | Assess association of B-HEI with markers of inflammation, insulin resistance and lipid profile in individuals at cardiometabolic risk. | CSS; 2008-2009 | (n = 204) 18-79y; M,F; diagnosed prediabetic | CRP, IL-6, TNF-α | 3 x 24-hour recall | **HEI-2010** *Guenther et al.^96^* | *Index-inflammation analyses:* Linear regression models  *Adjusted variables:* age, BMI, PA, sex. | *Index-inflammation association:* Higher HEI-2010 scores were significantly inversely associated with CRP levels (p=0.02) |
| Moradi 2020^103^  Iran  Nil funding | Assess association of Med diet adherence with renal function and CVD risk in patients with DM nephropathy | CSS; 2010-2013 | (n = 270) 65.9 ± 9.7 y; M,F; diagnosed DN | CRP | 168-item FFQ (SQ; validated; 12 months) | **MDS** *Trichopoulou et al.^24^* | *Index-inflammation analyses:* Multivariate-adjusted logistic regression models  *Adjusted variables:* age, drug use, energy intake, PA, SES, sex, smoking, WC. | *Index-inflammation association:* No significant associations between MDS and hs-CRP levels were found in patients with DN |
| Nilsson 2019^104^  Sweden  Nil funding | Assess associations between established dietary constructs with metabolic risk factors and inflammation older women. | CSS; period NR | (n = 112) 65–70 y; F; free from DM/CVD | Adiponectin,  CRP, fibrinogen | 6-day food diary | **DASH-S** *Fung et al.^22^* | *Index-inflammation analyses:* Analysis of covariance (ANCOVA)  *Adjusted variables:* energy intake, medication use, PA, WC. | *Index-inflammation association:* Higher DASH-S was significantly associated with higher adiponectin (P < 0.05). |
| Piccand 2019^105^  Switzerland  Research institute | Assess associations between dietary factors and inflammatory biomarkers | CSS (CoLaus study^106^)  2009-2012 | (n = 4027) 57.2±10.2 y; M,F; health status NS | CRP, IL-6, TNF-α | FFQ (SQ; validated; 1 month) | **AHEI** *McCullough et al.^17^*  **MDS** *Trichopoulou et al.^24^* | *Index-inflammation analyses:* Multivariable linear regression analyses  *Adjusted variables:* age, BMI, DM, education, energy intake, PA, sex, smoking. | *Index-inflammation association:* MDS and AHEI scores were significantly inversely associated with CRP levels (standardized regression score= −0·043 and −0·067, respectively, all P <0·01). No further associations were observed between indexes and inflammatory markers. |
| Piccirillo 2022^107^  Italy  Nil funding | Assess association between MED diet adherence and (i) inflammatory, lipid and glycaemic profile in patients with PAD; (ii) incidence of long-term major adverse CVD. | PCS; 2019-2020 | (n = 170) ≥ 45 y; M,F; PAD | CRP | 136-item FFQ (validated) | **MDS** *Martínez-González et al^108^* | *Index-inflammation analyses:* Multivariate regression analyses  *Adjusted variables:* age, BMI, CKD, DLD, DM, HTN, sex, smoking, vascular districts. | *Index-inflammation association:* Higher MDS was significantly associated with lower CRP concentrations (P = 0.0045). Findings suggest that the MED is associated with improved inflammatory status in participants with PAD. |
| Pocovi-Gerardino 2021^109^  Spain  Govt. | Assess association between MED Diet adherence and systemic lupus erythematosus clinical outcomes. | CSS; period NR | (n = 280) 46.9±12.8 y; M,F; diagnosed SLE | CRP | 24-hour recall | **MEDAS** *Schröder et al.^35^* | *Index-inflammation analyses:* Linear regression analyses  *Adjusted variables:* age, BMI, medical treatment, sex, smoking status. | *Index-inflammation association:* Higher adherence to Mediterranean DP measured by MEDAS was significantly inversely associated with CRP levels (P = 0.039; β = -0.055). High quality anti-inflammatory dietary patterns may contribute to management of SLE |
| Pourreza 2021^110^  Iran  Nil funding | Assess association of PDI with sleep quality and inflammatory biomarkers in OW/OB women | CSS; 2017-2019 | (n = 390) 18-48 y; F; OW/OB | CRP, IL-β1, TGF-β | 147-item FFQ (SQ; validated) | **PDI** *Satija 2016^19^*  **hPDI** Satija 2016^19^ | *Index-inflammation analyses:* Linear regression analyses  *Adjusted variables:* age, BMI, education, occupation, PA, smoking. | *Index-inflammation association:* Higher hPDI score (compared to lowest) was significantly and inversely associated with CRP levels (β = −0.14, 95% CI: −0.22,0.06, P = .001) and PDI was significantly associated with TGF-β (β = 2.04, 95% CI: 0.54,3.55, P = .008). |
| Rostgaard-Hansen 2023^111^  Germany  Research institute | Assess associations between diet quality scores and risk factors for cardiometabolic diseases, CRP, BP, WC, visceral and total fat mass. | CSS (DCH-NG^112^); 2015-2016 | (n = 450) 18–73 y; M,F; health status NS | CRP | 23-item FFQ (validated) | **DQS** *Toft et al.^113^* | *Index-inflammation analyses:* Linear regression models  *Adjusted variables:* age, education, PA, sex, smoking. | *Index-inflammation association:* High DQS was significantly associated with lower CRP concentrations (P = 0.0449) when adjusting for education, smoking habits and physical activity. |
| Sabia 2022 ^114^  Italy  Research institute | Assess association between MED diet adherence (using MEDAS) and CRP in Italian heavy smokers. | CCS; 2013-2016 | (n = 2438) 50-75 y; M,F; free from neoplasms | CRP | N/A | **MEDAS** *Schröder et al.^35^* | *Index-inflammation analyses:* Multivariate logistic regression models  *Adjusted variables:* age, BMI, FEV_1_%, chronic disease, medication use, sex, smoking status. | *Index-inflammation association:* MEDAS was significantly inversely associated with CRP levels, even after adjustments for BMI, in a population of heavy smokers. Each one-point increase in MEDAS score the odds of having elevated CRP levels (≥2 mg/L) decreased by 5% (0.95 (95% CI 0.91–0.99) |
| Savard 2021^115^  Canada  Research institute | Assess trimester-specific associations between inflammatory biomarkers concentrations and dietary indexes. | PCS (ANGE^116^); 2016-2017 | (n = 79) 32.1±3.7 y; F; pregnant, free from chronic disease. | Adiponectin, CRP, IL-6, leptin | 3 x 24-hour recall | **MDS** *Willet et al.^117^* | *Index-inflammation analyses:* Multivariate linear regression models  *Adjusted variables:* BMI, gestational weight gain, trimester. | *Index-inflammation association:* MDS was significantly associated with lower leptin levels (p < 0.001) in the second trimester. No further observations were observed. |
| Serrano-Martinez 2005^118^  Spain  Govt. | Assess associations between MED diet consumption and the production of inflammatory related molecules in coronary vessels | CSS; 2003 | (n = 24) 61.4±12.6 y; M,F; unstable angina. | TNF-α, VCAM-1 | 118-item FFQ (SQ; validated; 12 months) | **MDS** *Martínez-González et al^108^* | *Index-inflammation analyses:* Multiple linear regression models  *Adjusted variables:* BMI, coronary risk factors, sex. | *Index-inflammation association:* MDS was significantly inversely associated with TNF-α (β = -41.6 pg/ml (95% CI:–76.2 to –7.1), VCAM-1 (β = –35.1ng/ml (95% CI:–63.5, –6.7) coronary concentrations. Findings suggest that adherence to MED may be protective against inflammation of the coronary artery wall. |
| Sood 2022^119^  Australia  Research Institute | Assess associations between dietary indexes and insulin sensitivity, inflammatory biomarkers, and CVD risk factors in OW/OB adults without DM | CSS; period NR | (n = 65) 31.3±8.5 y; M,F; OW/OB without DM. | Adiponectin, chemokines, CRP, interleukins, MCP-1, NF_k_B, TNF-α | 3-day food diary | **MDS** *Panagiotakos et al.^120^* | *Index-inflammation analyses:* Multivariable regression analyses  *Adjusted variables:* age, body fat %, sex, WC | *Index-inflammation association:* Higher MDS was associated with lower NF_k_B (β = -2.147 (95% CI; -3.792, 0.501) P = 0.01) and higher adiponectin (β = 916.4 ng/mL (95% CI; 265.8, 1567.0) P = 0.007). No associations were observed. Findings suggest that MED adherence is associated with improved inflammation profile in adults with OW/OB |
| Tabung 2017^8^  USA  Research Institute | Assess the ability of dietary indexes to predict concentrations of inflammatory biomarkers. | CCS (NHS^9^, HPFS^10^)  1989–1990 | (n = 11,053) 25-42 y; M,F; health status NS | Adiponectin, CRP, IL-6, TNFα-R2 | FFQ | **EDII** *Tabung et al.^121^* | *Index-inflammation analyses:* Multivariable-adjusted linear regression analyses  *Adjusted variables:* age, energy intake, case/control status, chronic disease, HRT use, menopausal status, PA, smoking. | *Index-inflammation association:* Higher EDIP (indicative of a more pro-inflammatory diet) was associated with higher levels of CRP (38-60%), IL-6 (14-23%) TNFα-R2 (7-9%) and lower adiponectin levels (-16%). |
| Tertsunen 2022^122^  Finland  Nil funding | Assess associations of healthy Nordic diet with major CHD risk factors, carotid atherosclerosis and incident CHD in middle- to older-aged men in Finland | CS (KIHD^123^); 1984–1989 | (n = 2682) 42–60 y; M,F; free from CHD | CRP | 4-day food dairy | **BSDS** *Kanerva et al.^77^* | *Index-inflammation analyses:* Multivariable regression models  *Adjusted variables:* BMI, education, hx DM, income, marital status, medications, PA, smoking. | *Index-inflammation association:* Significant inverse association between HND score and CRP (multi variable-adjusted difference (comparing extreme quintiles): 0·66 mg/l, 95 % CI 0·11, 1·21 mg/l) |
| Vagianos 2021^124^  Canada  Pharmaceutical and research institutes | Assess associations between changes in dietary inflammatory potential and changes in intestinal inflammation, disease symptoms, or flares. | PCS; 2015-2017 | (n = 135) 45 y (mean age); M,F; diagnosed IBD (UC/CD) | fCal | 149-item FFQ (validated) | **EDIP** *Tabung et al.^7^* | *Index-inflammation analyses:* Multivariable ordinal logistic regression models  *Adjusted variables:* demographics, disease type and duration, smoking status. | *Index-inflammation association:* Change in EDIP was significantly associated with fCal levels. Each unit increase in EDII score (from baseline to follow-up) was associated with 3.1 times higher odds of having elevated fCAL levels above 250 μg/g. (P = 0.04) |
| Vahid 2022^125^  Iran  Nil funding | Assess association between HEI scores with the odds of OW/OB in Iranian adults. | CSS; period NS | (n = 1605) 18–81 y; M,F; free from chronic disease | CRP, IL-4, IL-1β, TNF-α | 124-item FFQ | **HEI-2015** *Krebs-Smith et al^34^* | *Index-inflammation analyses:* Multivariable logistic regression models  *Adjusted variables:* age, alcohol intake, BMI, DM, education, energy/nutrient intake, HTN, hx CVD, marital status, PA, sex, smoking. | *Index-inflammation association:* Higher HEI-2015 scores were significantly inversely associated with CRP, IL-4 and IL-1β concentrations in the multivariate-adjusted model (all *P* < 0.001). |
| Vahid 2023^126^  Europe, USA  Govt. | Assess association between diet quality and biomarkers of disease risk in a general adult population in Luxembourg. | CSS (ORISCAV- LUX 2^21^); 2016-2017 | (n = 1404) 25-79 y; M,F; health status NS | CRP | 174-food items FFQ (validated) | **AHEI** McCullough et al.^17^  **DASH-S** Fung et al^22^  **DQI-I** *Kim et al.^23^*  **MDS** *Trichopoulou et al.^30^* | *Inter-correlation analysis of dietary indexes:* Spearman’s correlation coefficients were calculated to assess inter-correlations between indexes.  *Index-inflammation analyses:* Multivariable general linear regression models  *Adjusted variables:* age, birth country, education, job, income, marital status, PA, sex, smoking status | *Inter-index correlation:* All dietary indexes were significantly strongly correlated with one another, with Spearman’s correlation coefficients ranging from 0.52 to 0.74 (all *P* < 0.001).  *Index-inflammation association:* No significant associations between any index and CRP concentrations were observed. |
| van der Pligt 2024^127^  Australia  Research Institute | Assess changes in adherence to dietary patterns across pregnancy and associations between diet quality and CRP levels in early and late pregnancy. | PCS (CPO study^128^)  2015-2017 | (n = 215) 31.5±3.9 y; F; pregnant | CRP | 2 x FFQ (DQES) | **DASH-S** *Fung et al.^22^*  **MDS** *Trichopoulou et al.^24^* | *Index-inflammation analyses:* Linear regression analysis  *Adjusted variables:* early pregnancy, GDM, gestational week, maternal age. | *Index-inflammation association:* In early pregnancy, CRP levels were significantly inversely associated with higher DASH-S (β = -0.04 (95% CI: -0.07, 0.00; P = 0.044) and higher MDS (β = -0.12 (95% CI: -0.21, -0.02; P = 0.023). Findings suggest that adherence to DASH and MED diets may beneficially affect inflammation in early pregnancy. |
| Vicente 2023^129^  Brazil  Research institute | Assess association of dietary indexes with an inflammatory biomarker. | Clinical trial; period NR | (n = 73) 63-89 y; M,F; non-frail, free from chronic disease | Anti-inflammatory index (IL-10:IL-6 ratio) | 2 x 24-hour recall | **MDS** *Trichopoulou et al.^24^* | *Index-inflammation analyses:* Multiple linear regression models  *Adjusted variables:* age, sex. | *Index-inflammation association:* Higher MDS was significantly associated with an anti-inflammatory profile. Each one-unit increase in the MDS was associated with a 0.25 unit increase in the Anti-inflammatory index. |
| Viscogliosi 2013^130^  Italy  Nil funding | Assess associations between MED diet adherence and metabolic syndrome, impaired fasting glucose, insulin resistance and microinflammation in subjects without DM or CVD. | CSS; 2012 | (n = 120) 59.8±10.2 y; M,F; free from chronic disease (except OB, HTN, DLD, IFG) | CRP | N/A | **MEDAS** *Martínez-González et al.^131^* | *Index-inflammation analyses:* Multivariable regression models  *Adjusted variables:* age, BMI, sex. | *Index-inflammation association:* Higher MDS (β= -0.082; 95% CI-0.125, -0.045; P <0.0001) showed statistically significant inverse associations with high CRP concentrations. |
| Waldeyer 2018^132^  Germany  Funding: NR | Assess association MED diet adherence (using MDS) with CAD severity. | PCS; 2015 | (n = 1,121) 67.7 y (mean); M,F; diagnosed CAD | CRP | FFQ | **MDS** *Stewart et al.^133^* | *Index-inflammation analyses:* Logistic regression analysis models  *Adjusted variables:* age, BMI, DM, DLD, HTN, medications, sex, smoking status. | *Index-inflammation association:* High MDS was significantly assocaited with lower CRP levels (P <0.001). Findings may contribute to the evidence for the protective effect of MED for prevention of CVD. |
| Wang 2023^134^  Australia  Research institute | Assess joint associations of dietary indexes with systemic inflammation, all-cause CVD, and cancer mortality risks by obesity status. | CSS (NHANES^39^);  1999–2010; 2015–2018 | (n = 27,915) ≥20 y; M,F; health status NS | CRP | 2 x 24-hour recall | **HEI-2015** *Krebs-Smith et al^34^*  **hPDI** *Satija 2016^19^*  **PDI** *Satija 2016^19^*  **PVDI** *Martínez-González et al.^135^* | *Index-inflammation analyses:* Multivariable ordinal logistic regression analyses  *Adjusted variables:* age, alcohol intake, BMI, chronic conditions, education, ethnicity, marital status, PA, SES, sex, smoking. | *Index-inflammation association:* Higher index scores, (compared to lowest) were significantly inversely associated with CRP levels in the fully adjusted model, in all participants. HEI-2015 (OR = 0.76, 95% CI 0.69, 0.84; P trend = < 0.001), PDI (OR = 0.83, 95% CI 0.75, 0.91; P trend = < 0.001), hPDI (OR = 0.79, 95% CI 0.71, 0.88; P trend = < 0.001), and PVD (OR = 0.85, 95% CI 0.75, 0.97; P trend = 0.02). |
| Weber 2024^136^  Germany  Govt. | Assess associations of adherence to dietary patterns with CVD risk factors, kidney function, and DM neuropathy among DM endotypes | CSS (GDS^137^); 2012-2021 | (n = 765) 48.7±13.3 y; M,F; diagnosed diabetes | CRP | 148-item FFQ (SQ; validated; 12 months) | **DASH-S** *Fung et al^22^*  **MDS** *Trichopoulou et al.^24^*  **hPDI** *Satija* 2016^19^  **PDI** *Satija* 2016^19^ | *Index-inflammation analyses:* Multivariable linear regression analyses  *Adjusted variables:* age, alcohol intake, BMI, BP, education, energy intake, sex, smoking status. | *Index-inflammation association:* Higher MDS, DASH-S, PDI and hPDI scores were inversely associated with CRP concentrations in individuals with MARD. β (95% CI): MDS -9.18 % (-15.61, -2.26); DASH-S -13.61 % (-24.17, -1.58); PDI -19.15 % (-34.28, -0.53); hPDI -16.10 % (-28.81, -1.12) |
| Whalen 2016^138^  USA  Research institute | Assess associations between diet pattern scores, and serum biomarkers of inflammation and lipid peroxidation. | CSS (MAP studies^4,5^); 1994–1997; 2002 | (n = 646) 30-74 y; M,F; free from CRC | CRP | 153-item FFQ (validated) | **MDS** *Whalen et al.^139^*  **Paleo diet score** *Whalen et al.^139^* | *Index-inflammation analyses:* General linear models  *Adjusted variables:* age, BMI, education, energy intake, ethnicity, HRT use, hx cancer, medication/ supplement use, PA, season, sex, smoking status. | *Index-inflammation association:* The multivariable-adjusted ORs comparing individuals in the highest quintile (comparing extreme quintiles) of the Palaeolithic and MDS were significantly associated with CRP levels (0.61 (95% CI: 0.36, 1.05; P-trend = 0.06) and 0.71 (95% CI: 0.42, 1.20; P-trend = 0.01) respectively). |

Abbreviations: AHEI, alternative healthy eating index; AIDI-20**,** anti-inflammatory diet index; aMED, alternate Mediterranean diet index; BDSD, Baltic sea diet score; BMI, body mass index; cALL, childhood acute lymphoblastic leukemia; CECP, Calcium and Colorectal Epithelial Cell Proliferation; CRP, C-reactive protein; CSS, cross-sectional study; DASH-S, Dietary Approaches to Stop Hypertension Score; DHD, Dutch healthy diet-index; DIS, dietary inflammation score; DP, dietary pattern; DQI-I, diet quality index international; DQS, diet quality score; EDII, empirical dietary inflammatory index; EDIP, empirical dietary inflammatory index pattern; F; female; fCal, faecal calprotectin; FFQ, food frequency questionnaires; govt, government; Hcy, homocysteine; HEI, healthy eating index; HEIFA, healthy eating index for Australians; hPDI, healthy plant-based diet index; HPFS, health professionals follow-up study; HRT, hormone replacement therapy; CRP, high sensitivity C-reactive protein; HTN, hypertension; ICAM, intercellular adhesion molecule; IL, interleukin; Lp-PLA2, lipoprotein-associated phospholipase A_2_; M, male; MAPs, markers of adenomatous polyps; MDS, Mediterranean diet score; MEDAS, Mediterranean diet adherence screener;  MEDI-LITE: literature-derived Mediterranean diet; MIND-S, Mediterranean-DASH diet intervention for neurodegenerative delay score; NHS, nurses' health study; NR, not reported; NS, not specified; ODS, Okinawan diet score; PA, physical activity; PAF, platelet-activating factor; Paleo, paleolithic; PBDi, plant-based diet index; PCS, prospective cohort study; PDI, plant-based diet index; PLCO, prostate, lung, colorectal and ovarian; PVDI, pro-vegetarian diet index; RCI, recommendation compliance index; REGARDS, reasons for geographic and racial differences in stroke study; RFS, recommended food score; rMED, relative Mediterranean diet; RRR, reduced rank regression; SA, self-administered; SES, socio-economic status; SNR, Swedish nutrition recommendations; s ICAM, soluble intercellular adhesion molecule; SQ, semi-quantitative; T2D, type 2 diabetes mellitus; TGF- β, Transforming growth factor β; TNFαR2, tumor necrosis factor alpha receptor-2; y, year; VCAM, vascular cell adhesion molecule; WBC, white blood cells; y, years

|  | Indexes based on inflammatory potential of diet | | | | | | Indexes Based on Dietary Guidelines/Recommendations | | | | | | | | | | | | | | Indexes Based on Therapeutic Diets | | | | | Indexes Based on Dietary Patterns | | | | | | | | | | | | | | | | | |
| --- | --- | --- | --- | --- | --- | --- | --- | --- | --- | --- | --- | --- | --- | --- | --- | --- | --- | --- | --- | --- | --- | --- | --- | --- | --- | --- | --- | --- | --- | --- | --- | --- | --- | --- | --- | --- | --- | --- | --- | --- | --- | --- | --- |
| Index name and reference | **AIDI-20** Kaluza et al.^1^ | **DIS** Byrd et al.^2^ | **EDII/EDIP** Tabung et al.^7,8^ | **FDII** Salari-Moghaddam et al.^101^ | **IFI** Riboldi et al.^11^ | **PAIFIS** Azevedo-Garcia et al.^13^ | **AHEI** McCullough et al.^17,140^ | **AHEI-2010** Chiuve et al.^74^ | **HEI** Kennedy et al.^65^ | **HEI-2010** Guenther et al.^96^ | **HEI-2015** Krebs-Smith et al.^34^ | **HEIFA** Roy et al.^58^ | **DDS-R** Kant et al.^79^ | **DQI-I** Kim et al.^23^ | **DQI-SNR** Drake et al.^55^ | **DQS** Toftet al.^113^ | **DHD-2015** Looman et al.^52^ | **RCI** Alkerwi et al.^25^ | **RFS** Kant et al.^67^ | **RFS** McCullough et al.^17^ | **AHA-DS** Maatei et al.^95^ | **DASH-S** Fung et al.^22^ | **DASH-S** Gunther et al.^57^ | **DASH-S** Matsunaga et al.^92^ | **MIND-S** Morris et al.^45^ | **BSDS** Kanerva et al.^77^ | **MDS** Martínez-González et al^108^ | **MDS** Panagiotakos et al.^120^ | **MDS** Stewart et al.^133^ | **MDS** Trichopoulou et al. ^24,88^ | **MDS** Trichopoulou et al. ^24^ | **MDS** Whalen et al.^139^ | **MDS** Willet et al.^117^ | **aMED** Fung 2005.^66^ | **rMED** Buckland et al.^63^ | **MEDAS** Martínez. et al.^131^ ^35^ | **MEDI-LITE** Sofi et al.^43^ | **ODS** Willcox et al.^46^ | **Paleo diet score** Whalen et al.^139^ | **PBDi** Kim et al.^70^ | **PDI** Satija et al.^19^ | **hPDI** Satija et al.^19^ | **PVDI** Martínez-González et al.^135^ |
| No. of components | 20 | 19 | 18 | 28 | 18 | 7 | 9 | 11 | 10 | 12 | 13 | 10 | 5 | 16 | 6 | 4 | 13 | 13 | 6 | 5 | 11 | 8 | 8 | 9 | 9 | 9 | 8 | 11 | 7 | 9 | 9 | 11 | 11 | 9 | 8 | 14 | 9 | 16 | 14 | 14 | 18 | 18 | 12 |
| **DIET DIVERSITY** |  |  |  |  |  |  |  |  |  |  |  |  |  |  |  |  |  |  |  |  |  |  |  |  |  |  |  |  |  |  |  |  |  |  |  |  |  |  |  |  |  |  |  |
| Overall diet diversity |  |  |  |  |  |  |  |  |  |  |  |  |  |  |  |  |  |  |  |  |  |  |  |  |  |  |  |  |  |  |  |  |  |  |  |  |  |  |  |  |  |  |  |
| Fruit & vegetable variety |  |  |  |  |  |  |  |  |  |  |  |  |  |  |  |  |  |  |  |  |  |  |  |  |  |  |  |  |  |  |  |  |  |  |  |  |  |  |  |  |  |  |  |
| **FRUIT** |  |  |  |  |  |  |  |  |  |  |  |  |  |  |  |  |  |  |  |  |  |  |  |  |  |  |  |  |  |  |  |  |  |  |  |  |  |  |  |  |  |  |  |
| Fruits |  |  |  |  |  |  |  |  |  |  |  |  |  |  |  |  |  |  |  |  |  |  |  |  |  |  |  |  |  |  |  |  |  |  |  |  |  |  |  |  |  |  |  |
| Whole fruits |  |  |  |  |  |  |  |  |  |  |  |  |  |  |  |  |  |  |  |  |  |  |  |  |  |  |  |  |  |  |  |  |  |  |  |  |  |  |  |  |  |  |  |
| Fruit (incl. nuts) |  |  |  |  |  |  |  |  |  |  |  |  |  |  |  |  |  |  |  |  |  |  |  |  |  |  |  |  |  |  |  |  |  |  |  |  |  |  |  |  |  |  |  |
| Citrus fruits |  |  |  |  |  |  |  |  |  |  |  |  |  |  |  |  |  |  |  |  |  |  |  |  |  |  |  |  |  |  |  |  |  |  |  |  |  |  |  |  |  |  |  |
| Red fruits |  |  |  |  |  |  |  |  |  |  |  |  |  |  |  |  |  |  |  |  |  |  |  |  |  |  |  |  |  |  |  |  |  |  |  |  |  |  |  |  |  |  |  |
| Berries |  |  |  |  |  |  |  |  |  |  |  |  |  |  |  |  |  |  |  |  |  |  |  |  |  |  |  |  |  |  |  |  |  |  |  |  |  |  |  |  |  |  |  |
| Apples & berries |  |  |  |  |  |  |  |  |  |  |  |  |  |  |  |  |  |  |  |  |  |  |  |  |  |  |  |  |  |  |  |  |  |  |  |  |  |  |  |  |  |  |  |
| Dried fruits |  |  |  |  |  |  |  |  |  |  |  |  |  |  |  |  |  |  |  |  |  |  |  |  |  |  |  |  |  |  |  |  |  |  |  |  |  |  |  |  |  |  |  |
| **COMBINED FRUIT/VEG** |  |  |  |  |  |  |  |  |  |  |  |  |  |  |  |  |  |  |  |  |  |  |  |  |  |  |  |  |  |  |  |  |  |  |  |  |  |  |  |  |  |  |  |
| Total fruits & vegetables |  |  |  |  |  |  |  |  |  |  |  |  |  |  |  |  |  |  |  |  |  |  |  |  |  |  |  |  |  |  |  |  |  |  |  |  |  |  |  |  |  |  |  |
| Dark yellow veg & fruits |  |  |  |  |  |  |  |  |  |  |  |  |  |  |  |  |  |  |  |  |  |  |  |  |  |  |  |  |  |  |  |  |  |  |  |  |  |  |  |  |  |  |  |
| **VEGETABLES** |  |  |  |  |  |  |  |  |  |  |  |  |  |  |  |  |  |  |  |  |  |  |  |  |  |  |  |  |  |  |  |  |  |  |  |  |  |  |  |  |  |  |  |
| Vegetables |  |  |  |  |  |  |  |  |  |  |  |  |  |  |  |  |  |  |  |  |  |  |  |  |  |  |  |  |  |  |  |  |  |  |  |  |  |  |  |  |  |  |  |
| Veg/legumes |  |  |  |  |  |  |  |  |  |  |  |  |  |  |  |  |  |  |  |  |  |  |  |  |  |  |  |  |  |  |  |  |  |  |  |  |  |  |  |  |  |  |  |
| Green leafy vegetables |  |  |  |  |  |  |  |  |  |  |  |  |  |  |  |  |  |  |  |  |  |  |  |  |  |  |  |  |  |  |  |  |  |  |  |  |  |  |  |  |  |  |  |
| Yellow/Orange vegetables |  |  |  |  |  |  |  |  |  |  |  |  |  |  |  |  |  |  |  |  |  |  |  |  |  |  |  |  |  |  |  |  |  |  |  |  |  |  |  |  |  |  |  |
| Green vegetables |  |  |  |  |  |  |  |  |  |  |  |  |  |  |  |  |  |  |  |  |  |  |  |  |  |  |  |  |  |  |  |  |  |  |  |  |  |  |  |  |  |  |  |
| Tomatoes |  |  |  |  |  |  |  |  |  |  |  |  |  |  |  |  |  |  |  |  |  |  |  |  |  |  |  |  |  |  |  |  |  |  |  |  |  |  |  |  |  |  |  |
| Cruciferous vegetables |  |  |  |  |  |  |  |  |  |  |  |  |  |  |  |  |  |  |  |  |  |  |  |  |  |  |  |  |  |  |  |  |  |  |  |  |  |  |  |  |  |  |  |
| Potatoes |  |  |  |  |  |  |  |  |  |  |  |  |  |  |  |  |  |  |  |  |  |  |  |  |  |  |  |  |  |  |  |  |  |  |  |  |  |  |  |  |  |  |  |
| Sweet potatoes |  |  |  |  |  |  |  |  |  |  |  |  |  |  |  |  |  |  |  |  |  |  |  |  |  |  |  |  |  |  |  |  |  |  |  |  |  |  |  |  |  |  |  |
| Other vegetables |  |  |  |  |  |  |  |  |  |  |  |  |  |  |  |  |  |  |  |  |  |  |  |  |  |  |  |  |  |  |  |  |  |  |  |  |  |  |  |  |  |  |  |
| Pickled vegetables |  |  |  |  |  |  |  |  |  |  |  |  |  |  |  |  |  |  |  |  |  |  |  |  |  |  |  |  |  |  |  |  |  |  |  |  |  |  |  |  |  |  |  |
| **GRAINS** |  |  |  |  |  |  |  |  |  |  |  |  |  |  |  |  |  |  |  |  |  |  |  |  |  |  |  |  |  |  |  |  |  |  |  |  |  |  |  |  |  |  |  |
| Total grains |  |  |  |  |  |  |  |  |  |  |  |  |  |  |  |  |  |  |  |  |  |  |  |  |  |  |  |  |  |  |  |  |  |  |  |  |  |  |  |  |  |  |  |
| Whole grains/Cereals |  |  |  |  |  |  |  |  |  |  |  |  |  |  |  |  |  |  |  |  |  |  |  |  |  |  |  |  |  |  |  |  |  |  |  |  |  |  |  |  |  |  |  |
| Whole grains/Cereals/Nuts |  |  |  |  |  |  |  |  |  |  |  |  |  |  |  |  |  |  |  |  |  |  |  |  |  |  |  |  |  |  |  |  |  |  |  |  |  |  |  |  |  |  |  |
| Grains& starches |  |  |  |  |  |  |  |  |  |  |  |  |  |  |  |  |  |  |  |  |  |  |  |  |  |  |  |  |  |  |  |  |  |  |  |  |  |  |  |  |  |  |  |
| Rice |  |  |  |  |  |  |  |  |  |  |  |  |  |  |  |  |  |  |  |  |  |  |  |  |  |  |  |  |  |  |  |  |  |  |  |  |  |  |  |  |  |  |  |
| Wheat, barley, other grains |  |  |  |  |  |  |  |  |  |  |  |  |  |  |  |  |  |  |  |  |  |  |  |  |  |  |  |  |  |  |  |  |  |  |  |  |  |  |  |  |  |  |  |
| Wheat flour |  |  |  |  |  |  |  |  |  |  |  |  |  |  |  |  |  |  |  |  |  |  |  |  |  |  |  |  |  |  |  |  |  |  |  |  |  |  |  |  |  |  |  |
| Refined grains/starches |  |  |  |  |  |  |  |  |  |  |  |  |  |  |  |  |  |  |  |  |  |  |  |  |  |  |  |  |  |  |  |  |  |  |  |  |  |  |  |  |  |  |  |
| **LEGUMES** |  |  |  |  |  |  |  |  |  |  |  |  |  |  |  |  |  |  |  |  |  |  |  |  |  |  |  |  |  |  |  |  |  |  |  |  |  |  |  |  |  |  |  |
| Legumes |  |  |  |  |  |  |  |  |  |  |  |  |  |  |  |  |  |  |  |  |  |  |  |  |  |  |  |  |  |  |  |  |  |  |  |  |  |  |  |  |  |  |  |
| **NUTS** |  |  |  |  |  |  |  |  |  |  |  |  |  |  |  |  |  |  |  |  |  |  |  |  |  |  |  |  |  |  |  |  |  |  |  |  |  |  |  |  |  |  |  |
| Nuts /seeds |  |  |  |  |  |  |  |  |  |  |  |  |  |  |  |  |  |  |  |  |  |  |  |  |  |  |  |  |  |  |  |  |  |  |  |  |  |  |  |  |  |  |  |
| Linseeds |  |  |  |  |  |  |  |  |  |  |  |  |  |  |  |  |  |  |  |  |  |  |  |  |  |  |  |  |  |  |  |  |  |  |  |  |  |  |  |  |  |  |  |
| Nuts incl. legumes/ seeds |  |  |  |  |  |  |  |  |  |  |  |  |  |  |  |  |  |  |  |  |  |  |  |  |  |  |  |  |  |  |  |  |  |  |  |  |  |  |  |  |  |  |  |
| **OTHER** |  |  |  |  |  |  |  |  |  |  |  |  |  |  |  |  |  |  |  |  |  |  |  |  |  |  |  |  |  |  |  |  |  |  |  |  |  |  |  |  |  |  |  |
| Low fat mixed dishes |  |  |  |  |  |  |  |  |  |  |  |  |  |  |  |  |  |  |  |  |  |  |  |  |  |  |  |  |  |  |  |  |  |  |  |  |  |  |  |  |  |  |  |
| Carbohydrate rich foods |  |  |  |  |  |  |  |  |  |  |  |  |  |  |  |  |  |  |  |  |  |  |  |  |  |  |  |  |  |  |  |  |  |  |  |  |  |  |  |  |  |  |  |
| Sofrito |  |  |  |  |  |  |  |  |  |  |  |  |  |  |  |  |  |  |  |  |  |  |  |  |  |  |  |  |  |  |  |  |  |  |  |  |  |  |  |  |  |  |  |
| Other animal-based foods |  |  |  |  |  |  |  |  |  |  |  |  |  |  |  |  |  |  |  |  |  |  |  |  |  |  |  |  |  |  |  |  |  |  |  |  |  |  |  |  |  |  |  |
| **MEAT/ MEAT ALTERNATIVE** |  |  |  |  |  |  |  |  |  |  |  |  |  |  |  |  |  |  |  |  |  |  |  |  |  |  |  |  |  |  |  |  |  |  |  |  |  |  |  |  |  |  |  |
| Total protein |  |  |  |  |  |  |  |  |  |  |  |  |  |  |  |  |  |  |  |  |  |  |  |  |  |  |  |  |  |  |  |  |  |  |  |  |  |  |  |  |  |  |  |
| Meat (unspecified) |  |  |  |  |  |  |  |  |  |  |  |  |  |  |  |  |  |  |  |  |  |  |  |  |  |  |  |  |  |  |  |  |  |  |  |  |  |  |  |  |  |  |  |
| Red & processed meat |  |  |  |  |  |  |  |  |  |  |  |  |  |  |  |  |  |  |  |  |  |  |  |  |  |  |  |  |  |  |  |  |  |  |  |  |  |  |  |  |  |  |  |
| Red meat |  |  |  |  |  |  |  |  |  |  |  |  |  |  |  |  |  |  |  |  |  |  |  |  |  |  |  |  |  |  |  |  |  |  |  |  |  |  |  |  |  |  |  |
| Pork |  |  |  |  |  |  |  |  |  |  |  |  |  |  |  |  |  |  |  |  |  |  |  |  |  |  |  |  |  |  |  |  |  |  |  |  |  |  |  |  |  |  |  |
| Processed meat |  |  |  |  |  |  |  |  |  |  |  |  |  |  |  |  |  |  |  |  |  |  |  |  |  |  |  |  |  |  |  |  |  |  |  |  |  |  |  |  |  |  |  |
| Hot dog |  |  |  |  |  |  |  |  |  |  |  |  |  |  |  |  |  |  |  |  |  |  |  |  |  |  |  |  |  |  |  |  |  |  |  |  |  |  |  |  |  |  |  |
| Organ meat |  |  |  |  |  |  |  |  |  |  |  |  |  |  |  |  |  |  |  |  |  |  |  |  |  |  |  |  |  |  |  |  |  |  |  |  |  |  |  |  |  |  |  |
| Fish/seafood |  |  |  |  |  |  |  |  |  |  |  |  |  |  |  |  |  |  |  |  |  |  |  |  |  |  |  |  |  |  |  |  |  |  |  |  |  |  |  |  |  |  |  |
| Oily fish |  |  |  |  |  |  |  |  |  |  |  |  |  |  |  |  |  |  |  |  |  |  |  |  |  |  |  |  |  |  |  |  |  |  |  |  |  |  |  |  |  |  |  |
| Seafood & plant proteins |  |  |  |  |  |  |  |  |  |  |  |  |  |  |  |  |  |  |  |  |  |  |  |  |  |  |  |  |  |  |  |  |  |  |  |  |  |  |  |  |  |  |  |
| Lean meat/poultry/fish/eggs |  |  |  |  |  |  |  |  |  |  |  |  |  |  |  |  |  |  |  |  |  |  |  |  |  |  |  |  |  |  |  |  |  |  |  |  |  |  |  |  |  |  |  |
| Poultry |  |  |  |  |  |  |  |  |  |  |  |  |  |  |  |  |  |  |  |  |  |  |  |  |  |  |  |  |  |  |  |  |  |  |  |  |  |  |  |  |  |  |  |
| White:Red meat ratio |  |  |  |  |  |  |  |  |  |  |  |  |  |  |  |  |  |  |  |  |  |  |  |  |  |  |  |  |  |  |  |  |  |  |  |  |  |  |  |  |  |  |  |
| Eggs |  |  |  |  |  |  |  |  |  |  |  |  |  |  |  |  |  |  |  |  |  |  |  |  |  |  |  |  |  |  |  |  |  |  |  |  |  |  |  |  |  |  |  |
| Plant proteins |  |  |  |  |  |  |  |  |  |  |  |  |  |  |  |  |  |  |  |  |  |  |  |  |  |  |  |  |  |  |  |  |  |  |  |  |  |  |  |  |  |  |  |
| **DAIRY** |  |  |  |  |  |  |  |  |  |  |  |  |  |  |  |  |  |  |  |  |  |  |  |  |  |  |  |  |  |  |  |  |  |  |  |  |  |  |  |  |  |  |  |
| Dairy |  |  |  |  |  |  |  |  |  |  |  |  |  |  |  |  |  |  |  |  |  |  |  |  |  |  |  |  |  |  |  |  |  |  |  |  |  |  |  |  |  |  |  |
| Full fat dairy/ cheese |  |  |  |  |  |  |  |  |  |  |  |  |  |  |  |  |  |  |  |  |  |  |  |  |  |  |  |  |  |  |  |  |  |  |  |  |  |  |  |  |  |  |  |
| Low fat dairy/cheese |  |  |  |  |  |  |  |  |  |  |  |  |  |  |  |  |  |  |  |  |  |  |  |  |  |  |  |  |  |  |  |  |  |  |  |  |  |  |  |  |  |  |  |
| Dairy & beans |  |  |  |  |  |  |  |  |  |  |  |  |  |  |  |  |  |  |  |  |  |  |  |  |  |  |  |  |  |  |  |  |  |  |  |  |  |  |  |  |  |  |  |
| **FATS/ OILS** |  |  |  |  |  |  |  |  |  |  |  |  |  |  |  |  |  |  |  |  |  |  |  |  |  |  |  |  |  |  |  |  |  |  |  |  |  |  |  |  |  |  |  |
| Dietary fats & oils |  |  |  |  |  |  |  |  |  |  |  |  |  |  |  |  |  |  |  |  |  |  |  |  |  |  |  |  |  |  |  |  |  |  |  |  |  |  |  |  |  |  |  |
| Oils (nuts, seeds, fish, veg) |  |  |  |  |  |  |  |  |  |  |  |  |  |  |  |  |  |  |  |  |  |  |  |  |  |  |  |  |  |  |  |  |  |  |  |  |  |  |  |  |  |  |  |
| Vegetable oils |  |  |  |  |  |  |  |  |  |  |  |  |  |  |  |  |  |  |  |  |  |  |  |  |  |  |  |  |  |  |  |  |  |  |  |  |  |  |  |  |  |  |  |
| Olive oil |  |  |  |  |  |  |  |  |  |  |  |  |  |  |  |  |  |  |  |  |  |  |  |  |  |  |  |  |  |  |  |  |  |  |  |  |  |  |  |  |  |  |  |
| Olive oil as main fat |  |  |  |  |  |  |  |  |  |  |  |  |  |  |  |  |  |  |  |  |  |  |  |  |  |  |  |  |  |  |  |  |  |  |  |  |  |  |  |  |  |  |  |
| Animal fats (butter/cream) |  |  |  |  |  |  |  |  |  |  |  |  |  |  |  |  |  |  |  |  |  |  |  |  |  |  |  |  |  |  |  |  |  |  |  |  |  |  |  |  |  |  |  |
| Hydrogenated oils |  |  |  |  |  |  |  |  |  |  |  |  |  |  |  |  |  |  |  |  |  |  |  |  |  |  |  |  |  |  |  |  |  |  |  |  |  |  |  |  |  |  |  |
| Hydrogenated fats |  |  |  |  |  |  |  |  |  |  |  |  |  |  |  |  |  |  |  |  |  |  |  |  |  |  |  |  |  |  |  |  |  |  |  |  |  |  |  |  |  |  |  |
| **DISCRETIONARY** |  |  |  |  |  |  |  |  |  |  |  |  |  |  |  |  |  |  |  |  |  |  |  |  |  |  |  |  |  |  |  |  |  |  |  |  |  |  |  |  |  |  |  |
| Added sugar |  |  |  |  |  |  |  |  |  |  |  |  |  |  |  |  |  |  |  |  |  |  |  |  |  |  |  |  |  |  |  |  |  |  |  |  |  |  |  |  |  |  |  |
| Sweets, desserts, pastries |  |  |  |  |  |  |  |  |  |  |  |  |  |  |  |  |  |  |  |  |  |  |  |  |  |  |  |  |  |  |  |  |  |  |  |  |  |  |  |  |  |  |  |
| Added fat (diet moderation) |  |  |  |  |  |  |  |  |  |  |  |  |  |  |  |  |  |  |  |  |  |  |  |  |  |  |  |  |  |  |  |  |  |  |  |  |  |  |  |  |  |  |  |
| Candies |  |  |  |  |  |  |  |  |  |  |  |  |  |  |  |  |  |  |  |  |  |  |  |  |  |  |  |  |  |  |  |  |  |  |  |  |  |  |  |  |  |  |  |
| Chocolate |  |  |  |  |  |  |  |  |  |  |  |  |  |  |  |  |  |  |  |  |  |  |  |  |  |  |  |  |  |  |  |  |  |  |  |  |  |  |  |  |  |  |  |
| Pizza |  |  |  |  |  |  |  |  |  |  |  |  |  |  |  |  |  |  |  |  |  |  |  |  |  |  |  |  |  |  |  |  |  |  |  |  |  |  |  |  |  |  |  |
| Snacks |  |  |  |  |  |  |  |  |  |  |  |  |  |  |  |  |  |  |  |  |  |  |  |  |  |  |  |  |  |  |  |  |  |  |  |  |  |  |  |  |  |  |  |
| Chips/ Fried potatoes |  |  |  |  |  |  |  |  |  |  |  |  |  |  |  |  |  |  |  |  |  |  |  |  |  |  |  |  |  |  |  |  |  |  |  |  |  |  |  |  |  |  |  |
| Fats/sugar/sodium/alcohol |  |  |  |  |  |  |  |  |  |  |  |  |  |  |  |  |  |  |  |  |  |  |  |  |  |  |  |  |  |  |  |  |  |  |  |  |  |  |  |  |  |  |  |
| **ALCOHOL** |  |  |  |  |  |  |  |  |  |  |  |  |  |  |  |  |  |  |  |  |  |  |  |  |  |  |  |  |  |  |  |  |  |  |  |  |  |  |  |  |  |  |  |
| Alcohol |  |  |  |  |  |  |  |  |  |  |  |  |  |  |  |  |  |  |  |  |  |  |  |  |  |  |  |  |  |  |  |  |  |  |  |  |  |  |  |  |  |  |  |
| Wine/ Red wine |  |  |  |  |  |  |  |  |  |  |  |  |  |  |  |  |  |  |  |  |  |  |  |  |  |  |  |  |  |  |  |  |  |  |  |  |  |  |  |  |  |  |  |
| Beer |  |  |  |  |  |  |  |  |  |  |  |  |  |  |  |  |  |  |  |  |  |  |  |  |  |  |  |  |  |  |  |  |  |  |  |  |  |  |  |  |  |  |  |
| **BEVERAGES** |  |  |  |  |  |  |  |  |  |  |  |  |  |  |  |  |  |  |  |  |  |  |  |  |  |  |  |  |  |  |  |  |  |  |  |  |  |  |  |  |  |  |  |
| SSB |  |  |  |  |  |  |  |  |  |  |  |  |  |  |  |  |  |  |  |  |  |  |  |  |  |  |  |  |  |  |  |  |  |  |  |  |  |  |  |  |  |  |  |
| Water |  |  |  |  |  |  |  |  |  |  |  |  |  |  |  |  |  |  |  |  |  |  |  |  |  |  |  |  |  |  |  |  |  |  |  |  |  |  |  |  |  |  |  |
| Tea |  |  |  |  |  |  |  |  |  |  |  |  |  |  |  |  |  |  |  |  |  |  |  |  |  |  |  |  |  |  |  |  |  |  |  |  |  |  |  |  |  |  |  |
| Herbal tea |  |  |  |  |  |  |  |  |  |  |  |  |  |  |  |  |  |  |  |  |  |  |  |  |  |  |  |  |  |  |  |  |  |  |  |  |  |  |  |  |  |  |  |
| Coffee |  |  |  |  |  |  |  |  |  |  |  |  |  |  |  |  |  |  |  |  |  |  |  |  |  |  |  |  |  |  |  |  |  |  |  |  |  |  |  |  |  |  |  |
| Tea & coffee |  |  |  |  |  |  |  |  |  |  |  |  |  |  |  |  |  |  |  |  |  |  |  |  |  |  |  |  |  |  |  |  |  |  |  |  |  |  |  |  |  |  |  |
| Low energy drinks |  |  |  |  |  |  |  |  |  |  |  |  |  |  |  |  |  |  |  |  |  |  |  |  |  |  |  |  |  |  |  |  |  |  |  |  |  |  |  |  |  |  |  |
| Fruit juice |  |  |  |  |  |  |  |  |  |  |  |  |  |  |  |  |  |  |  |  |  |  |  |  |  |  |  |  |  |  |  |  |  |  |  |  |  |  |  |  |  |  |  |
| Artificial juice (sugar-free) |  |  |  |  |  |  |  |  |  |  |  |  |  |  |  |  |  |  |  |  |  |  |  |  |  |  |  |  |  |  |  |  |  |  |  |  |  |  |  |  |  |  |  |
| Artificial juice (sugar) |  |  |  |  |  |  |  |  |  |  |  |  |  |  |  |  |  |  |  |  |  |  |  |  |  |  |  |  |  |  |  |  |  |  |  |  |  |  |  |  |  |  |  |
| Fluids (non-alcoholic) |  |  |  |  |  |  |  |  |  |  |  |  |  |  |  |  |  |  |  |  |  |  |  |  |  |  |  |  |  |  |  |  |  |  |  |  |  |  |  |  |  |  |  |
| **COMPOSITION** |  |  |  |  |  |  |  |  |  |  |  |  |  |  |  |  |  |  |  |  |  |  |  |  |  |  |  |  |  |  |  |  |  |  |  |  |  |  |  |  |  |  |  |
| Dietary fiber |  |  |  |  |  |  |  |  |  |  |  |  |  |  |  |  |  |  |  |  |  |  |  |  |  |  |  |  |  |  |  |  |  |  |  |  |  |  |  |  |  |  |  |
| Macronutrient ratio |  |  |  |  |  |  |  |  |  |  |  |  |  |  |  |  |  |  |  |  |  |  |  |  |  |  |  |  |  |  |  |  |  |  |  |  |  |  |  |  |  |  |  |
| Total carbohydrate |  |  |  |  |  |  |  |  |  |  |  |  |  |  |  |  |  |  |  |  |  |  |  |  |  |  |  |  |  |  |  |  |  |  |  |  |  |  |  |  |  |  |  |
| Total fat |  |  |  |  |  |  |  |  |  |  |  |  |  |  |  |  |  |  |  |  |  |  |  |  |  |  |  |  |  |  |  |  |  |  |  |  |  |  |  |  |  |  |  |
| PUFA/MUFA: SFA ratio |  |  |  |  |  |  |  |  |  |  |  |  |  |  |  |  |  |  |  |  |  |  |  |  |  |  |  |  |  |  |  |  |  |  |  |  |  |  |  |  |  |  |  |
| Omega-3 |  |  |  |  |  |  |  |  |  |  |  |  |  |  |  |  |  |  |  |  |  |  |  |  |  |  |  |  |  |  |  |  |  |  |  |  |  |  |  |  |  |  |  |
| Trans fat |  |  |  |  |  |  |  |  |  |  |  |  |  |  |  |  |  |  |  |  |  |  |  |  |  |  |  |  |  |  |  |  |  |  |  |  |  |  |  |  |  |  |  |
| SFA |  |  |  |  |  |  |  |  |  |  |  |  |  |  |  |  |  |  |  |  |  |  |  |  |  |  |  |  |  |  |  |  |  |  |  |  |  |  |  |  |  |  |  |
| Cholesterol |  |  |  |  |  |  |  |  |  |  |  |  |  |  |  |  |  |  |  |  |  |  |  |  |  |  |  |  |  |  |  |  |  |  |  |  |  |  |  |  |  |  |  |
| Empty calories |  |  |  |  |  |  |  |  |  |  |  |  |  |  |  |  |  |  |  |  |  |  |  |  |  |  |  |  |  |  |  |  |  |  |  |  |  |  |  |  |  |  |  |
| Sodium |  |  |  |  |  |  |  |  |  |  |  |  |  |  |  |  |  |  |  |  |  |  |  |  |  |  |  |  |  |  |  |  |  |  |  |  |  |  |  |  |  |  |  |
| Calcium |  |  |  |  |  |  |  |  |  |  |  |  |  |  |  |  |  |  |  |  |  |  |  |  |  |  |  |  |  |  |  |  |  |  |  |  |  |  |  |  |  |  |  |
| Iron |  |  |  |  |  |  |  |  |  |  |  |  |  |  |  |  |  |  |  |  |  |  |  |  |  |  |  |  |  |  |  |  |  |  |  |  |  |  |  |  |  |  |  |
| Vitamin C |  |  |  |  |  |  |  |  |  |  |  |  |  |  |  |  |  |  |  |  |  |  |  |  |  |  |  |  |  |  |  |  |  |  |  |  |  |  |  |  |  |  |  |
| Multivitamin/supplement use |  |  |  |  |  |  |  |  |  |  |  |  |  |  |  |  |  |  |  |  |  |  |  |  |  |  |  |  |  |  |  |  |  |  |  |  |  |  |  |  |  |  |  |

**Supplemental Figure S1.** Heatmap presenting the representation and classification of dietary components in the included indexes

(adapted from ^141^). Shading indicates that the dietary component was included in the index. Classification of dietary components as follows: Green, higher intake (≥ recommendations) scored favorably; Red, lower intake (≤ recommendations) scored favorably; Orange, moderate intake scored favorably

References

1. Kaluza J, Harris H, Melhus H, Michaëlsson K, Wolk A. Questionnaire-Based Anti-Inflammatory Diet Index as a Predictor of Low-Grade Systemic Inflammation. *Antioxid Redox Signal*. Jan 1 2018;28(1):78-84. doi:10.1089/ars.2017.7330

2. Byrd DA, Judd SE, Flanders WD, Hartman TJ, Fedirko V, Bostick RM. Development and Validation of Novel Dietary and Lifestyle Inflammation Scores. *The Journal of Nutrition*. 2019/12/01/ 2019;149(12):2206-2218. doi:<https://doi.org/10.1093/jn/nxz165>

3. Howard VJ, Cushman M, Pulley L, et al. The reasons for geographic and racial differences in stroke study: objectives and design. *Neuroepidemiology*. 2005;25(3):135-43. doi:10.1159/000086678

4. Gong Z, Xie D, Deng Z, et al. The PPAR{gamma} Pro12Ala polymorphism and risk for incident sporadic colorectal adenomas. *Carcinogenesis*. Mar 2005;26(3):579-85. doi:10.1093/carcin/bgh343

5. Daniel CR, Bostick RM, Flanders WD, et al. TGF-alpha expression as a potential biomarker of risk within the normal-appearing colorectal mucosa of patients with and without incident sporadic adenoma. *Cancer Epidemiol Biomarkers Prev*. Jan 2009;18(1):65-73. doi:10.1158/1055-9965.Epi-08-0732

6. Yang B, Gross MD, Fedirko V, McCullough ML, Bostick RM. Effects of calcium supplementation on biomarkers of inflammation and oxidative stress in colorectal adenoma patients: a randomized controlled trial. *Cancer Prev Res (Phila)*. Nov 2015;8(11):1069-75. doi:10.1158/1940-6207.Capr-15-0168

7. Tabung FK, Smith-Warner SA, Chavarro JE, et al. Development and Validation of an Empirical Dietary Inflammatory Index. *J Nutr*. Aug 2016;146(8):1560-70. doi:10.3945/jn.115.228718

8. Tabung FK, Smith-Warner SA, Chavarro JE, et al. An Empirical Dietary Inflammatory Pattern Score Enhances Prediction of Circulating Inflammatory Biomarkers in Adults. *The Journal of Nutrition*. 2017/08/01/ 2017;147(8):1567-1577. doi:<https://doi.org/10.3945/jn.117.248377>

9. Colditz GA, Hankinson SE. The Nurses' Health Study: lifestyle and health among women. *Nat Rev Cancer*. May 2005;5(5):388-96. doi:10.1038/nrc1608

10. Rimm EB, Giovannucci EL, Stampfer MJ, Colditz GA, Litin LB, Willett WC. Reproducibility and validity of an expanded self-administered semiquantitative food frequency questionnaire among male health professionals. *Am J Epidemiol*. May 15 1992;135(10):1114-26; discussion 1127-36. doi:10.1093/oxfordjournals.aje.a116211

11. Riboldi BP, Luft VC, Bracco PA, et al. The inflammatory food index and its association with weight gain and incidence of diabetes: Longitudinal Study of Adult Health (ELSA-Brasil). *Nutrition, Metabolism and Cardiovascular Diseases*. 2022;32(3):675-683. doi:<https://dx.doi.org/10.1016/j.numecd.2021.12.022>

12. Schmidt MI, Duncan BB, Mill JG, et al. Cohort Profile: Longitudinal Study of Adult Health (ELSA-Brasil). *International Journal of Epidemiology*. 2015;44(1):68-75. doi:10.1093/ije/dyu027

13. Azevedo-Garcia LG, Torres-Leal FL, Aristizabal JC, Berg G, Carvalho HB, De Moraes AC. Reliability and Validity Estimate of the Pro-Inflammatory/Anti-Inflammatory Food Intake Score in South American Pediatric Population: SAYCARE Study. *International Journal of Environmental Research and Public Health*. 2023;20(2). doi:10.3390/ijerph20021038

14. Carvalho HB, Moreno LA, Silva AM, et al. Design and Objectives of the South American Youth/Child Cardiovascular and Environmental (SAYCARE) Study. *Obesity*. 2018/03/01 2018;26(S1):S5-S13. doi:<https://doi.org/10.1002/oby.22117>

15. Akbaraly TN, Shipley MJ, Ferrie JE, et al. Long-term Adherence to Healthy Dietary Guidelines and Chronic Inflammation in the Prospective Whitehall II Study. *The American Journal of Medicine*. 2015;128(2):152-160.e4. doi:10.1016/j.amjmed.2014.10.002

16. Akbaraly TN, Ferrie JE, Berr C, et al. Alternative Healthy Eating Index and mortality over 18 y of follow-up: results from the Whitehall II cohort123. *The American Journal of Clinical Nutrition*. 2011/07/01/ 2011;94(1):247-253. doi:<https://doi.org/10.3945/ajcn.111.013128>

17. McCullough ML, Feskanich D, Stampfer MJ, et al. Diet quality and major chronic disease risk in men and women: moving toward improved dietary guidance. *The American Journal of Clinical Nutrition*. 2002;76(6):1261-1271. doi:10.1093/ajcn/76.6.1261

18. Aljuraiban GS, Gibson R, Al-Freeh L, et al. Associations Among Plant-Based Dietary Indexes, the Dietary Inflammatory Index, and Inflammatory Potential in Female College Students In Saudi Arabia: A Cross-Sectional Study. *Journal of the Academy of Nutrition and Dietetics*. 2022;122(4):771-785.e8. doi:10.1016/j.jand.2021.08.111

19. Satija A, Bhupathiraju SN, Rimm EB, et al. Plant-Based Dietary Patterns and Incidence of Type 2 Diabetes in US Men and Women: Results from Three Prospective Cohort Studies. *PLOS Medicine*. 2016;13(6):e1002039. doi:10.1371/journal.pmed.1002039

20. Alkerwi A, Vernier C, Crichton GE, Sauvageot N, Shivappa N, Hébert JR. Cross-comparison of diet quality indices for predicting chronic disease risk: findings from the Observation of Cardiovascular Risk Factors in Luxembourg (ORISCAV-LUX) study. *Br J Nutr*. Jan 28 2015;113(2):259-69. doi:10.1017/s0007114514003456

21. Alkerwi Aa, Sauvageot N, Donneau A-F, et al. First nationwide survey on cardiovascular risk factors in Grand-Duchy of Luxembourg (ORISCAV-LUX). *BMC Public Health*. 2010/08/10 2010;10(1):468. doi:10.1186/1471-2458-10-468

22. Fung TT. Adherence to a DASH-Style Diet and Risk of Coronary Heart Disease and Stroke in Women. *Archives of Internal Medicine*. 2008;168(7):713. doi:10.1001/archinte.168.7.713

23. Kim S, Haines PS, Siega-Riz AM, Popkin BM. The Diet Quality Index-International (DQI-I) Provides an Effective Tool for Cross-National Comparison of Diet Quality as Illustrated by China and the United States. *The Journal of Nutrition*. 2003;133(11):3476-3484. doi:10.1093/jn/133.11.3476

24. Trichopoulou A, Costacou T, Bamia C, Trichopoulos D. Adherence to a Mediterranean Diet and Survival in a Greek Population. *New England Journal of Medicine*. 2003;348(26):2599-2608. doi:10.1056/nejmoa025039

25. Alkerwi Aa, Sauvageot N, Nau A, et al. Population compliance with national dietary recommendations and its determinants: findings from the ORISCAV-LUX study. *British Journal of Nutrition*. 2012;108(11):2083-2092. doi:10.1017/S0007114512000232

26. Aroke D, Folefac E, Shi N, Jin Q, Clinton SK, Tabung FK. Inflammatory and Insulinemic Dietary Patterns: Influence on Circulating Biomarkers and Prostate Cancer Risk. *Cancer Prev Res (Phila)*. Oct 2020;13(10):841-852. doi:10.1158/1940-6207.Capr-20-0236

27. Gohagan JK, Prorok PC, Hayes RB, Kramer BS. The Prostate, Lung, Colorectal and Ovarian (PLCO) Cancer Screening Trial of the National Cancer Institute: history, organization, and status. *Control Clin Trials*. Dec 2000;21(6 Suppl):251s-272s. doi:10.1016/s0197-2456(00)00097-0

28. Arouca A, Michels N, Moreno LA, et al. Associations between a Mediterranean diet pattern and inflammatory biomarkers in European adolescents. *Eur J Nutr*. Aug 2018;57(5):1747-1760. doi:10.1007/s00394-017-1457-4

29. Moreno L, De Henauw S, Gonzalez-Gross M, et al. Design and implementation of the healthy lifestyle in Europe by nutrition in adolescence cross-sectional study. *International journal of obesity*. 2008;32(5):S4-S11.

30. Trichopoulou A, Kouris-Blazos A, Wahlqvist ML, et al. Diet and overall survival in elderly people. *Bmj*. Dec 2 1995;311(7018):1457-60. doi:10.1136/bmj.311.7018.1457

31. Baden MY, Satija A, Hu FB, Huang T. Change in Plant-Based Diet Quality Is Associated with Changes in Plasma Adiposity-Associated Biomarker Concentrations in Women. *The Journal of Nutrition*. 2019/04/01/ 2019;149(4):676-686. doi:<https://doi.org/10.1093/jn/nxy301>

32. Bérard S, Morel S, Teasdale E, et al. Diet Quality Is Associated with Cardiometabolic Outcomes in Survivors of Childhood Leukemia. *Nutrients*. Jul 18 2020;12(7)doi:10.3390/nu12072137

33. Levy E, Samoilenko M, Morel S, et al. Cardiometabolic Risk Factors in Childhood, Adolescent and Young Adult Survivors of Acute Lymphoblastic Leukemia – A Petale Cohort. *Scientific Reports*. 2017/12/15 2017;7(1):17684. doi:10.1038/s41598-017-17716-0

34. Krebs-Smith SM, Pannucci TE, Subar AF, et al. Update of the Healthy Eating Index: HEI-2015. *Journal of the Academy of Nutrition and Dietetics*. 2018;118(9):1591-1602. doi:10.1016/j.jand.2018.05.021

35. Schröder H, Fitó M, Estruch R, et al. A short screener is valid for assessing Mediterranean diet adherence among older Spanish men and women. *J Nutr*. Jun 2011;141(6):1140-5. doi:10.3945/jn.110.135566

36. Bonaccio M, Costanzo S, Di Castelnuovo A, et al. Increased Adherence to a Mediterranean Diet Is Associated With Reduced Low-Grade Inflammation after a 12.7-Year Period: Results From the Moli-sani Study. *J Acad Nutr Diet*. May 2023;123(5):783-795.e7. doi:10.1016/j.jand.2022.12.005

37. Di Castelnuovo A, Costanzo S, Persichillo M, et al. Distribution of short and lifetime risks for cardiovascular disease in Italians. *European Journal of Preventive Cardiology*. 2012;19(4):723-730.

38. Carter SJ, Roberts MB, Salter J, Eaton CB. Relationship between Mediterranean Diet Score and atherothrombotic risk: Findings from the Third National Health and Nutrition Examination Survey (NHANES III), 1988–1994. *Atherosclerosis*. 2010/06/01/ 2010;210(2):630-636. doi:<https://doi.org/10.1016/j.atherosclerosis.2009.12.035>

39. Ezzati TM, Massey JT, Waksberg J, Chu A, Maurer KR. Sample design: Third National Health and Nutrition Examination Survey. *Vital Health Stat 2*. Sep 1992;(113):1-35.

40. Panagiotakos DB, Milias GA, Pitsavos C, Stefanadis C. MedDietScore: a computer program that evaluates the adherence to the Mediterranean dietary pattern and its relation to cardiovascular disease risk. *Comput Methods Programs Biomed*. Jul 2006;83(1):73-7. doi:10.1016/j.cmpb.2006.05.003

41. Cervo MMC, Scott D, Seibel MJ, et al. Adherence to Mediterranean diet and its associations with circulating cytokines, musculoskeletal health and incident falls in community-dwelling older men: The Concord Health and Ageing in Men Project. *Clin Nutr*. Dec 2021;40(12):5753-5763. doi:10.1016/j.clnu.2021.10.010

42. Cumming RG, Handelsman D, Seibel MJ, et al. Cohort Profile: the Concord Health and Ageing in Men Project (CHAMP). *Int J Epidemiol*. Apr 2009;38(2):374-8. doi:10.1093/ije/dyn071

43. Sofi F, Macchi C, Abbate R, Gensini GF, Casini A. Mediterranean diet and health status: an updated meta-analysis and a proposal for a literature-based adherence score. *Public Health Nutr*. Dec 2014;17(12):2769-82. doi:10.1017/s1368980013003169

44. Chan R, Yu B, Leung J, Lee JS, Woo J. Association of dietary patterns with serum high-sensitivity C-reactive protein level in community-dwelling older adults. *Clin Nutr ESPEN*. 2019;31:38-47. doi:10.1016/j.clnesp.2019.03.004

45. Morris MC, Tangney CC, Wang Y, et al. MIND diet slows cognitive decline with aging. *Alzheimers Dement*. Sep 2015;11(9):1015-22. doi:10.1016/j.jalz.2015.04.011

46. Willcox BJ, Willcox DC, Todoriki H, et al. Caloric restriction, the traditional Okinawan diet, and healthy aging: the diet of the world's longest-lived people and its potential impact on morbidity and life span. *Ann N Y Acad Sci*. Oct 2007;1114:434-55. doi:10.1196/annals.1396.037

47. Dai J, Miller AH, Bremner JD, et al. Adherence to the Mediterranean Diet Is Inversely Associated With Circulating Interleukin-6 Among Middle-Aged Men. *Circulation*. 2008/01/15 2008;117(2):169-175. doi:10.1161/CIRCULATIONAHA.107.710699

48. Henderson WG, Eisen S, Goldberg J, True WR, Barnes JE, Vitek ME. The Vietnam Era Twin Registry: a resource for medical research. *Public Health Rep*. Jul-Aug 1990;105(4):368-73.

49. de Graaf MCG, Spooren CEGM, Hendrix EMB, et al. Diet Quality and Dietary Inflammatory Index in Dutch Inflammatory Bowel Disease and Irritable Bowel Syndrome Patients. *Nutrients*. 2022;14(9). doi:10.3390/nu14091945

50. van den Heuvel TR, Jonkers DM, Jeuring SF, et al. Cohort Profile: The Inflammatory Bowel Disease South Limburg Cohort (IBDSL). *Int J Epidemiol*. Apr 1 2017;46(2):e7. doi:10.1093/ije/dyv088

51. Ersryd A, Posserud I, Abrahamsson H, SimrÉN M. Subtyping the irritable bowel syndrome by predominant bowel habit: Rome II versus Rome III. *Alimentary Pharmacology & Therapeutics*. 2007/09/01 2007;26(6):953-961. doi:<https://doi.org/10.1111/j.1365-2036.2007.03422.x>

52. Looman M, Feskens EJM, de Rijk M, et al. Development and evaluation of the Dutch Healthy Diet index 2015. *Public Health Nutrition*. 2017;20(13):2289-2299. doi:10.1017/S136898001700091X

53. Dias JA, Wirfält E, Drake I, et al. A high quality diet is associated with reduced systemic inflammation in middle-aged individuals. *Atherosclerosis*. 2015;238(1):38-44. doi:10.1016/j.atherosclerosis.2014.11.006

54. Berglund G, ElmstÅHl S, Janzon L, Larsson SA. Design and feasibility. *Journal of Internal Medicine*. 1993/01/01 1993;233(1):45-51. doi:<https://doi.org/10.1111/j.1365-2796.1993.tb00647.x>

55. Drake I, Gullberg B, Ericson U, et al. Development of a diet quality index assessing adherence to the Swedish nutrition recommendations and dietary guidelines in the Malmö Diet and Cancer cohort. *Public Health Nutr*. May 2011;14(5):835-45. doi:10.1017/s1368980010003848

56. English CJ, Lohning AE, Mayr HL, Jones M, MacLaughlin H, Reidlinger DP. The association between dietary quality scores with C-reactive protein and novel biomarkers of inflammation platelet-activating factor and lipoprotein-associated phospholipase A2: a cross-sectional study. *Nutrition & Metabolism*. 2023;20(1):1-13. doi:10.1186/s12986-023-00756-x

57. Günther AL, Liese AD, Bell RA, et al. Association between the dietary approaches to hypertension diet and hypertension in youth with diabetes mellitus. *Hypertension*. Jan 2009;53(1):6-12. doi:10.1161/hypertensionaha.108.116665

58. Roy R, Hebden L, Rangan A, Allman-Farinelli M. The development, application, and validation of a Healthy eating index for Australian Adults (HEIFA—2013). *Nutrition*. 2016;32(4):432-440.

59. Martínez-González MA, de la Fuente-Arrillaga C, Nunez-Cordoba JM, et al. Adherence to Mediterranean diet and risk of developing diabetes: prospective cohort study. *Bmj*. Jun 14 2008;336(7657):1348-51. doi:10.1136/bmj.39561.501007.BE

60. Fargnoli JL, Fung TT, Olenczuk DM, Chamberland JP, Hu FB, Mantzoros CS. Adherence to healthy eating patterns is associated with higher circulating total and high-molecular-weight adiponectin and lower resistin concentrations in women from the Nurses' Health Study. *Am J Clin Nutr*. 2008;88(5):1213-24. doi:10.3945/ajcn.2008.26480

61. Fernández-Barrés S, Vrijheid M, Manzano-Salgado CB, et al. The Association of Mediterranean Diet during Pregnancy with Longitudinal Body Mass Index Trajectories and Cardiometabolic Risk in Early Childhood. *J Pediatr*. Mar 2019;206:119-127.e6. doi:10.1016/j.jpeds.2018.10.005

62. Guxens M, Ballester F, Espada M, et al. Cohort Profile: The INMA—INfancia y Medio Ambiente—(Environment and Childhood) Project. *International Journal of Epidemiology*. 2012;41(4):930-940. doi:10.1093/ije/dyr054

63. Buckland G, González CA, Agudo A, et al. Adherence to the Mediterranean Diet and Risk of Coronary Heart Disease in the Spanish EPIC Cohort Study. *American Journal of Epidemiology*. 2009;170(12):1518-1529. doi:10.1093/aje/kwp282

64. Ford ES, Mokdad AH, Liu S. Healthy Eating Index and C-reactive protein concentration: findings from the National Health and Nutrition Examination Survey III, 1988-1994. *Eur J Clin Nutr*. 2005;59(2):278-83. doi:10.1038/sj.ejcn.1602070

65. Kennedy ET, Ohls J, Carlson S, Fleming K. The Healthy Eating Index: design and applications. *J Am Diet Assoc*. Oct 1995;95(10):1103-8. doi:10.1016/s0002-8223(95)00300-2

66. Fung TT, McCullough ML, Newby PK, et al. Diet-quality scores and plasma concentrations of markers of inflammation and endothelial dysfunction. *American Journal of Clinical Nutrition*. 2005;82(1):163-173.

67. Kant AK. A Prospective Study of Diet Quality and Mortality in Women. *JAMA*. 2000;283(16):2109. doi:10.1001/jama.283.16.2109

68. González-Ortiz A, Xu H, Avesani CM, et al. Plant-based diets, insulin sensitivity and inflammation in elderly men with chronic kidney disease. *Journal of Nephrology*. 2020/10/01 2020;33(5):1091-1101. doi:10.1007/s40620-020-00765-6

69. Franzon K, Zethelius B, Cederholm T, Kilander L. Modifiable Midlife Risk Factors, Independent Aging, and Survival in Older Men: Report on Long-Term Follow-Up of the Uppsala Longitudinal Study of Adult Men Cohort. *Journal of the American Geriatrics Society*. 2015/05/01 2015;63(5):877-885. doi:<https://doi.org/10.1111/jgs.13352>

70. Kim H, Caulfield LE, Garcia-Larsen V, et al. Plant-Based Diets and Incident CKD and Kidney Function. *Clinical Journal of the American Society of Nephrology*. 2019;14(5)

71. Hayuningtyas A, Dewi YA, Octavia L, Pulungan A, Agustina R. Dietary quality score is positively associated with serum adiponectin level in Indonesian preschool-age children living in the urban area of Jakarta. *PLoS One*. 2021;16(2):e0246234. doi:10.1371/journal.pone.0246234

72. Dudung A, Victor T, Helda K, Fiastuti W, Rina A. Inadequate dietary α-linolenic acid intake among Indonesian pregnant women is associated with lower newborn weights in urban Jakarta. *Asia Pacific Journal of Clinical Nutrition*. 2017;26(S1):s9-s18. doi:10.6133/apjcn.062017.s1

73. Huang T, Tobias DK, Hruby A, Rifai N, Tworoger SS, Hu FB. An Increase in Dietary Quality Is Associated with Favorable Plasma Biomarkers of the Brain-Adipose Axis in Apparently Healthy US Women. *J Nutr*. May 2016;146(5):1101-8. doi:10.3945/jn.115.229666

74. Chiuve SE, Fung TT, Rimm EB, et al. Alternative dietary indices both strongly predict risk of chronic disease. *J Nutr*. Jun 2012;142(6):1009-18. doi:10.3945/jn.111.157222

75. Huang Y, Li X, Zhang T, et al. Associations of healthful and unhealthful plant-based diets with plasma markers of cardiometabolic risk. *Eur J Nutr*. 2023;62(6):2567-2579. doi:10.1007/s00394-023-03170-4

76. Kanerva N, Loo B-M, Eriksson JG, et al. Associations of the Baltic Sea diet with obesity-related markers of inflammation. *Annals of Medicine*. 2014;46(2):90-96. doi:10.3109/07853890.2013.870020

77. Kanerva N, Kaartinen NE, Schwab U, Lahti-Koski M, Männistö S. The Baltic Sea Diet Score: a tool for assessing healthy eating in Nordic countries. *Public Health Nutrition*. 2014;17(8):1697-1705. doi:10.1017/S1368980013002395

78. Kant AK, Graubard BI. A comparison of three dietary pattern indexes for predicting biomarkers of diet and disease. *J Am Coll Nutr*. Aug 2005;24(4):294-303. doi:10.1080/07315724.2005.10719477

79. Kant AK, Graubard BI, Schatzkin A. Dietary Patterns Predict Mortality in a National Cohort: The National Health Interview Surveys, 1987 and 1992. *The Journal of Nutrition*. 2004/07/01/ 2004;134(7):1793-1799. doi:<https://doi.org/10.1093/jn/134.7.1793>

80. Kennedy ET, Ohls J, Carlson S, Fleming K. The healthy eating index: Design and applications. *American Dietetic Association Journal of the American Dietetic Association*. Oct 1995

2023-11-28 1995;95(10):1103.

81. Kharaty S, Harrington JM, Millar SR, Perry IJ, Phillips CM. Plant-based dietary indices and biomarkers of chronic low-grade inflammation: a cross-sectional analysis of adults in Ireland. *Eur J Nutr*. 2023;62(8):3397-3410. doi:10.1007/s00394-023-03242-5

82. Kearney PM, Harrington JM, Mc Carthy VJC, Fitzgerald AP, Perry IJ. Cohort Profile: The Cork and Kerry Diabetes and Heart Disease Study. *International Journal of Epidemiology*. 2013;42(5):1253-1262. doi:10.1093/ije/dys131

83. Ko BJ, Park KH, Shin S, et al. Diet quality and diet patterns in relation to circulating cardiometabolic biomarkers. *Clin Nutr*. 2016;35(2):484-490. doi:10.1016/j.clnu.2015.03.022

84. Lahoz C, Castillo E, Mostaza JM, et al. Relationship of the Adherence to a Mediterranean Diet and Its Main Components with CRP Levels in the Spanish Population. *Nutrients*. Mar 20 2018;10(3)doi:10.3390/nu10030379

85. Salinero-Fort MÁ, de Burgos-Lunar C, Mostaza Prieto J, et al. Validating prediction scales of type 2 diabetes mellitus in Spain: the SPREDIA-2 population-based prospective cohort study protocol. *BMJ Open*. 2015;5(7):e007195. doi:10.1136/bmjopen-2014-007195

86. Li SX, Hodge AM, MacInnis RJ, et al. Inflammation-Related Marker Profiling of Dietary Patterns and All-cause Mortality in the Melbourne Collaborative Cohort Study. *J Nutr*. 2021;151(10):2908-2916. doi:10.1093/jn/nxab231

87. Milne RL, Fletcher AS, MacInnis RJ, et al. Cohort Profile: The Melbourne Collaborative Cohort Study (Health 2020). *Int J Epidemiol*. Dec 1 2017;46(6):1757-1757i. doi:10.1093/ije/dyx085

88. Trichopoulou A, Orfanos P, Norat T, et al. Modified Mediterranean diet and survival: EPIC-elderly prospective cohort study. *BMJ*. 2005;330(7498):991. doi:10.1136/bmj.38415.644155.8f

89. Li J, Demirel A, Azuero A, et al. Limited Association between the Total Healthy Eating Index-2015 Score and Cardiovascular Risk Factors in Individuals with Long-Standing Spinal Cord Injury: An Exploratory Study: An Exploratory Study. *J Acad Nutr Diet*. 2021;121(11):2260-2266. doi:10.1016/j.jand.2021.04.010

90. Li L, Wan Z, Geng T, et al. Associations of healthy dietary patterns with mortality among people with prediabetes. *Eur J Nutr*. 2023;62(3):1377-1387. doi:10.1007/s00394-022-03078-5

91. Matsunaga M, Hurwitz EL, Li D. Adequate intake of plant protein foods and moderate intake of animal protein foods are inversely associated with C-reactive protein in US adults with diabetes: A cross-sectional study with National Health and Nutrition Examination Survey. *Nutrition*. 2021;89:111276. doi:10.1016/j.nut.2021.111276

92. Matsunaga M, Hurwitz EL, Li D. Development and Evaluation of a Dietary Approaches to Stop Hypertension Dietary Index with Calorie-Based Standards in Equivalent Units: A Cross-Sectional Study with 24-Hour Dietary Recalls from Adult Participants in the National Health and Nutrition Examination Survey 2007-2010. *Journal of the Academy of Nutrition and Dietetics*. 2018/01/01/ 2018;118(1):62-73.e4. doi:<https://doi.org/10.1016/j.jand.2017.03.010>

93. Mattei J, Sotos-Prieto M, Bigornia SJ, Noel SE, Tucker KL. The Mediterranean Diet Score Is More Strongly Associated with Favorable Cardiometabolic Risk Factors over 2 Years Than Other Diet Quality Indexes in Puerto Rican Adults. *J Nutr*. 2017;147(4):661-669. doi:10.3945/jn.116.245431

94. Tucker KL, Mattei J, Noel SE, et al. The Boston Puerto Rican Health Study, a longitudinal cohort study on health disparities in Puerto Rican adults: challenges and opportunities. *BMC Public Health*. Mar 1 2010;10:107. doi:10.1186/1471-2458-10-107

95. Mattei J, Bhupathiraju S, Tucker KL. Higher adherence to a diet score based on American Heart Association recommendations is associated with lower odds of allostatic load and metabolic syndrome in Puerto Rican adults. *J Nutr*. Nov 2013;143(11):1753-9. doi:10.3945/jn.113.180141

96. Guenther PM, Kirkpatrick SI, Reedy J, et al. The Healthy Eating Index-2010 Is a Valid and Reliable Measure of Diet Quality According to the 2010 Dietary Guidelines for Americans1, 2, 3. *The Journal of Nutrition*. 2014/03/01/ 2014;144(3):399-407. doi:<https://doi.org/10.3945/jn.113.183079>

97. Mears M, Tussing-Humphreys L, Cerwinske L, et al. Associations between alternate healthy eating index-2010, body composition, osteoarthritis severity, and interleukin-6 in older overweight and obese african american females with self-reported osteoarthritis. *Nutrients*. 2019;11(1):26. doi:<https://dx.doi.org/10.3390/nu11010026>

98. Millar SR, Navarro P, Harrington JM, et al. Dietary score associations with markers of chronic low-grade inflammation: a cross-sectional comparative analysis of a middle- to older-aged population. *European Journal of Nutrition*. 2022;doi:10.1007/s00394-022-02892-1

99. Millar SR, Navarro P, Harrington JM, Perry IJ, Phillips CM. Dietary Quality Determined by the Healthy Eating Index-2015 and Biomarkers of Chronic Low-Grade Inflammation: A Cross-Sectional Analysis in Middle-to-Older Aged Adults. *Nutrients*. 2021;13(1):222. doi:10.3390/nu13010222

100. Mirrafiei A, Ansari S, Jayedi A, Lesani A, Djafarian K, Shab-Bidar S. The association of meal-specific food-based dietary inflammatory index with cardiovascular risk factors and inflammation in a sample of Iranian adults. *BMC Endocrine Disorders*. 2023/01/10 2023;23(1):10. doi:10.1186/s12902-023-01265-x

101. Salari-Moghaddam A, Keshteli AH, Afshar H, Esmaillzadeh A, Adibi P. Empirically derived food-based dietary inflammatory index is associated with increased risk of psychological disorders in women. *Nutr Neurosci*. Apr 2021;24(4):260-268. doi:10.1080/1028415x.2019.1621044

102. Monfort-Pires M, Folchetti LD, Previdelli AN, Siqueira-Catania A, de Barros CR, Ferreira SR. Healthy Eating Index is associated with certain markers of inflammation and insulin resistance but not with lipid profile in individuals at cardiometabolic risk. *Appl Physiol Nutr Metab*. 2014;39(4):497-502. doi:10.1139/apnm-2013-0279

103. Moradi M, Daneshzad E, Najafabadi MM, Bellissimo N, Suitor K, Azadbakht L. Association between adherence to the Mediterranean diet and renal function biomarkers and cardiovascular risk factors among diabetic patients with nephropathy. *Clin Nutr ESPEN*. Dec 2020;40:156-163. doi:10.1016/j.clnesp.2020.09.032

104. Nilsson A, Halvardsson P, Kadi F. Adherence to DASH-Style Dietary Pattern Impacts on Adiponectin and Clustered Metabolic Risk in Older Women. *Nutrients*. 2019;11(4)doi:10.3390/nu11040805

105. Piccand E, Vollenweider P, Guessous I, Marques-Vidal P. Association between dietary intake and inflammatory markers: results from the CoLaus study. *Public Health Nutrition*. 2019;22(3):498-505. doi:10.1017/S1368980018002355

106. Firmann M, Mayor V, Vidal PM, et al. The CoLaus study: a population-based study to investigate the epidemiology and genetic determinants of cardiovascular risk factors and metabolic syndrome. *BMC Cardiovasc Disord*. Mar 17 2008;8:6. doi:10.1186/1471-2261-8-6

107. Piccirillo F, Miano N, Goffredo C, et al. Impact of Mediterranean diet on metabolic and inflammatory status of patients with polyvascular atherosclerotic disease. *Nutr Metab Cardiovasc Dis*. Jan 2022;32(1):117-124. doi:10.1016/j.numecd.2021.09.032

108. Martínez-González MA, Fernández-Jarne E, Serrano-Martínez M, Marti A, Martinez JA, Martín-Moreno JM. Mediterranean diet and reduction in the risk of a first acute myocardial infarction: an operational healthy dietary score. *Eur J Nutr*. Aug 2002;41(4):153-60. doi:10.1007/s00394-002-0370-6

109. Pocovi-Gerardino G, Correa-Rodríguez M, Callejas-Rubio JL, et al. Beneficial effect of Mediterranean diet on disease activity and cardiovascular risk in systemic lupus erythematosus patients: a cross-sectional study. *Rheumatology (Oxford)*. Jan 5 2021;60(1):160-169. doi:10.1093/rheumatology/keaa210

110. Pourreza S, Khademi Z, Mirzababaei A, et al. Association of plant-based diet index with inflammatory markers and sleep quality in overweight and obese female adults: A cross-sectional study. *Int J Clin Pract*. 2021;75(9):e14429. doi:10.1111/ijcp.14429

111. Rostgaard-Hansen AL, Lau CJ, Halkjær J, Olsen A, Toft U. An updated validation of the Dietary Quality Score: associations with risk factors for cardiometabolic diseases in a Danish population. *European Journal of Nutrition*. 2023/06/01 2023;62(4):1647-1656. doi:10.1007/s00394-023-03100-4

112. Petersen KEN, Halkjær J, Loft S, Tjønneland A, Olsen A. Cohort profile and representativeness of participants in the Diet, Cancer and Health—Next Generations cohort study. *European Journal of Epidemiology*. 2022/01/01 2022;37(1):117-127. doi:10.1007/s10654-021-00832-7

113. Toft U, Kristoffersen L, Lau C, Borch-Johnsen K, Jørgensen T. The Dietary Quality Score: Validation and association with cardiovascular risk factors: The Inter99 study. *European journal of clinical nutrition*. 02/01 2007;61:270-8. doi:10.1038/sj.ejcn.1602503

114. Sabia F, Borgo A, Lugo A, et al. Evaluation of Simplified Diet Scores Related to C-Reactive Protein in Heavy Smokers Undergoing Lung Cancer Screening. *Nutrients*. 2022;14(20). doi:10.3390/nu14204312

115. Savard C, Lemieux S, Plante A-S, et al. Longitudinal changes in circulating concentrations of inflammatory markers throughout pregnancy: are there associations with diet and weight status? *Applied Physiology, Nutrition, and Metabolism*. 2022/03/01 2021;47(3):287-295. doi:10.1139/apnm-2021-0395

116. Savard C, Lemieux S, Lafrenière J, Laramée C, Robitaille J, Morisset A-S. Validation of a self-administered web-based 24-hour dietary recall among pregnant women. *BMC Pregnancy and Childbirth*. 2018/04/23 2018;18(1):112. doi:10.1186/s12884-018-1741-1

117. Willett WC, Sacks F, Trichopoulou A, et al. Mediterranean diet pyramid: a cultural model for healthy eating. *Am J Clin Nutr*. Jun 1995;61(6 Suppl):1402s-1406s. doi:10.1093/ajcn/61.6.1402S

118. Serrano-Martinez M, Palacios M, Martinez-Losa E, et al. A Mediterranean dietary style influences TNF-alpha and VCAM-1 coronary blood levels in unstable angina patients. *Eur J Nutr*. Sep 2005;44(6):348-54. doi:10.1007/s00394-004-0532-9

119. Sood S, Feehan J, Itsiopoulos C, et al. Higher Adherence to a Mediterranean Diet Is Associated with Improved Insulin Sensitivity and Selected Markers of Inflammation in Individuals Who Are Overweight and Obese without Diabetes. *Nutrients*. Oct 21 2022;14(20)doi:10.3390/nu14204437

120. Panagiotakos DB, Pitsavos C, Stefanadis C. Dietary patterns: A Mediterranean diet score and its relation to clinical and biological markers of cardiovascular disease risk. *Nutrition, Metabolism and Cardiovascular Diseases*. 2006;16(8):559-568. doi:10.1016/j.numecd.2005.08.006

121. Tabung FK, Wang W, Fung TT, et al. Development and validation of empirical indices to assess the insulinaemic potential of diet and lifestyle. *Br J Nutr*. Nov 28 2016;116(10):1787-1798. doi:10.1017/s0007114516003755

122. Tertsunen H-M, Hantunen S, Tuomainen T-P, Salonen JT, Virtanen JK. A healthy Nordic diet score and risk of incident CHD among men: the Kuopio Ischaemic Heart Disease Risk Factor Study. *British Journal of Nutrition*. 2022;127(4):599-606. doi:10.1017/S0007114521001227

123. Salonen JT. Is there a continuing need for longitudinal epidemiologic research? The Kuopio Ischaemic Heart Disease Risk Factor Study. *Ann Clin Res*. 1988;20(1-2):46-50.

124. Vagianos K, Shafer LA, Witges K, et al. Association Between Change in Inflammatory Aspects of Diet and Change in IBD-related Inflammation and Symptoms Over 1 Year: The Manitoba Living With IBD Study. *Inflammatory Bowel Diseases*. 2021;27(2):190-202. doi:10.1093/ibd/izaa052

125. Vahid F, Jalili M, Rahmani W, Nasiri Z, Bohn T. A Higher Healthy Eating Index Is Associated with Decreased Markers of Inflammation and Lower Odds for Being Overweight/Obese Based on a Case-Control Study. *Nutrients*. 2022;14(23). doi:10.3390/nu14235127

126. Vahid F, Hoge A, Hébert JR, et al. Association of diet quality indices with serum and metabolic biomarkers in participants of the ORISCAV-LUX-2 study. *European Journal of Nutrition*. 2023;62(5):2063-2085. doi:10.1007/s00394-023-03095-y

127. van der Pligt PF, Ebrahimi S, Kuswara K, et al. Associations of adherence to the DASH diet and Mediterranean diet with maternal c-reactive protein levels during pregnancy. *Nutr Metab Cardiovasc Dis*. 2024;34(3):672-680. doi:10.1016/j.numecd.2023.11.001

128. De Guingand DL, Ellery SJ, Davies-Tuck ML, Dickinson H. Creatine and pregnancy outcomes, a prospective cohort study in low-risk pregnant women: study protocol. *BMJ Open*. 2019;9(1):e026756. doi:10.1136/bmjopen-2018-026756

129. Vicente B, Bastos A, Melo C, Aquino R, Ribeiro S. Correlation Between Different Dietary Indexes, and Their Association with An Anti-inflammatory Biomarker in Older Adults: An Exploratory Study. *European Journal of Geriatrics and Gerontology*. 09/12 2023;5:238-245. doi:10.4274/ejgg.galenos.2023.2022-10-5

130. Viscogliosi G, Cipriani E, Liguori ML, et al. Mediterranean dietary pattern adherence: associations with prediabetes, metabolic syndrome, and related microinflammation. *Metab Syndr Relat Disord*. Jun 2013;11(3):210-6. doi:10.1089/met.2012.0168

131. Martínez-González MA, García-Arellano A, Toledo E, et al. A 14-item Mediterranean diet assessment tool and obesity indexes among high-risk subjects: the PREDIMED trial. *PLoS One*. 2012;7(8):e43134. doi:10.1371/journal.pone.0043134

132. Waldeyer C, Brunner FJ, Braetz J, et al. Adherence to Mediterranean diet, high-sensitive C-reactive protein, and severity of coronary artery disease: Contemporary data from the INTERCATH cohort. *Atherosclerosis*. Aug 2018;275:256-261. doi:10.1016/j.atherosclerosis.2018.06.877

133. Stewart RA, Wallentin L, Benatar J, et al. Dietary patterns and the risk of major adverse cardiovascular events in a global study of high-risk patients with stable coronary heart disease. *Eur Heart J*. Jul 1 2016;37(25):1993-2001. doi:10.1093/eurheartj/ehw125

134. Wang YB, Page AJ, Gill TK, Melaku YA. The association between diet quality, plant-based diets, systemic inflammation, and mortality risk: findings from NHANES. *Eur J Nutr*. 2023;62(7):2723-2737. doi:10.1007/s00394-023-03191-z

135. Martínez-González MA, Sánchez-Tainta A, Corella D, et al. A provegetarian food pattern and reduction in total mortality in the Prevención con Dieta Mediterránea (PREDIMED) study1234. *The American Journal of Clinical Nutrition*. 2014/07/01/ 2014;100:320S-328S. doi:<https://doi.org/10.3945/ajcn.113.071431>

136. Weber KS, Lang A, Strassburger K, et al. Association of dietary patterns with diabetes-related comorbidities varies among diabetes endotypes. *Nutrition, Metabolism and Cardiovascular Diseases*. 2024;doi:<https://dx.doi.org/10.1016/j.numecd.2023.12.026>

137. Zaharia OP, Strassburger K, Strom A, et al. Risk of diabetes-associated diseases in subgroups of patients with recent-onset diabetes: a 5-year follow-up study. *The Lancet Diabetes & Endocrinology*. 2019;7(9):684-694. doi:10.1016/S2213-8587(19)30187-1

138. Whalen KA, McCullough ML, Flanders WD, Hartman TJ, Judd S, Bostick RM. Paleolithic and Mediterranean Diet Pattern Scores Are Inversely Associated with Biomarkers of Inflammation and Oxidative Balance in Adults. *J Nutr*. 2016;146(6):1217-26. doi:10.3945/jn.115.224048

139. Whalen KA, McCullough M, Flanders WD, Hartman TJ, Judd S, Bostick RM. Paleolithic and Mediterranean diet pattern scores and risk of incident, sporadic colorectal adenomas. *Am J Epidemiol*. Dec 1 2014;180(11):1088-97. doi:10.1093/aje/kwu235

140. McCullough ML, Feskanich D, Stampfer MJ, et al. Adherence to the Dietary Guidelines for Americans and risk of major chronic disease in women. *Am J Clin Nutr*. Nov 2000;72(5):1214-22. doi:10.1093/ajcn/72.5.1214

141. Miller V, Webb P, Micha R, Mozaffarian D. Defining diet quality: a synthesis of dietary quality metrics and their validity for the double burden of malnutrition. *Lancet Planet Health*. Aug 2020;4(8):e352-e370. doi:10.1016/s2542-5196(20)30162-5
